# Molecular functions of the double‐sided and inverted ubiquitin‐interacting motif found in *Xenopus tropicalis* cryptochrome 6

**DOI:** 10.1111/dgd.12852

**Published:** 2023-05-16

**Authors:** Keiko Okano, Hiroaki Otsuka, Marika Nakagawa, Toshiyuki Okano

**Affiliations:** ^1^ Department of Electrical Engineering and Bioscience, Graduate School of Sciences and Engineering Waseda University, TWIns Tokyo Japan

**Keywords:** cryptochrome, nucleolus, photoreception, ubiquitin‐interacting motif, *Xenopus tropicalis*

## Abstract

Cryptochromes (CRYs) are multifunctional molecules that act as a circadian clock oscillating factor, a blue‐light sensor, and a light‐driven magnetoreceptor. *Cry* genes are classified into several groups based on the evolutionary relationships. *Cryptochrome 6* gene (*Cry6*) is present in invertebrates and lower vertebrates such as amphibians and fishes. Here we identified a *Cry6* ortholog in *Xenopus tropicalis* (*XtCry6*). XtCRY6 retains a conserved long N‐terminal extension (termed CRY N‐terminal extension; CNE) that is not found in any CRY in the other groups. A structural prediction suggested that CNE contained unique structures; a tetrahelical fold structure topologically related to KaiA/RbsU domain, overlapping nuclear‐ and nucleolar‐localizing signals (NLS/NoLS), and a novel motif (termed DI‐UIM) overlapping a double‐sided ubiquitin‐interacting motif (DUIM) and an inverted ubiquitin‐interacting motif (IUIM). Potential activities of the NLS/NoLS and DI‐UIM were examined to infer the molecular function of XtCRY6. GFP‐NLS/NoLS fusion protein exogenously expressed in HEK293 cells was mostly observed in the nucleolus, while GFP‐XtCRY6 was observed in the cytoplasm. A glutathione *S*‐transferase (GST) pull‐down assay suggested that the DI‐UIM physically interacts with polyubiquitin. Consistently, protein docking simulations implied that XtCRY6 DI‐UIM binds two ubiquitin molecules in a relationship of a twofold rotational symmetry with the symmetry axis parallel or perpendicular to the DI‐UIM helix. These results strongly suggested that XtCRY6 does not function as a circadian transcriptional repressor and that it might have another function such as photoreceptive molecule regulating light‐dependent protein degradation or gene expression through a CNE‐mediated interaction with ubiquitinated proteins in the cytoplasm and/or nucleolus.

## Graphical abstract

To infer the molecular function of uncharacterized group of cryptochrome CRY6 in *Xenopus tropicalis*, we performed in silico structure prediction. XtCRY6 retains a long N‐terminal extension (CNE), and structural prediction suggested that CNE contained unique structures, KaiA/RbsU‐like fold domain and a novel motif termed DI‐UIM composed of a double‐sided ubiquitin‐interacting motif (DUIM) and an inverted ubiquitin‐interacting motif (IUIM). We also investigated its temporal expression, cellular localization, and ubiquitin‐binding ability.

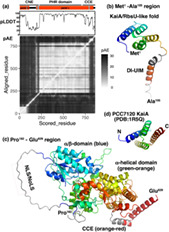

## 1 INTRODUCTION

Cryptochromes (CRY) form a large family with photorepair enzyme photolyases (Fortunato et al., 2015; Lin & Todo, 2005; Ozturk, 2017). Most cryptochromes bind a flavin adenine dinucleotide (FAD) chromophore that absorbs blue light to function as photosensors or light‐dependent magnetoreceptors (Ozturk, 2017; Wiltschko et al., 2021). Mammalian CRY1 and CRY2 are unique in that they are not activated by blue light but function as transcriptional repressors in the core oscillatory mechanism of the circadian clock (Takahashi, 2016). Avian CRY4 proteins have been analyzed by us and other groups and are presumed to be magnetoreceptors (Günther et al., 2018; Mitsui et al., 2015; Otsuka et al., 2020; Watari et al., 2012; Wiltschko et al., 2021). We previously identified a novel *Cry* gene in a pufferfish *Takifugu rubripes* and named it *TrCry6* (Okano et al., 2017). *Cry6* paralogs had been found in lower invertebrates (Oliveri et al., 2014), but there is no information on the molecular structure or function of CRY6 proteins.

*Xenopus tropicalis* is a model organism among amphibians that has the advantage of being diploid and having a relatively small genome size (Kashiwagi et al., 2010). We have previously searched for the *Cry* genes in *X. tropicalis* and reported mammalian‐type XtCRY1 and XtCRY2. Both the XtCRY1 and XtCRY2 strongly repressed the circadian clock transactivation complex CLOCK‐BMAL (Kubo et al., 2010), showing the circadian function of XtCRY1 and XtCRY2. Interestingly, *XtCry1/Cry2* showed very high and constant mRNA expression levels in the gonads, while they were widely transcribed in *X. tropicalis* tissues with daily variations (Kubo et al., 2010). Like *XtCry1/Cry2*, *XtCry4* also showed high mRNA expression in the gonads, but did not inhibit transcriptional activation by CLOCK‐BMAL (Takeuchi et al., 2014). These results implied that XtCRYs may have an unknown but common function in the gonads and their unique functions (circadian clock or magnetoreception) in the other tissues.

Ubiquitination is a well‐characterized post‐translational modification, in which carboxyl‐terminal glycine of ubiquitin is covalently bound to ε‐amino group of Lys residue of a target protein. In polyubiquitination, ubiquitin is linked to one of the seven Lys residues or the first Met of the previous ubiquitin to form complex polyubiquitin chains, while a single ubiquitin is linked to a target protein in monoubiquitination (Swatek & Komander, 2016). The structure of the ubiquitination defines the fate of the target protein, e.g., polyubiquitination on Lys^48^ and Lys^29^ is related to proteasome‐dependent protein degradation, while the other polyubiquitination and monoubiquitination is related to the other cellular processes such as endocytic traffic, mitophagy, and DNA‐damage response (Swatek & Komander, 2016).

Various ubiquitin‐binding domains have been identified, among which UIM (ubiquitin‐interacting motif) is an approximately 20‐amino‐acid stretch that forms an α‐helix (Harper & Schulman, 2006). UIM interacts with the ubiquitin such that the ubiquitin C‐terminus is directed toward the N‐terminus of UIM and is further classified into two classes: a single‐sided or overlapping double‐sided UIM (DUIM), either of which binds one or two ubiquitin molecule(s), respectively. DUIM has internally repeated UIM and is found in factors involved in receptor endocytosis such as Hrs and Eps15 (Dikic et al., 2009; Harper & Schulman, 2006; Hurley et al., 2006). Besides UIM, inverted ubiquitin‐interacting motif (IUIM, also termed MIU [motif interacting with ubiquitin]) has been identified to interact with ubiquitin in an inverted direction such that the ubiquitin C‐terminus is directed toward the C‐terminus of UIM (Penengo et al., 2006).

In the present study, we identified and characterized *Cry6* in *X. tropicalis* (*XtCry6*). We report expression of *XtCry6* mRNA in the native tissues and subcellular localization of XtCRY6 in cultured cells. In XtCRY6, we also found a new motif overlapping three ubiquitin‐interacting motifs of DUIM and IUIM, which is termed here “double‐sided and inverted ubiquitin‐interacting motif (DI‐UIM).” Interactions between DI‐UIM and ubiquitin molecules were assessed by GST pull‐down assay and in silico molecular analyses.

## 2 MATERIALS AND METHODS

### 2.1 Ethics statement

All experiments were conducted in accordance with the guidelines and regulations of Waseda University. All protocols were approved by the Committee for the Management of Biological Experiment at Waseda University (WD18‐047, WD21‐035, WD22‐045), and experimental animal care was conducted with permission from the Animal Experiment Committee of Waseda University (2018‐A091).

### 2.2 Animals

*X. tropicalis* were provided by the Institute for Amphibian Biology (Hiroshima University) through the National Bio‐Resource Project of the Ministry of Education, Culture, Sports, Science and Technology, Japan. *X. tropicalis* adults were entrained in light/dark cycles (LD, 12 h light: 12 h dark; 5–20 μW cm^−2^, fluorescent light, FHF32EX‐N‐HX‐S, NEC) for at least 1 week. They were fed commercial pellets once a day at a random timepoint. The temperature of the circulating water was kept at 27–28°C.

### 2.3 Cloning of cDNA of XtCry6

Total RNA was extracted from *X. tropicalis* larvae using TRIzol reagent (Invitrogen), and first‐strand cDNAs were synthesized with SuperScript III reverse transcriptase (Invitrogen) using oligo(dT)_21_ primer. Primers for cDNA cloning were designed on the basis of database‐deposited partial sequences, which anneal to the untranslated region of *XtCry6* gene (5′‐CGTCCAGCTGGGGCTTGTGTAAC‐3′ and 5′‐ATAAATATCTGTTTAGTTGACATAATAAAAATGGGACATAC‐3′). PCR products amplified with KODplus (TOYOBO) were inserted into the pMD20‐T vector (TaKaRa).

### 2.4 Molecular phylogenetic analysis

Protein sequences of CRY/photolyase family members were aligned by using MAFFT ver.7 (https://mafft.cbrc.jp/alignment/server/) using an advanced setting of G‐INS‐1 progressive method. The obtained alignment was further trimmed by deleting amino‐ and carboxyl‐terminal regions, aligned using MAFFT again, and analyzed by MEGA (ver.11.0.11) to obtain a neighbor‐joining (NJ) phylogenetic tree. Bootstrap probabilities were estimated from 1,000 replicates.

### 2.5 Entrainment and sampling

*X. tropicalis* were placed in an incubator (27°C) 1 week before sampling. They were entrained to an LD cycle (12 h light: 12 h dark; Figure S1, 100 ± 20 μW cm^−2^). Feeding was done only once on the fourth day to avoid food entrainment. At Zeitgeber time (ZT) 0‐ZT1 or ZT11‐ZT12 *X. tropicalis* were chilled on ice before sampling to avoid possible RNA synthesis or degradation. Tissues were collected under fluorescent light (240–300 μW cm^−2^). The liver, kidney, testis, and ovary were separated by 50 mg wet weight each, and eyeballs and brain were used as whole tissues. The tissues were collected and immediately homogenized in TRIzol reagent (Invitrogen).

### 2.6 RNA extraction and cDNA synthesis

Total RNA was extracted using TRIzol reagent (Invitrogen) following the manufacturer's instructions, then treated with RNase‐free Recombinant DNase I (Takara). We synthesized cDNA from 0.4 μg (eyeball) or 1 μg (brain, liver, kidney, testis, and ovary) of total RNA using High Capacity cDNA Reverse Transcription Kit (Thermo Fisher Scientific).

### 2.7 Primers

Several primer sets for amplification of *XtCry6* cDNA (Table 1) were designed on the basis of the genome databases using Primer3 (ver. 0.4.0, http://bioinfo.ut.ee/primer3-0.4.0/). They were designed so that no pair of the primers existed in a single exon. Serially diluted cDNA of all six tissues was subjected to quantitative RT‐PCR for selecting primers with amplification efficiency close to 100%. Finally, two independent pairs of primers (primer sets A and B, Table 1) were selected on the basis of the amplification curve, shape of the melting curve, and amplification efficiency. Primers for control genes (*XtHprt1* and *XtGusb*) were the same as those used in our previous studies (Kubo et al., 2010; Takeuchi et al., 2014).

**TABLE 1:** Primers used in quantitative RT‐PCR analysis

Gene	Xenbase gene ID	GenBank accession	Set	Sequence (5′ to 3′)	Amplification start	Amplification length	Reference
*XtCry6*	XB‐GENE‐5887601	XM_002938141	A	CGCAC CTTCT CAACT TCG	2,154	150	This study
				GACTG TTCAC GCCTT TCTTC TA			
			B	ACAGA AGGGG TTGGAC TCAG	1,246	82	This study
				CGATT GGTGC AGAGT TGTTC			
*XtHprt1*	XB‐GENE‐1014679	NM_203981		AGGCT CAGAC ATGGC GAG	23	119	Kubo et al. (2010)
				GTGGA ATGTA GACTT TCTCC AGATC			
*XtGusb*	XB‐GENE‐921780	XM_002935803		CATGG TGTCA ACAAA CATGA GGAC	1171	104	Kubo et al. (2010)
				GAGTT AGCAC CAAGC CACTT C			

### 2.8 Quantitative RT‐PCR

The relative mRNA expression levels of *XtCry6* were calculated using the ΔΔCt method as described previously (Okano et al., 2020). The reference gene was virtually defined as the average of threshold cycles (Ct) for *XtHprt1* (XB‐GENE‐1014679) and *XtGusb* (XB‐GENE‐921780), widely expressed house‐keeping genes with relatively stable expression levels across tissues. All the PCR products were subjected to 3% agarose gel electrophoresis to verify the appropriate amplification.

### 2.9 Cellular localization of CRY6 in HEK293 cells

pcDNA‐GFP‐XtCRY6 for expression of GFP‐tagged full‐length XtCRY6 was constructed by transferring *XtCry6* cDNA into pcDNA‐DEST53 vector (Invitrogen) by PCR and SLiCE reaction (Motohashi, 2015). pcDNA‐GFP‐XtCRY6NLS/NoLS was constructed by inserting annealed synthetic oligonucleotide into NotI site of pcDNA‐DEST53 vector to encode three tandem repeats of the putative NLS/NoLS of XtCRY6 (AAA‐[RRSRKKKKSA]_3_[stop]). A control plasmid pcDNA‐GFP‐SV40NLS was similarly constructed to encode tandem repeats of SV40NLS (AAA‐[DPKKKRKV]_3_‐DTAA[stop]). pcDNA‐GFP‐XtCRY1 (Kubo et al., 2010) was also used as a control. HEK293 cells (RIKEN Cell Bank, RCB1637) were plated onto slide glass (Matsunami, TF0215). Expression plasmids were transfected into HEK293 cells using Lipofectamine and Plus Reagent (Invitrogen) according to the manufacturer's instructions. Twenty‐four hours after transfection, the cells were fixed with ice‐cold methanol for 5 min, and then washed with PBS (10 mM NaH_2_PO_4_, 140 mM NaCl, 1 mM MgCl_2_, pH 7.4). The cells were incubated Hoechst 33342 solution (DOJINDO) for 5 min before microscopic observation.

### 2.10 GST pull‐down assay

To obtain GST‐XtCRY6DI‐UIM_WT (wild‐type DI‐UIM), GST‐XtCRY6DI‐UIM_SFL (shuffled DI‐UIM), and GST‐HsRabex5IUIM (a positive control IUIM) fusion proteins, three kinds of expression plasmid were constructed by inserting annealed synthetic oligonucleotides into BamHI site of pGEX5X‐1 vector to encode IGGGGSGGGG SADLETLGYE TDLELAIALS LQEHNQLTDE FTKTKAARMT EQT[stop] (XtCRY6DI‐UIM_WT, XtCRY6‐derived sequence is underlined) or IGGGGSGGGG SADLETL LEY GADLITAQES ELL HNQLTDE FTKTKAARMT EQT[stop] (XtCRY6DI‐UIM_SFL, shuffled DI‐UIM is double‐underlined) or IGGGGSGGGG SQKQIQEDWE LAERLQREEE EAFASSQSEF TKTKAARMTE QT[stop] (HsRabex5IUIM, HsRabex5‐derived sequence is underlined), respectively. Each GST fusion protein was purified from *Escherichia coli* BL21 that was transformed with the expression plasmid. The purified GST‐fusion protein (3 μg) was mixed with polyubiquitin (2.2 μg, K48Ub_2–8_, UBPBio, D1500) in binding buffer (25 mM HEPES[pH 7.2], 125 mM CH_3_COOK, 2.5 mM CH_3_COOMg, 5 mM EDTA, 0.5% TritonX‐100, 1 mM dithiothreitol, 1× cOmplete protease inhibitor cocktail (Rosche, 4693116001) and incubated with rotation for 30 min at 4°C. Then, the sample was mixed with glutathione‐conjugated magnetic beads (MagneGST Glutathione Particles, Promega, V8611) and incubated with rotation for 1 h at 4°C. After unbound materials were washed out with the binding buffer, the precipitants were eluted with 50 mM glutathione (reduced form) in the binding buffer (pH 7.69) and analyzed by immunoblot analysis (Watari et al., 2012) using anti‐ubiquitin polyclonal antibody (Proteintech, 102021‐2‐AP) and anti‐GST monoclonal antibody (B‐14, Santa Cruz Biotechnology, sc‐138). In competition experiments, 2.2 μg of the polyubiquitin was mixed with 1 μg of synthetic polypeptide (XtCRY6DI‐UIM_WT, ADLETLGYETDLELAIALSLQEHNQLTD or XtCRY6DI‐UIM_SFL, ADLETLLEYGADLITAQESELLHNQLTD, Genscript) and incubated for 1 h at 4°C prior to the incubation with the GST‐fusion protein.

### 2.11 Molecular modeling

Structure predictions were performed using ColabFold: AlphaFold2 using MMseqs2 (https://colab.research.google.com/github/sokrypton/ColabFold/blob/main/AlphaFold2.ipynb#scrollTo=kOblAo-xetgx) or AlphaFold Colab (https://colab.research.google.com/github/deepmind/alphafold/blob/main/notebooks/AlphaFold.ipynb). XtCRY6 was divided into four regions based on the predicted aligned error (pAE) and pLDDT, among which two regions with low pAE scores and high pLDDT scores were subjected to further structure prediction using ColabFold. PyMOL 2.5.0 (Open‐Source PyMol) was used for rendering of the molecular structures.

## 3 RESULTS

**FIGURE 1:**
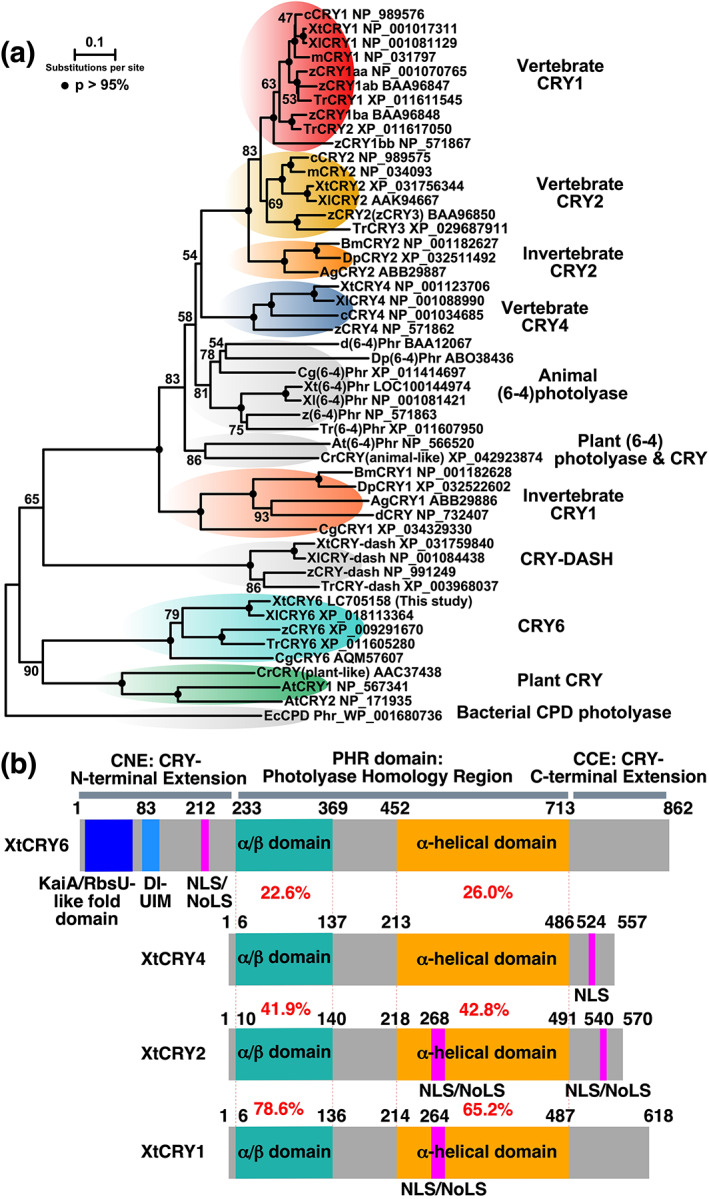
Phylogenetic analysis and molecular architectures of CRYs in *X. tropicalis*. (a) A molecular phylogenetic tree (NJ tree) of CRYs and photolyases. Values at the branches indicate bootstrap probabilities. Each protein is shown with combining the abbreviated species name (m, mouse; c, chicken; z, zebrafish; d, *Drosophila* [fruit fly]) or scientific name (Ag, *Anopheles gambiae*; At, *Arabidopsis thaliana*; Bm, *Bombyx mori*; Cg, *Crassostrea gigas* [oyster]; Cr, *Chlamydomonas reinhardtii*; Dp, *Danaus plexippus plexippus*; Ec, *Escherichia coli*; Tr, *Takifugu rubripes* [pufferfish]; Xt, *X. tropicalis*; Xl, *X. laevis*) and protein name followed by its accession ID. Alignment of N‐ and C‐terminally deleted sequences (corresponding to Asp^244^‐Tyr^691^ of XtCRY6) are shown in Figure S2. (b) Domain structures of *X. tropicalis* CRYs. Amino acid positions shown are start of domains/motifs and start/end of the protein. DI‐UIM, double‐sided/inverted ubiquitin‐interacting motif; NLS, nuclear localization signal; NoLS, nucleolar localization signal

We previously identified a pufferfish (*Takifugu rubripes*) gene closely related to plant *Cry*s, and named it *TrCry6* (Okano et al., 2017). We found its orthologs in the databases of the *X. tropicalis* (XtCRY6, GenBank acc. No. LC705158) and *X. laevis* (XlCRY6)(Figures 1, S2, and S3). A phylogenetic tree was constructed on the basis of protein sequence similarities in the PHR domain (Figures 1 and S2), and it suggested that CRY6 is relatively distant from the other vertebrate CRYs (CRY1/2/4) and instead close to plant CRY. We also found that approximately 200‐amino‐acid‐long N‐terminal‐extended sequence (termed CNE as CRY N‐terminal extension) exist in the primary structures of XtCRY6 in addition to PHR domain (PHR, photolyase homology region including *Cry* α/β domain and α helical domain), and CCE (CRY C‐terminal extension) that are commonly found in all CRY family proteins (Figure 1b). Noticeably, the CNE was found in predicted CRY6 proteins in the other species (Figure S3).

**FIGURE 2:**
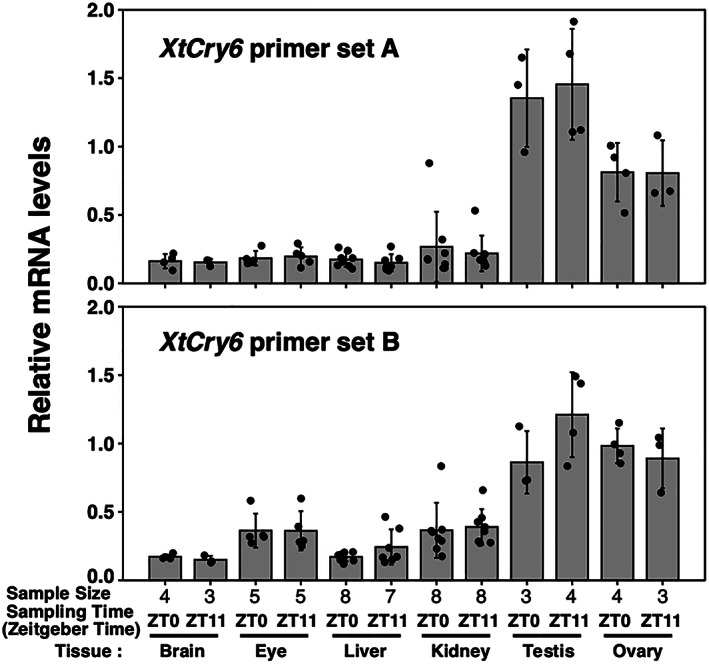
Quantitative RT‐PCR analysis of tissue and temporal expression of *Cry6* in *Xenopus tropicalis*. Each tissue was collected at ZT0‐ZT1 and ZT11‐ZT12. *XtCry6* mRNA level was calculated as a value relative to that of the *XtHprt1* and *XtGusb* gene. Error bars represent ± SD. *XtCry6* primer pair set A (above); *XtCry6* primer pair set B (below). Primer sequences are presented in Table 1

Firstly, we examined *XtCry6* mRNA expression in various adult tissues to infer the molecular function. To date, the mRNA expression pattern of vertebrate *Cry6* has been examined only in fish cultured cells (Okano et al., 2017; Oliveri et al., 2014), and so we examined the expression level of *XtCry6* mRNA in each tissue of *X. tropicalis*, using qRT‐PCR. We designed two sets of primers (Table 1) to amplify different stretches of *XtCry6* cDNA for precise measurements. We compared relative *XtCry6* mRNA levels in the eye, brain, liver, kidney, testis, and ovary, and both primers gave similar results showing relatively higher expressions in the testis and ovary than the other tissues (Figure 2). Considering the possibility of diurnal variation, we examined two points during the day but found no significant difference between ZT0 and ZT11 in each tissue (Figure 2).

**FIGURE 3:**
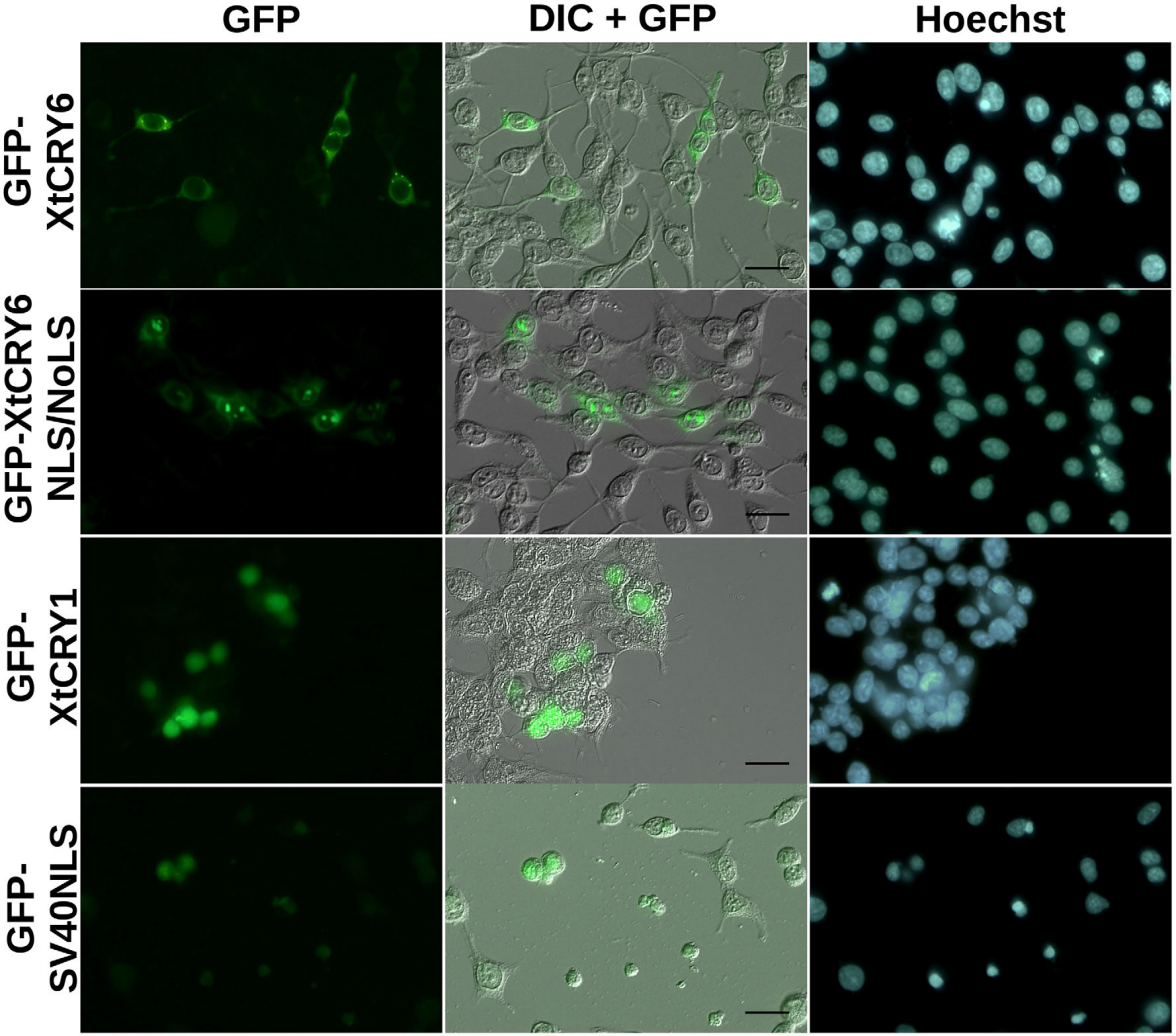
Cellular localization of GFP‐fused XtCRY6 and XtCRY6NLS/NoLS expressed in HEK 293 cells. Expression vector (pcDNA‐GFP‐XtCRY6 or pcDNA‐GFP‐XtCRY6NLS/NoLS or pcDNA‐GFP‐XtCRY1 or pcDNA‐GFP‐SV40NLS) was transfected into HEK 293 cells. After 24 h, the cells were treated Hoechst 33342 (Dojindo), and observed using a fluorescence microscope for detection of GFP (left, GFP) and nuclei (right, Hoechst) or a differential interference microscope (middle, DIC, GFP signals are overlaid). Scale bars, 20 μm

In the CRY6 CNE, we found a putative nuclear localization signal (NLS) (Kosugi et al., 2009) (Figure 1b). The consensus NLS in CNE of XtCRY6 (Arg^212^‐Ala^221^ [RRSRKKKKSA] predicted by cNLS mapper (Kosugi et al., 2009); Arg^212^‐Lys^218^ [RRSRKKK] predicted by Predict NLS [https://rostlab.org/owiki/index.php/PredictNLS]) further contained putative nucleolar localization signals (NoLS)(RRSR, RKKK and KKKK, consensus [R/K][R/K]X[R/K], Endo et al., 2009). So, we examined the activity of the NLS/NoLS peptide [RRSRKKKKSA]_3_ by investigating the subcellular localization of GFP‐tagged XtCRY6 and GFP‐tagged XtCRY6NLS/NoLS peptide in cultured mammalian cells (Figure 3). GFP‐XtCRY6 fluorescence was detected mostly in the cytoplasm, not in the nucleus (Figure 3, upper left panel). On the other hand, strong fluorescent signals were detected in GFP‐XtCRY6NLS/NoLS‐expressing cells in addition to weak cytoplasmic signals (Figure 3, GFP‐XtCRY6NLS/NoLS). Overlaying the fluorescent signals with DIC image strongly suggested localization of those signals in the nucleolus (Figure 3). This is in clear contrast with GFP‐XtCRY1 and GFP‐SV40NLS, both of which are most exclusively detected in the nucleus.

**FIGURE 4:**
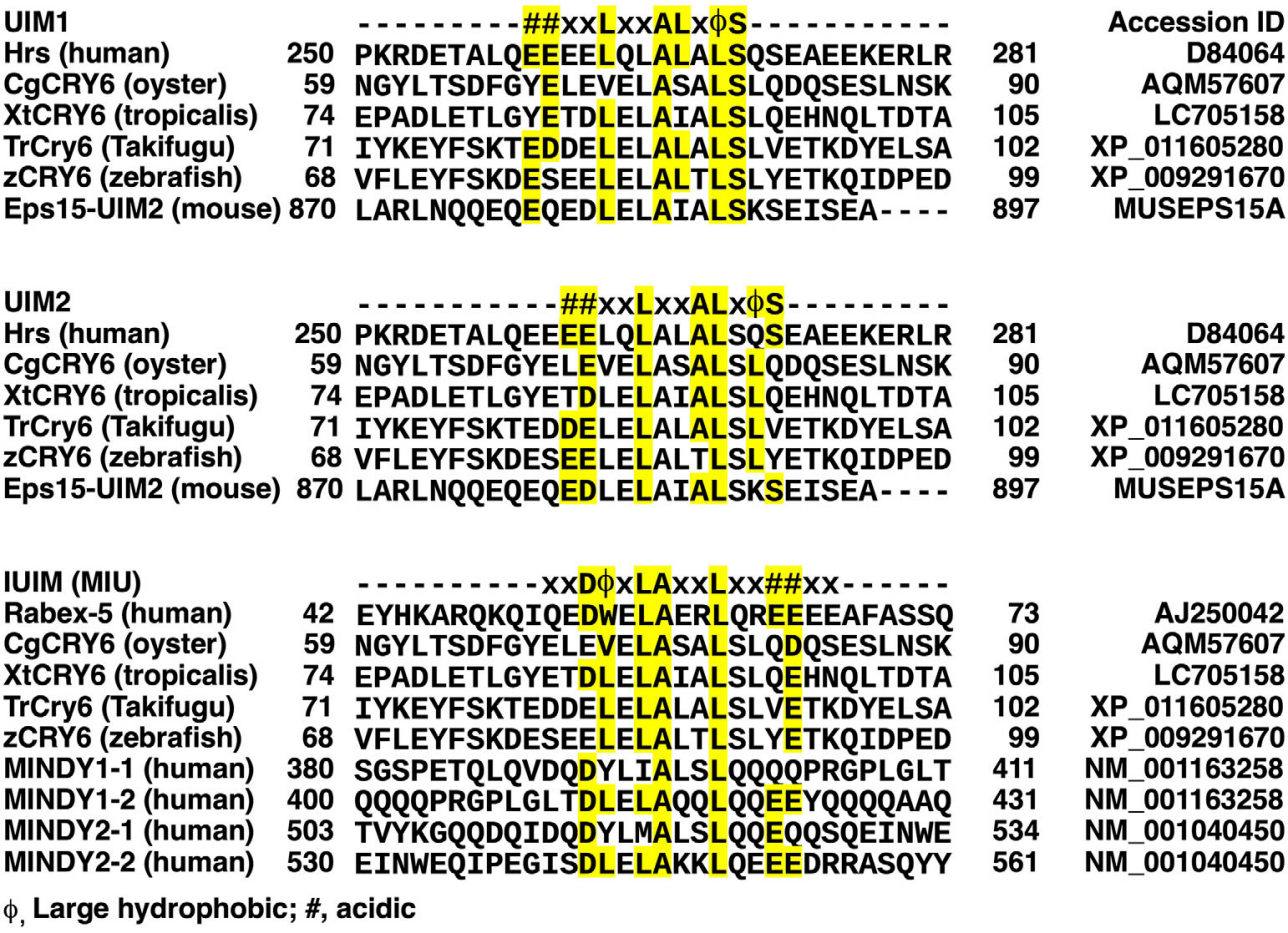
Comparison of DUIM and IUIM in various ubiquitin‐interacting proteins and CRY6 proteins. Amino acid sequences of putative DI‐UIM of CRY6s were compared with DUIM (upper and middle alignments, UIM1 and UIM2, respectively) and IUIM (lower alignments). Consensus motifs (Penengo et al., 2006) are shown in the top of alignments, and conserved residues are colored in yellow

We also found ubiquitin‐interacting motifs in XtCRY6CNE, which were conserved in the CNE of Takifugu and zebrafish CRY6 (UIM1, UIM2, and IUIM in Figures 4). The UIM1 and UIM2 likely constitute a motif of double‐sided ubiquitin‐interacting motif (DUIM). The putative DUIM of XtCRY6 was highly similar to those of Hrs (hepatocyte growth factor‐regulated tyrosine kinase substrate) and Eps15 (EGFR pathway substrate 15), endocytic adaptor proteins (Dikic et al., 2009; Harper & Schulman, 2006; Hurley et al., 2006). Notably, the DUIM of XtCRY6 is also similar to the consensus of IUIM (Penengo et al., 2006) (Figure 4). We term the overlapping DUIM and IUIM as DI‐UIM.

**FIGURE 5:**
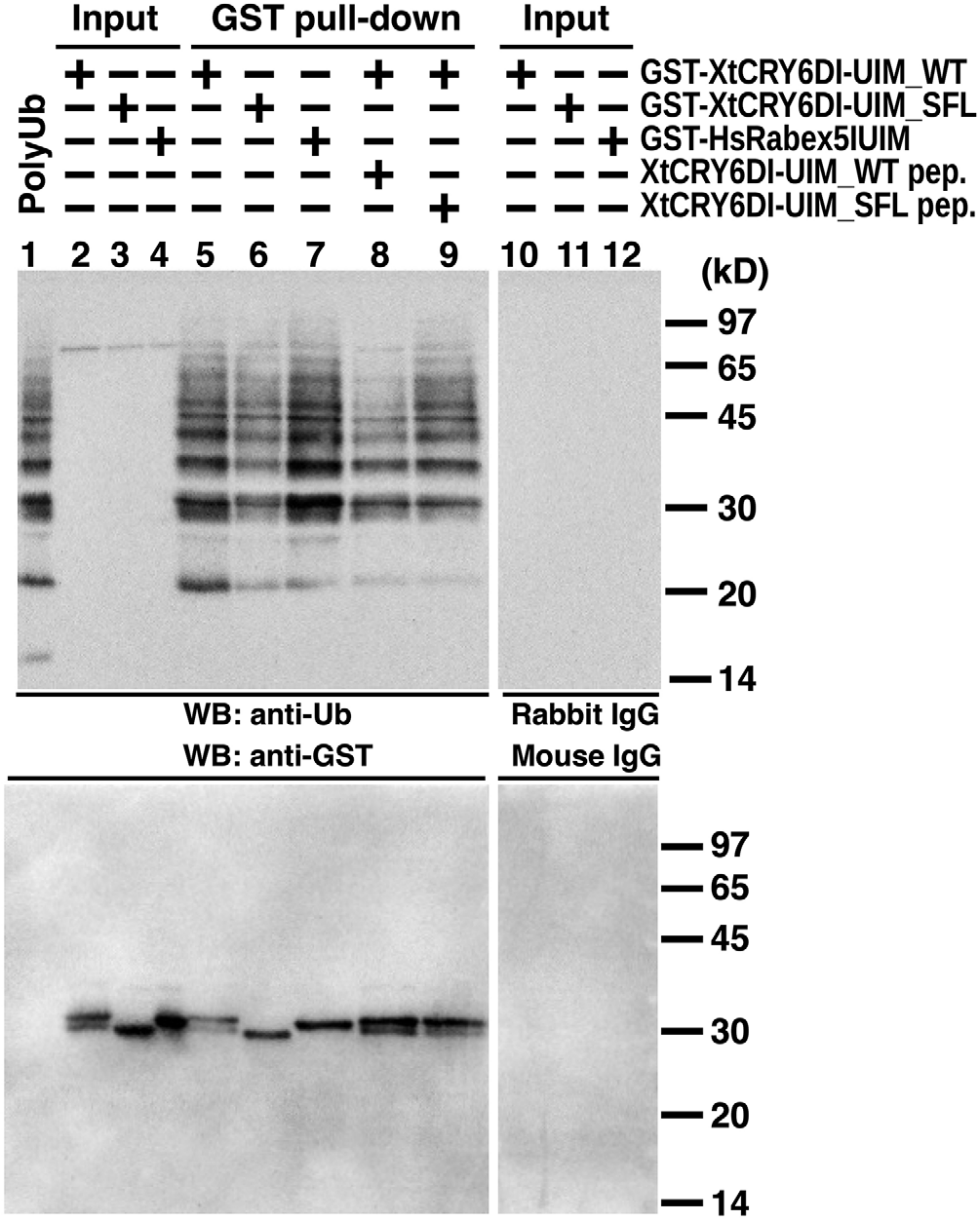
GST pull‐down assay. GST‐fusion protein (GST‐XtCRY6DI‐UIM_WT, GST‐XtCRY6DI‐UIM_SFL, GST‐Rabex5_IUIM) was subjected to in vitro pull‐down assay using K48Ub_2–8_ (GST pull‐down; lanes 5–9), and the precipitant corresponding to 1.8 μg of input GST‐fusion protein was loaded in each lane. Loaded as an input was 1.8 μg of each GST‐fusion protein (Input; lanes 2–4, and 10–12). Antibodies used are anti‐ubiquitin antibody (upper panel) and anti‐GST antibody (lower panel)

Then, we performed GST pull‐down assay to assess activity of XtCRY6 DI‐UIM. We compared polyubiquitin‐binding activities of two GST fusion proteins, GST‐XtCRY6DI‐UIM_WT and GST‐XtCRY6DI‐UIM_SFL, which contain a wild‐type XtCRY6DI‐UIM and its shuffled polypeptide, respectively (Figure 5). GST‐XtCRY6DI‐UIM_WT precipitated polyubiquitin (Figure 5, lane 5) similar to a positive control GST‐HsRabex5_IUIM (lane 7), while GST‐XtCRY6DI‐UIM_SFL precipitated less amount of polyubiquitin (lane 6). The polyubiquitin binding to GST‐XtCRY6DI‐UIM_WT was more strongly suppressed by preincubation of polyubiquitin with XtCRY6DI‐UIM_WT peptide (Figure 5, lane 8) than that with XtCRY6DI‐UIM_SFL (lane 9), confirming direct binding of polyubiquitin to XtCRY6 DI‐UIM. Analysis using anti‐GST antibody (Figure 5, lower panel) showed sufficient precipitations of GST‐fusion proteins. GST‐XtCRY6DI‐UIM_SFL (lane 6) showed a slightly higher electromobility than GST‐XtCRY6DI‐UIM_WT (lane 5). This difference is likely due to not degradation of GST‐XtCRY6DI‐UIM_SFL but structural difference between the two polypeptides, because these bands were detected by using the THETAS tag (Miura et al., 2019) fused at the carboxyl terminal (TKAARMTEQT) (not shown).

**FIGURE 6:**
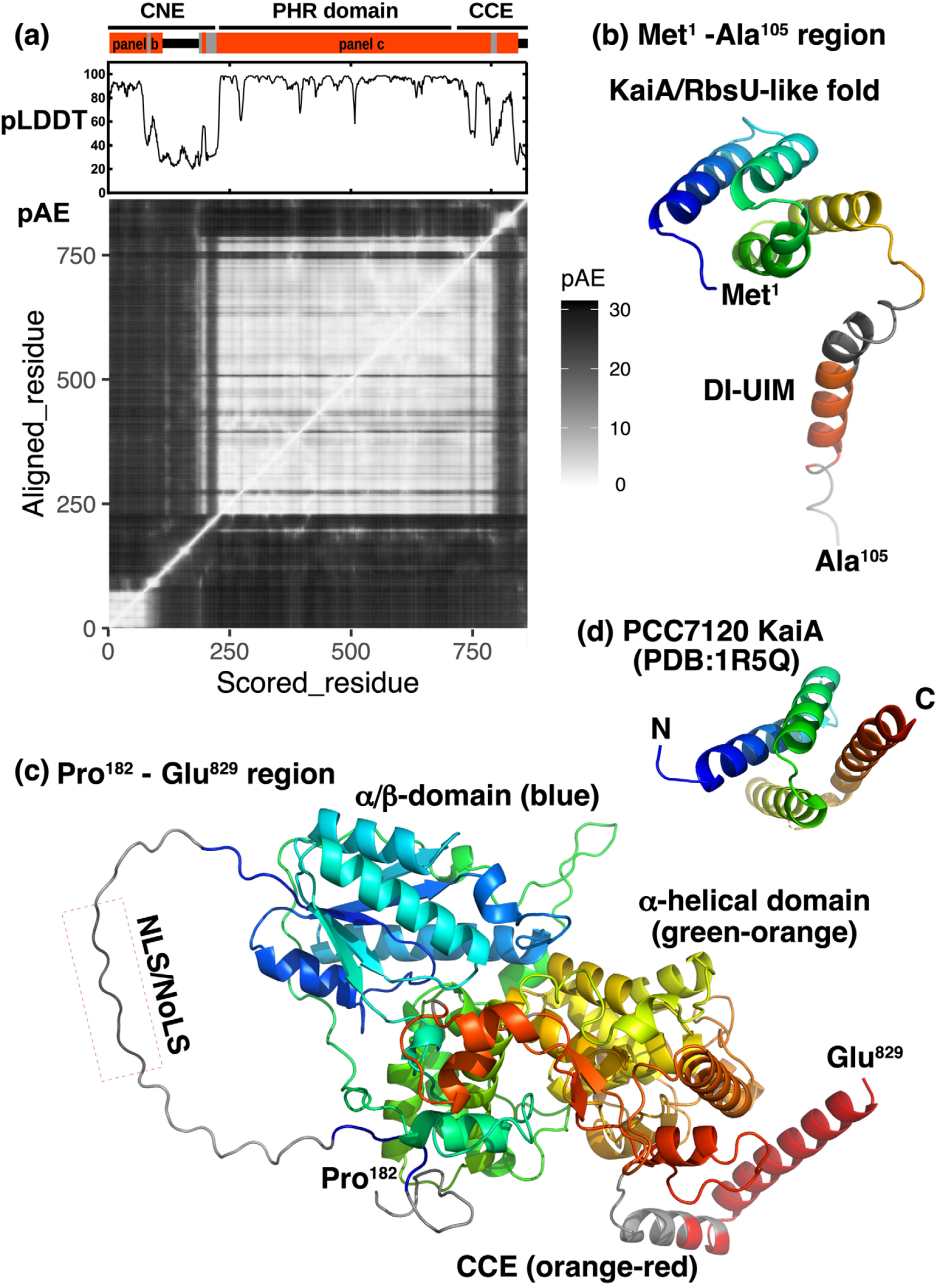
Predicted molecular structures of XtCRY6. (a) Plots of pLDDT (upper) and pAE (lower) scores from structural prediction of full‐length XtCRY6. Red bars above the pLDDT plot indicate two regions subjected to the molecular modeling shown in panels b and c. (b) A predicted structure of N‐terminal region of XtCRY6 (a part of the CNE; Met^1^‐Ala^105^). (c) A predicted structure of PHR domain and a part of CCE region of XtCRY6 (Pro^182^‐Gle^829^). (d) 3D structure of KaiA from PCC7120 based on a crystal structure analysis (PDB accession code 1R5Q)

Next, structures of XtCRY6 were predicted by using ColabFold (Figure 6). A preliminary prediction of the full‐length XtCRY6 structure resulted in low pLDDT score and high predicted aligned error scores (pAE) in some regions (Figure 6a). Because such regions likely correspond to flexible or linker regions with low structural reliabilities, we excluded Ser^106^‐Glu^181^ and Gly^830^‐Asn^862^ (shown by black bars in upper part of Figure 6a) from further predictions due to their low pLDDT and high pAE scores. Then, Met^1^‐Ala^105^ and Pro^182^‐Glu^829^ were separately subjected to ColabFold (Figure 6b, c, respectively), although they still contained short regions with relatively low pLDDT (pLDDT <50; colored in gray in Figure 6a–c). The predicted structure of Met^1^‐Ala^105^ (Figure 6b) contained a tetrahelical fold domain at proximity of the amino‐terminus (KaiA/RbsU‐like fold). This domain formed a right‐handed superhelix that is topologically related to KaiA (KaiA/RbsU domain, SCOP ID 2000595; Figure 6d), a core circadian clock component in cyanobacteria *Anabaena* sp PCC7120 (Garces et al., 2004), whereas there is no apparent sequence homology between XtCRY6 and KaiA/RbsU. The predicted structure of Met^1^‐Ala^105^ region also contained an α‐helix corresponding to the DI‐UIM. The pLDDT score of KaiA/RbsU‐like fold and DI‐UIM were relatively high (>50) in contrast to their adjacent regions (<50, colored in gray in Figure 6b), implying relatively high stability and probability of KaiA/RbsU‐like fold and DI‐UIM structures. The Pro^182^‐Glu^829^ region is composed of a part of CNE, PHR domain, and a part of CCE (Figure 6a, c). Some regions in the CNE including NLS/NoLS‐like motifs (colored in gray in Figure 6c) had relatively low (<50) pLDDT scores, possibly due to their high flexibilities.

**FIGURE 7:**
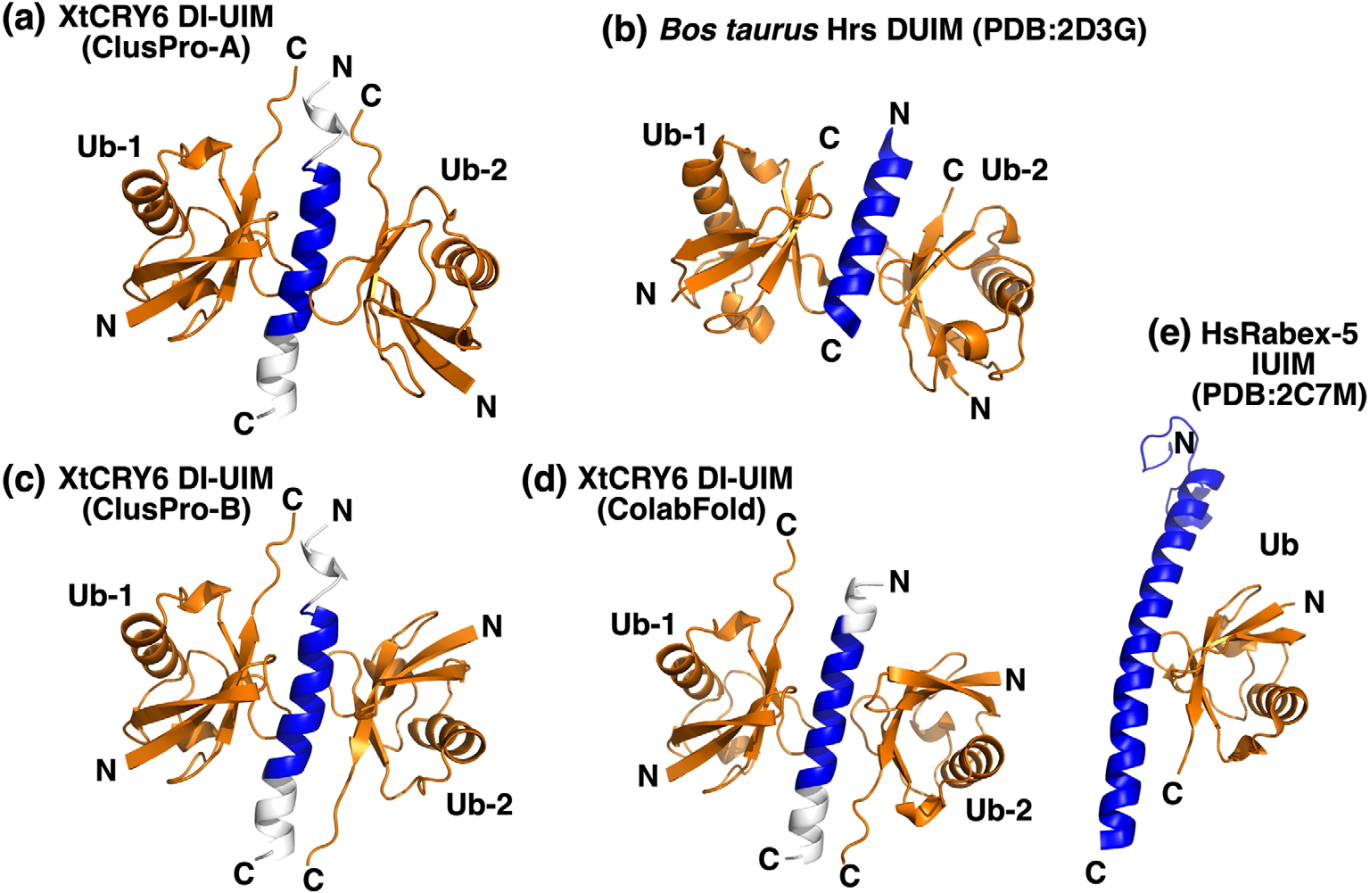
Predicted structures of XtCRY6 DI‐UIM binding two ubiquitin molecules. (a) A predicted structure (ClusPro‐A model) of XtCRY6 DI‐UIM binding two ubiquitin molecules being related by twofold rotational symmetry with the symmetry axis in parallel to the DI‐UIM helix. (b) A structure of *Bos taurus* Hrs DUIM binding two ubiquitin molecules (Hirano et al., 2006) being related by twofold rotational symmetry with the symmetry axis in parallel to the DUIM helix (PDB accession code 2D3G). (c) A predicted structure (ClusPro‐B model) of XtCRY6 DI‐UIM with two ubiquitin molecules that are related by twofold rotational symmetry with the symmetry axis being perpendicular to the DI‐UIM helix. (d) A ColabFold‐predicted structure of XtCRY6 DI‐UIM with two ubiquitin molecules interacting in inverse directions along the helix. (e) 3D structure of human Rabex‐5 based on a crystal structure analysis (PDB accession code 2C7M)

Finally, we performed docking simulations to infer the ubiquitin‐binding activity of each binding site in the DUIM and IUIM by using ClusPro and ColabFold (Figure 7). Amino acid sequences of XtCRY6 DI‐UIM with the flanking amino acids (L^78^ETLGYETDLELAIALSLQEHNQLTDTA^105^) and a monomeric form of *X. tropicalis* ubiquitin (XtUbiquitin, retrieved from *X.tropicalis* polyubiquitin‐C [https://www.kegg.jp/entry/xtr:448120]) were predicted by ColabFold and AlphaFold Colab, respectively. Then, those structures were subjected to docking simulation by using ClusPro 2.0 (https://cluspro.org/). ClusPro predicted 10 complex structures composed of XtCRY6DI‐UIM and one ubiquitin molecule, among which one showed the largest interaction energy was selected. Then, the XtCRY6DI‐UIM:ubiquitin complex was further subjected to the second docking simulation with another ubiquitin molecule. Two out of 15 predicted complex structures (Figure 7a, c) were selected by the criterion of interaction of the second ubiquitin to the DI‐UIM. In a model shown in Figure 7a (ClusPro‐A model), two ubiquitin molecules bind UIM1 and UIM2 in a relationship of twofold rotational symmetry with the symmetry axis in parallel to the UIM helix like a Hrs:ubiquitin complex (PDB accession code 2D3G, Figure 7b). On the other hand, in a model shown in Figure 7c (ClusPro‐B model), the second ubiquitin molecule (Ub‐2) interacts with IUIM like a Rabex‐5:ubiquitin complex (Figure 7e), and hence the two ubiquitin molecules bind to UIM1 and IUIM in a relationship of twofold rotational symmetry with the symmetry axis being perpendicular to the DI‐UIM helix.

Without using ClusPro, Colabfold predicted a complex structure of DI‐UIM and two ubiquitin molecules (Figure 7d) that was very similar to the ClusPro‐B model (Figure 7c).

## 4 DISCUSSION

In this study, we identified XtCRY6 forming an independent group within the CRY/photolyase family with a unique CNE region and revealed its unique structures, tissue expression and subcellular localization. Although its physiological function is not fully understood, we partially characterized potential functions of the putative NoLS and DI‐UIM in the CNE.

Invertebrate CRY1, such as *Drosophila* CRY (dCRY), binds FAD and functions as a photoreceptor (circadian photoreceptor) for synchronization of the circadian clock (Foley & Emery, 2020). On the other hand, mammalian CRYs are likely not photosensitive. Mammalian‐type CRYs in *X. tropicalis* (XtCRY1 and XtCRY2) have one or two NLS (Figure 1b) and are accumulated in the nucleus (Figure 3 and Kubo et al., 2010). XtCRY1 and XtCRY2 strongly repressed BMAL/CLOCK‐mediated transcriptional activation (Kubo et al., 2010). Therefore, both XtCRY1 and XtCRY2 may be the circadian core oscillatory transcriptional repressors in the central mechanism of circadian clock oscillation like in mammals (Kubo et al., 2010). On the other hand, XtCRY4 is a cytosolic protein showing no transcriptional repressor activity (Takeuchi et al., 2014) and may function as a magnetoreceptor considering that avian CRY4 is a strong candidate for the light‐driven magnetoreceptor (Günther et al., 2018; Mitsui et al., 2015; Otsuka et al., 2020; Watari et al., 2012; Wiltschko et al., 2021). Because it is not clear at present whether XtCRY6 is expressed in the nucleus or not, it is interesting to investigate its transcriptional regulation function.

To infer the molecular function of XtCRY6, a classical but still important approach is to measure its tissue and temporal expression in animals. So, we firstly investigated *Cry6* mRNA expression, which was relatively higher in testis and ovary than in other tissues (Figure 2). This result implies that XtCRY6 may be involved in functions specific to the testis and ovary. It should be noted that *XtCry1/2/4* mRNAs are also highly expressed in testis and ovary (Kubo et al., 2010; Takeuchi et al., 2014), and therefore it is possible that CRY serves unique functions in the gonads and common function in the other tissues.

*Cry6* gene is found only in the genomes of invertebrates and metamorphic vertebrates below amphibians (Figure 1a). This together with the phylogenetic analysis suggest that *Cry6* may be an ortholog of plant *Cry* genes retained in animals for a long time but lost in the ancestral reptiles. CRY6, however, is substantially different from the other CRYs including plant CRY in that CRY6 has the CNE, indicative of emergence of *Cry6* gene by domain shuffling during animal evolution. CRY6 may also be photosensitive, because all groups of cryptochromes except for mammalian CRYs are thought to bind FAD chromophore. Taking into account the function of plant CRYs as blue‐light photoreceptor for photomorphogenesis, CRY6 might play a similar role or unique one depending on the CNE. Molecular characterization of both the CRY6 PHR domain and CNE would be an important clue to approach the photosensitivity and biological function of CRY6.

In the CNE, XtCRY6 has three important features, putative NLS/NoLS, KaiA/RbsU‐like fold domain, and DI‐UIM (Figures 1b and 4). The putative NLS/NoLS sequences elicited nucleolar localization in HEK293 cells (Figure 3, GFP‐XtCRY6NLS/NoLS), and similar sequences were highly conserved among the CNE of CRY6 in the other species (Figure S3). Thus it is possible that the NoLS of XtCRY6 is functional, although the full‐length XtCRY6 did not migrate to the nucleus or nucleolus (Figure 3). The NoLS of XtCRY6 may be not active in the full‐length protein under our experimental condition but activated under physiological condition. Alternatively, XtCRY6 is a cytoplasmic protein and the NLS/NoLS‐like motif plays the other function(s).

KaiA/RbsU‐like fold domain is composed of four helices in a right‐handed superhelix (Figure 6d). In *Synechococcus elongatus* PCC 7942, KaiA/RbsU domain has shown to be essential to dimerization (Hayashi et al., 2004), binding with KaiC (Iwasaki et al., 1999), and phosphoregulation of KaiC (Iwasaki et al., 2002). A similar bundle structure is also found in the N‐terminal RbsT‐interaction domain of RbsU, a bacterial phosphoserine phosphatase (Hardwick et al., 2007). Thus, the KaiA/RbsU‐like fold domain of CRY6 might be involved in similar mechanisms such as protein–protein interaction and/or phosphoregulation.

Regarding the function of DI‐UIM, we verified its potential activity by GST pull‐down assay (Figure 5) but used only K48Ub_2–8_. The DI‐UIM may be involved in a complex selectivity in the interaction with (poly)ubiquitinated protein, because DUIM and IUIM overlap each other in DI‐UIM to elicit a competition between two sterically different modes of interaction as shown in Figure 7. Comparative measurements of the binding affinity between each binding site and various structures of polyubiquitins together with the structural analysis of those complexes would reveal the competitive regulation on DI‐UIM. In such an analysis, many structural patterns are possible in the polyubiquitination, and so encompassing analysis is likely necessary.

The nucleolus is a nuclear subdomain specialized for rRNA transcription and processing, and these functions are regulated by ubiquitin dynamics, in which a nucleolar protein nucleophosmin/B23 (NPM) may play an important role (Itahana et al., 2003). NPM recruits a deubiquitinating enzyme USP36 into the nucleolus by binding NoLS of USP36 (Endo et al., 2009). Importantly, NMP itself is deubiquitinated by USP36 and stabilized by inhibiting its ubiquitination‐dependent degradation. XtCRY6 possibly localizes in the nucleolus and interacts with polyubiquitinated proteins such as NPM through NoLS and/or DI‐UIM, respectively (Figures 3 and 5), and if so, XtCRY6 would be involved in rRNA transcription and processing.

In this study, we were able to identify XtCRY6 and partially characterized its function. To elucidate physiological function of CRY6, future studies should be undertaken to assess the chromophore binding, photosensitivity, transcriptional repression ability, and effects of genetic ablation.

## AUTHOR CONTRIBUTIONS

All authors conducted the experiments and data analyses. K.O. and T.O. conceived and designed the experiments, and wrote the manuscript.

## FUNDING INFORMATION

This work was supported by the Japanese Society for the Promotion of Science (JSPS 18K19348) and by MEXT Quantum Leap Flagship Program (MEXT Q‐LEAP) Grant Number JPMXS0120330644, awarded to T.O.

## CONFLICT OF INTEREST STATEMENT

The authors declare that they have no competing interests.

## Supporting information


**Data S1:** Supplementary Information
