# Supplementary material for: Molecular functions of the double‐sided and inverted ubiquitin‐interacting motif found in Xenopus tropicalis cryptochrome 6
Source: Dev Growth Differ. 2023 May 16;65(4):203–14. doi: 10.1111/dgd.12852 (PMC11520951; doi:10.1111/dgd.12852)
Supplement: Supplementary file 1 — Data S1: Supplementary Information [file DGD-65-203-s001.docx]

Supplemental Materials for:

**Analysis of *Xenopus tropicalis* cryptochrome 6 revealed a conserved double-sided/inverted ubiquitin-interacting motif (DI-UIM) in the amino-terminal extension region*.***

Okano Keiko, Otsuka Hiroaki, Nakagawa Marika, and Okano Toshiyuki*

**Affiliation**

Department of Electrical Engineering and Bioscience, Graduate School of Sciences and Engineering, Waseda University, TWIns, Wakamatsucho 2-2, Shinjuku-Ku, Tokyo 162-8480, Japan.

***Corresponding Author:**

Dr. Toshiyuki Okano

Department of Electrical Engineering and Bioscience, Graduate School of Advanced Science and Engineering, Waseda University, Wakamatsu-cho 2-2, Shinjuku-ku, Tokyo 162-8480, Japan

tel and fax: +81-3-5369-7316

e-mail: [okano@waseda.jp](mailto:okano@waseda.jp)

**Figure S1 Emission spectra of LEDs.**LEDs used are OSTCXBCBC1E OptoSupply (Power LED, λmax = 460 nm, λmax = 525 nm, λmax = 624 nm,). The spectrum was measured using a photonic multichannel spectral analyzer (Hamamatsu Photonics, Model PMA-11; type C7473-36).

**(Figure S2: 1/8)**

**cCRY1_NP_989576 1:DNPALRECIR --------GA DT------VR CVYILDPWFA GSS------- --NVGINRWR FLLQCLEDLD 47**

**XtCRY1_NP_001017311 1:DNPALRECIQ --------GA DT------IR CVYILDPWFA GSS------- --NVGINRWR FLLQCLEDLD 47**

**XlCRY1_NP_001081129 1:DNPALRECIQ --------GA DT------VR CVYILDPWFA GSS------- --NVGINRWR FLLQCLEDLD 47**

**mCRY1_NP_031797 1:DNPALKECIQ --------GA DT------IR CVYILDPWFA GSS------- --NVGINRWR FLLQCLEDLD 47**

**zCRY1aa_NP_001070765 1:DNPSLRDSIL --------GA HS------VR CVYILDPWFA GSS------- --NVGISRWR FLLQCLEDLD 47**

**zCRY1ab_BAA96847 1:DNPSLRDSIK --------GA DN------LR CVYILDPWFA GSS------- --NVGISRWR FLLQCLEDLD 47**

**TrCRY1_XP_011611545 1:DNPSLKDSLL --------GA DS------VR CVYILDPWFA GSS------- --NVGINRWR FLLQSLEDLD 47**

**zCRY1ba_BAA96848 1:DNPALQEAVR --------GA DT------VR CVYFLDPWFA GSS------- --NLGVNRWR FLLQCLDDLD 47**

**TrCRY2_XP_011617050 1:DNPALREAVR --------GA GT------VR CVYFLDPWFA GSS------- --NVGVNRWR FLLQCLEDLD 47**

**zCRY1bb_NP_571867 1:DNPALLEALN --------GA DT------LR CVYFLDPWFA GAS------- --NLGVNRWR FLLQSLEDLD 47**

**cCRY2_NP_989575 1:DNPALQAALR --------GA AS------LR CIYILDPWFA ASS------- --AVGINRWR FLLQSLEDLD 47**

**mCRY2_NP_034093 1:DNPALLAAVR --------GA RC------VR CVYILDPWFA ASS------- --SVGINRWR FLLQSLEDLD 47**

**XtCRY2_XP_031756344 1:DNPALSAALR --------DA TS------VR CVYILDPWFA ASS------- --SGGVNRWR FLLQSLEDLD 47**

**XlCRY2_AAK94667 1:DNPALLAALR --------GA NS------VR CVYILDPWFA ASS------- --SGGVNRWR FLLQSLEDLD 47**

**zCRY2(zCRY3)_BAA96850 1:DNPALQEALN --------GA DT------VR CVYILDPWFA GSA------- --NVGVNRWR FLLESLEDLD 47**

**TrCRY3_XP_029687911 1:DNPALQEALN --------GA DS------LR CIYILDPWFA GAA------- --NVGINRWR FLLEALEDLD 47**

**BmCRY2_NP_001182627 1:DNPALREGII --------DA VT------FR CVFIIDPWFA SSS------- --NVGINKWR FLLQCLEDLD 47**

**DpCRY2_XP_032511492 1:DNPALREGLV --------DA TT------FR CVFIIDPWFA SSS------- --NVGINKWR FLLQCLEDLD 47**

**AgCRY2_ABB29887 1:DNPALREGLR --------GA RT------FR CVFIIDPWFA GSS------- --NVGINKWR FLLQCLDDLD 47**

**XtCRY4_NP_001123706 1:DNPTLVTALE --------TS DV------VY PVYILDRNFM TSS------- -SVIGSKRWN FFLQSIEDLH 48**

**XlCRY4_NP_001088990 1:DNPTLVAALE --------TS DI------IY PVYILDKNFM TSS------- -SVIGSKRWN FLLQSIEDLH 48**

**cCRY4_NP_001034685 1:DNPALLAALQ --------SS EV------VY PVYILDRAFM TSS------- -MHIGALRWH FLLQSLEDLR 48**

**zCRY4_NP_571862 1:DNPSLLGALA --------SS SA------LY PVYVLDRVFL QGA------- -MHMGALRWR FLLQSLEDLD 48**

**d(6-4)Phr_BAA12067 1:DNPALSHIFT --------AA NAAPGKYFVR PIFILDPGIL DWM------- --QVGANRWR FLQQTLEDLD 53**

**Dp(6-4)Phr_ABO38436 1:DNLALRNAIN --------EA ENR--KQILR PIYVIDPDIK N--------- --RVGCNRLR FLFQSLKNLD 49**

**Cg(6-4)Phr_XP_011414697 1:DNPALQAACK --------VA DD------VK PVFILDPWFA NNA------- --NVGVNRWR FLLQTLQNLD 47**

**Xt(6-4)Phr_LOC100144974 1:DNPALLAAMK --------DC AE------LY PIFILDPWFP RNM------- --KVSVNRWR FLIEALKDLD 47**

**Xl(6-4)Phr_NP_001081421 1:DNPALLAAMK --------DC AE------LH PIFILDPWFP KNM------- --QVSVNRWR FLIDALKDLD 47**

**z(6-4)Phr_NP_571863 1:DNPALIAALK --------DC RH------IY PLFLLDPWFP KNT------- --RIGINRWR FLIEALKDLD 47**

**Tr(6-4)Phr_XP_011607950 1:DNPGLMAALR --------DC KE------LY PVFILDPQL- HNK------- --SVGVNRCR FLIGALKDLD 46**

**At(6-4)Phr_NP_566520 1:DNPALEYASK --------GS EF------MY PVFVIDPHYM ESDPSAFSPG SSRAGVNRIR FLLESLKDLD 56**

**CrCRY(animal-like)_XP_042923874 1:DNPALLEACK --------DA KH------VY PVFVLDPHFL QQS------- SYKVSVNRYN FLLESLEDLQ 49**

**BmCRY1_NP_001182628 1:DNPSLHSALE E-------TS GP------FF PIFIFDGETA GTK------- --VVGYNRMR YLLEALDDLD 48**

**DpCRY1_XP_032522602 1:DNPSLHSALE D-------AS SP------FF PIFIFDGETA GTK------- --MVGYNRMR YLLEALNDLD 48**

**AgCRY1_ABB29886 1:DNPSLLEALK SDCVNQSSEA VK------LF PIFIFDGESA GTR------- --IVGYNRMK FLLESLADLD 55**

**dCRY_NP_732407 1:DNPALLAALA D-----KDQG IA------LI PVFIFDGESA GTK------- --NVGYNRMR FLLDSLQDID 50**

**CgCRY1_XP_034329330 1:DNPSLIDGLS --------EC DR------FY PVFIFDGEVA GTK------- --TAGYNRFR FLLECLQDLD 47**

**XtCRY-dash_XP_031759840 1:DNEVLHWAHR --------NA DQ------IV PLYCFDPRHY GGTHYF---N FPKTGPHRLK FLLESVQDLR 53**

**XlCRY-dash_NP_001084438 1:DNEVLHWAHR --------NA DQ------IV PLYCFDPRHY VGTHYF---N FPKTGPHRLK FLLESVRDLR 53**

**zCRY-dash_NP_991249 1:DNEVFHWAQR --------NA EH------II PLYCFDPRHY QGTYHY---N FPKTGPFRLR FLLDSVKDLR 53**

**TrCRY-dash_XP_003968037 1:DNELFHWAQR --------NA DH------IV PLYCFDPRHY MGTYHY---N LPKTGPFRLR FLLESIKDLR 53**

**XtCRY6_LC705158_(This_Study) 1:DNPALISALE --------HG VP------VI PVFLWCINEE TGQNFT---- -LATGGATKY WLHHALLKLN 51**

**XlCRY6_XP_018113364 1:DNPALVSALE --------HG VP------VI PVFLWCINEE TGQNFT---- -LATGGATKY WLHHALLELN 51**

**zCRY6_XP_009291670 1:DNPALIGCLE --------LG AP------VI PVFLWNAMEE EGPGVT---- -MSTGGASKY WLHQALVSLK 51**

**TrCRY6_XP_011605280 1:DNPALVSALK --------VG AP------VI PIFIWSPEEE EGPGVT---- -VAMGGACKY WLHQALSCLC 51**

**CgCRY6_AQM57607 1:DNPALFEAAS --------MN VP------VI LVFLWSESEE DPEGV----- -VAAGGATKL WLHHALNHLD 50**

**CrCRY(plant-like)_AAC37438 1:DNPALVAALA --------AA PN------VI PVFIWAPEEE GQF------- --QPGRCSRW WSKHSLVDLQ 47**

**AtCRY1_NP_567341 1:DNPALAAAVR --------AG P-------VI ALFVWAPEEE GHY------- --HPGRVSRW WLKNSLAQLD 46**

**AtCRY2_NP_171935 1:DNPALAAAAH --------EG S-------VF PVFIWCPEEE GQF------- --YPGRASRW WMKQSLAHLS 46**

**EcCPD_Phr_WP_001680736 1:DNLALAAACR --------NS SAR-----VL ALYIATPRQW ATH------- --NMSPRQAE LINAQLNGLQ 48**

**(Figure S2: 2/8)**

**cCRY1_NP_989576 48:ANL-RKLN-- -SRLFVIRGQ --------PA DVFPRLFKEW S-IAKLSIEY DSEPFGKERD AAIKKLASEA 104**

**XtCRY1_NP_001017311 48:ANL-RKLN-- -SRLFVIRGQ --------PA DVFPRLFKEW K-ITKLSIEY DSEPFGKERD AAIKKLASEA 104**

**XlCRY1_NP_001081129 48:ANL-RKLN-- -SRLFVIRGQ --------PA DVFPRLFKEW K-ITKLSIEY DSEPFGKERD AAIKKLASEA 104**

**mCRY1_NP_031797 48:ANL-RKLN-- -SRLFVIRGQ --------PA DVFPRLFKEW N-ITKLSIEY DSEPFGKERD AAIKKLATEA 104**

**zCRY1aa_NP_001070765 48:ASL-RKLN-- -SRLFVIRGQ --------PT DVFPRLFKEW N-INRLSYEY DSEPFGKERD AAIKKLANEA 104**

**zCRY1ab_BAA96847 48:ASL-RKLN-- -SRLFVIRGQ --------PT DVFPRLFKEW K-ISRLSYEY DSEPFGKDRD AAIRKLATEA 104**

**TrCRY1_XP_011611545 48:SSL-RKLN-- -SRLFVIRGQ --------PT DVFPRLFKEW N-ISRLSYEY DSEPFGKERD AAIKKLASEA 104**

**zCRY1ba_BAA96848 48:SNL-RKLN-- -SRLFVVRGQ --------PA NVFPRLFKEW K-ISRLTFEY DSEPFGKERD AAIKKLAMEA 104**

**TrCRY2_XP_011617050 48:ANL-RKLN-- -SRLFVIRGQ --------PA NVFPRLFKEW K-ISRLTFEY DSEPFGKERD AAIKKLAMEA 104**

**zCRY1bb_NP_571867 48:ASL-RKLN-- -SCLFVIRGQ --------PA DIFPRLFKEW K-VSRLTFEF DSEPFGKERD AAIKKLACEA 104**

**cCRY2_NP_989575 48:NSL-RKLN-- -SRLFVVRGQ --------PT DVFPRLFKEW G-VTRLTFEY DSEPFGKERD AAIIKLAKEA 104**

**mCRY2_NP_034093 48:TSL-RKLN-- -SRLFVVRGQ --------PA DVFPRLFKEW G-VTRLTFEY DSEPFGKERD AAIMKMAKEA 104**

**XtCRY2_XP_031756344 48:TSL-RKLN-- -SRLFVVRGQ --------PA DVFPRLFKEW G-VSRLTFEY DSEPFGKERD AVIMKLAKEA 104**

**XlCRY2_AAK94667 48:SSL-RKLN-- -SRLFVVRGQ --------PA DVFPKLFKEW G-VSRLTFEY DSEPFGKERD AVIMKLAKEA 104**

**zCRY2(zCRY3)_BAA96850 48:TSL-RKLN-- -SRLSVVRGQ --------PT DVFPRLFKEW N-VTRLTFEY DSEPYGKERD AAIIKMAQEY 104**

**TrCRY3_XP_029687911 48:CSL-RKLS-- -SRLLVVRGQ --------PT DVFPRLLKDW K-VTRLTFEF DPEPYGKERD GAIIKLAQQF 104**

**BmCRY2_NP_001182627 48:KSL-KKLN-- -SRLFVVRGQ --------PA DALPKLFREW G-TTALTFEE DPEPYGRVRD HNIISKCREV 104**

**DpCRY2_XP_032511492 48:KNL-RKLN-- -SRLFVVRGQ --------PA DALPKLFREW G-TTALTFEE DPEPYGRVRD HNIMTKCREV 104**

**AgCRY2_ABB29887 48:RNL-RKLN-- -SRLFVIRGQ --------PA DALPKLFKEW G-TTCLTFEE DPEPFGRVRD HNISEMCKEL 104**

**XtCRY4_NP_001123706 49:CNL-QKLN-- -SCLFVIQGD --------YE RVLREHVEKW N-ITQVTFDL EIEPYYKGLD ERIRAMGQEL 105**

**XlCRY4_NP_001088990 49:CNL-QKLN-- -SCLFVIQGD --------YQ SVLREHVQKW H-ITQVTFDL EIEPYYKGMD ERIRAMGQEL 105**

**cCRY4_NP_001034685 49:SSL-RQLG-- -SCLLVIQGE --------YE SVVRDHVQKW N-ITQVTLDA EMEPFYKEME ANIRGLGEEL 105**

**zCRY4_NP_571862 49:TRL-QAIG-- -SRLFVLCGS --------TA NILRELVAQW G-ITQISYDT EVEPYYTRMD KDIQTVAQEN 105**

**d(6-4)Phr_BAA12067 54:NQL-RKLN-- -SRLFVVRGK --------PA EVFPRIFKSW R-VEMLTFET DIEPYSVTRD AAVQKLAKAE 110**

**Dp(6-4)Phr_ABO38436 50:TSL-RKIN-- -TRLYVIKGK --------AI ECLPKLFDEW H-VKFLTLQV DIDADLVKQD EVIEEFCEAN 106**

**Cg(6-4)Phr_XP_011414697 48:ENL-KKIN-- -SRLYIIKGK --------PA DVFPKLFKNW G-VSHLTFEE DIEPYALTRD SAIKKLAEEH 104**

**Xt(6-4)Phr_LOC100144974 48:ENL-KKIN-- -SRLFVVRGK --------PT EVFPLLFKKW K-VTRLTFEV DTEPYSRQRD ADVEKLAAEH 104**

**Xl(6-4)Phr_NP_001081421 48:ENL-KKIN-- -SRLFVVRGK --------PA EVFPLLFKKW K-VTRLTFEV DIEPYSRQRD AEVEKLAAEH 104**

**z(6-4)Phr_NP_571863 48:SSL-KKLN-- -SRLFVVRGS --------PT EVLPKLFKQW K-ITRLTFEV DTEPYSQSRD KEVMKLAKEY 104**

**Tr(6-4)Phr_XP_011607950 47:LSL-RQLN-- -TRLFVVRGK --------PE EVFPKLFCQW K-ITKLTYEY DTEPLSLSRD KTVTRLAEEH 103**

**At(6-4)Phr_NP_566520 57:SSL-KKLG-- -SRLLVFKGE --------PG EVLVRCLQEW K-VKRLCFEY DTDPYYQALD VKVKDYASST 113**

**CrCRY(animal-like)_XP_042923874 50:RSF-QARG-- -SRLLVLRGK --------PE EVFPRVFREW G-VTQLCFEH DTEPYAKVRD AAVRRLAAEA 106**

**BmCRY1_NP_001182628 49:KQF-KKYG-- -GRLLLVKGK --------PS AVFRRLWEEF G-IRKLCFEQ DCEPVWRPRD ESVKTACREI 105**

**DpCRY1_XP_032522602 49:QQF-RKYG-- -GKLLMIKGR --------PD LIFRRLWEEF G-IRTLCFEQ DCEPIWRPRD ASVRALCRDI 105**

**AgCRY1_ABB29886 56:RQF-RDLG-- -GQLLVFRGD --------SV TVLRRLFEEL N-IKKLCYEQ DCEPIWKERD DAVAKLCRTM 112**

**dCRY_NP_732407 51:DQL-QAATDG RGRLLVFEGE --------PA YIFRRLHEQV R-LHRICIEQ DCEPIWNERD ESIRSLCREL 110**

**CgCRY1_XP_034329330 48:KNL-KAAG-- -TRLYCFQGQ --------PT DILERLIEEW G-VTKVTFEA DPEPIWQERD RLVRELLDKK 104**

**XtCRY-dash_XP_031759840 54:NTL-KERG-- -SNLLLRRGK --------PE EIIAGLVKQL GNVSAVTLHE EATKEETDVE SAVRRVCTQL 111**

**XlCRY-dash_NP_001084438 54:ITL-KKKG-- -SNLLLRRGK --------PE EVIEDLVKQL GNVSAVTLHE EATKEETDVE SAVKQACTRL 111**

**zCRY-dash_NP_991249 54:ALL-KKHG-- -STLLVRQGK --------PE DVVCELIKQL GSVSTVAFHE EVASEEKSVE EKLKEICCQN 111**

**TrCRY-dash_XP_003968037 54:NTL-LNKG-- -SNLIVRRGK --------PE EVVASLIKQL GSVSTVAFHE EVTSEELDVE KRVKDVCAQM 111**

**XtCRY6_LC705158_(This_Study) 52:QSLIQRFG-- -SHIIFRVAR -------SCE EELVSLVHET G-ADTIVINA VYEPWLKERD DLISETLRRH 110**

**XlCRY6_XP_018113364 52:QSLIQKFG-- -SHVIFRVAQ -------SCE KELVSLVHET G-AGTVIVNA VYEPWLKERD DFISENLQKN 110**

**zCRY6_XP_009291670 52:RSL-EERG-- -SHLVTLKAE P------SSL TALQGLMDET G-AASVVATA LYEPWLKERD DALWETLEKR 110**

**TrCRY6_XP_011605280 52:SSL-ENIG-- -SHLVFLKSE TRGSEGGASL RALQGLAKAT G-ARTVVANA LYEPWLKGRD DAVAAALQKS 116**

**CgCRY6_AQM57607 51:KSISDRYN-- -NRIIYRKTQ -------SCQ REILSLIEET G-AKALLIND VYEPFLKQRD DKICSELQRK 109**

**CrCRY(plant-like)_AAC37438 48:QAL-AALG-- -SRLVIRRST -------DST AALLQLVTEL G-AEAVFFNH LYDPISLMRD HDCKRGLTAA 105**

**AtCRY1_NP_567341 47:SSL-RSLG-- -TCLITKRST -------DSV ASLLDVVKST G-ASQIFFNH LYDPLSLVRD HRAKDVLTAQ 104**

**AtCRY2_NP_171935 47:QSL-KALG-- -SDLTLIKTH -------NTI SAILDCIRVT G-ATKVVFNH LYDPVSLVRD HTVKEKLVER 104**

**EcCPD_Phr_WP_001680736 49:IAL-AEKG-- -IPLLFREVD D----FVASV EIVKQGCAEN S-VTHLFYNY QYEVNERARD VEVERALR-- 107**

**(Figure S2: 3/8)**

**cCRY1_NP_989576 105:GVEVIVRISH TLYDLDKIIE LNGG-QPPLT YKRFQTLISR MEPL--EMPV ETIT--PEVM QKCTTPVSDD 169**

**XtCRY1_NP_001017311 105:GVEVIVRISH TLYDLDKIIE LNGG-QPPLT YKRFQTLISK MEPL--EIPV ETIT--AEVM EKCTTPVLDD 169**

**XlCRY1_NP_001081129 105:GVEVIVRISH TLYDLDKIIE LNGG-QPPLT YKRFQTLISK MDPL--EIPV ETIT--AEVM EKCTTPVSDD 169**

**mCRY1_NP_031797 105:GVEVIVRISH TLYDLDKIIE LNGG-QPPLT YKRFQTLVSK MEPL--EMPA DTIT--SDVI GKCMTPLSDD 169**

**zCRY1aa_NP_001070765 105:GVEVIVRISH TLYDLDKIIE LNGG-QSPLT YKRFQTLISR MEAV--ETPA ETIT--AEVM GPCTTPLSDD 169**

**zCRY1ab_BAA96847 105:GVEVFVRISH TLYDLDKIIE FNGG-QSPLT YKRFQTLISR MDPV--EMPA ETIT--AEIM GKCSTPVSDD 169**

**TrCRY1_XP_011611545 105:GVEVTVCISH TLYDLDKIIE LNGG-QSPLT YKRFQTLISR MDPV--EVPA ESIT--AEIM GKCTTPLSDD 169**

**zCRY1ba_BAA96848 105:GVEVIVKTSH TLYNLDKIIE LNGG-QPPLT YKRFQTLISR MDPP--EMPV ETLS--NSIM GCCVTPVAED 169**

**TrCRY2_XP_011617050 105:GVEVIVKISH TLYDLDKIIE LNGG-HPPLT YKRFQTLISR MDPP--EMPV ETLS--GNLM GRCVTPISED 169**

**zCRY1bb_NP_571867 105:GVEVIVKISH TLYDLDRIIE LNGG-QSPLT YKRFQTLVSS MEPP--DPPL ASPD--RGMM GKCVTPISEN 169**

**cCRY2_NP_989575 105:GVEVVIENSH TLYDLDRIIE LNGN-KPPLT YKRFQAIISR MELP--KKPV SSIV--SQQM ETCKVDIQEN 169**

**mCRY2_NP_034093 105:GVEVVTENSH TLYDLDRIIE LNGQ-KPPLT YKRFQALISR MELP--KKPA VAVS--SQQM ESCRAEIQEN 169**

**XtCRY2_XP_031756344 105:GVEVIVENSH TLYDLDRIIE LNGH-SPPLT YKRFQAIISR MELP--RRPA PSVT--RQQM EACRAEIKRN 169**

**XlCRY2_AAK94667 105:GVEVIVENSH TLYDLDRVIE LNGH-SPPLT YKRFQAIISR MELP--RRPA PSVT--RQQM EACRAEIKRN 169**

**zCRY2(zCRY3)_BAA96850 105:GVETVVRNSH TLYNPDRIIE MNNH-SPPLT FKRFQAIVNR LELP--RKPL PTIT--QEQM ARCRTQISDN 169**

**TrCRY3_XP_029687911 105:GVETIVRNSH TLYNLDRIIE VNNN-SPPLT FKRFQTIVSR LELP--RRPL PTVT--QHQI HKCGAKMADS 169**

**BmCRY2_NP_001182627 105:GITVTSRVSH TLYKLDKIIE RNGG-KAPLT YHQFQALIAS MPPP--PPAE VTIT--PQML NGATTPITDN 169**

**DpCRY2_XP_032511492 105:GIQVTSRVSH TLYKLDDIIE KNGG-KAPLT YHQFQALIAS MPPP--PSAE PTIS--LETL NRAVTPISDN 169**

**AgCRY2_ABB29887 105:GIEVISAASH TLYNLERIIE KNGG-RAPLT YHQFQAIIAS MDAP--PQPE AAIT--LDVI GNANTPQYDD 169**

**XtCRY4_NP_001123706 106:GFEVVSMVAH TLYDIKKILA LNCG-KPPLT YKNFLRVLSM LGNP--DKPA RQIT--SEDF IKCITPTKLA 170**

**XlCRY4_NP_001088990 106:GFDVVSKVAH TLYDVKSILA LNYG-KPPLT YKNFLRVLSV LGDP--DKPA RQIT--LEDF IKCTTPTEFA 170**

**cCRY4_NP_001034685 106:GFQVLSLMGH SLYNTQRILE LNGG-TPPLT YKRFLRILSL LGDP--EVPV RNPT--AEDF QRCS-PPELG 169**

**zCRY4_NP_571862 106:GLQTYTCVSH TLYDVKRIVK ANGG-SPPLT YKKFLHVLSV LGEP--EKPA RDVS--IEDF QRCVTPV--D 168**

**d(6-4)Phr_BAA12067 111:GVRVETHCSH TIYNPELVKA KNLG-KAPIT YQKFLGIVEQ LKVP--KV-- -LGV--PEKL KKMPTPPKDE 172**

**Dp(6-4)Phr_ABO38436 107:NIFVVKRMQH TVYDFNSVVK KNNG-SIPLT YQKFLSLVSD VQVK----DI IQIS--KGVS DECK---ASD 166**

**Cg(6-4)Phr_XP_011414697 105:NVKVTSCVSH TLFDPQRIIS KNGG-KAPLT YQRLQTVLSS LGSP--PKPV DSPS------ -ECKTKTESD 164**

**Xt(6-4)Phr_LOC100144974 105:NVQVIQKVSN TLYAIDRIIA ENNG-KPPLT YVRFQTVLAS LGPP--KRPV QVPT--QENM KDCCTLWKSS 169**

**Xl(6-4)Phr_NP_001081421 105:DVQVIQKVSN TLYDIDRIIA ENNG-KPPLT YVRFQTVLAP LGPP--KRPI KAPT--LENM KDCHTPWKSS 169**

**z(6-4)Phr_NP_571863 105:GVEVTPKISH TLYNIDRIID ENNG-KTPMT YIRLQSVVKA MGHP--KKPI PAPT--NEDM RGVSTPLSDD 169**

**Tr(6-4)Phr_XP_011607950 104:GIDVVCKVSH TLFDINRIIE ENNG-KTPLT YKSMQAIVKK LGPP--KRPL SAPS--MEDL KDVNTPCSES 168**

**At(6-4)Phr_NP_566520 114:GVEVFSPVSH TLFNPAHIIE KNGG-KPPLS YQSFLKVA-- -GEP--SCAK SELV-----M SYSSLPPIGD 172**

**CrCRY(animal-like)_XP_042923874 107:GVEVVTPISH TLYDTDMLVA RNGG-AAPLT MQSFTKLVDR VGDP--PAPA PDPP------ -AAMPPPAED 166**

**BmCRY1_NP_001182628 106:GVTCREHVSH TLWEPDTVIK ANGG-IPPLT YQMFLHTVAT IGDP--PRPV DNAK--LRGI KFGTLPLCFY 170**

**DpCRY1_XP_032522602 106:GVSCREHVAH TLWNPDTVIK ANGG-IPPLT YQMFLHTVEI IGNP--PRPV DDVD--LNGV NFGSLPESFY 170**

**AgCRY1_ABB29886 113:DVRCVENVSH TLWNPIEVIQ TNGD-IPPLT YQMFLHTVNI IGDP--PRPV GAPN--FEYV EFGRVPALLA 177**

**dCRY_NP_732407 111:NIDFVEKVSH TLWDPQLVIE TNGG-IPPLT YQMFLHTVQI IGLP--PRPT ADAR--LEDA TFVELDPEFC 175**

**CgCRY1_XP_034329330 105:NVQCVEKVSH TLWDPYEIIE NNGG-SPPLT FSLFNLVTST IGPP--PRPV EDPD-----F TDISLPVSQN 166**

**XtCRY-dash_XP_031759840 112:GVRYQTFWGS TLYHREDLPF RHIS-SLPDV YTQFRKAAET QGKV--RSTF QMPD-R---- ---LKPLPSG 170**

**XlCRY-dash_NP_001084438 112:GIKYQTFWGS TLYHREDLPF RHIS-SLPDV YTQFRKAVET QGKV--RPTF QMPD-K---- ---LKPLPSG 170**

**zCRY-dash_NP_991249 112:KVRVQTFWGS TLYHRDDLPF SHIG-GLPDV YTQFRKAVEA QGRV--RPVL STPE-Q---- ---VKSPPSG 170**

**TrCRY-dash_XP_003968037 112:KVNVHTCWGS TLYHRDDLPF HHIS-RLPDV YTQFRKAVES QCRV--RPVF PPPE-H---- ---LKPLPQG 170**

**XtCRY6_LC705158_(This_Study) 111:GVELKKHHSY CLYEPDSVST EGVGLRGIGS VSHFMSCCKR NNSAPIGMPL DAPR--CLP- APCNWPESDH 177**

**XlCRY6_XP_018113364 111:GVEFKKHHSY CLYEPGSIST EGVGLRGIGS VSHFMSCCKR NQSAPIGIPL DAPM--ILP- TPCKWPDSDD 177**

**zCRY6_XP_009291670 111:GVTCHIYHSY CLRDPYTVST RGVGLRGIGS VSHFMSCCQQ NPAGGLGSPL DAPT--TLP- SPSAWPQGCP 177**

**TrCRY6_XP_011605280 117:GVECEMFHSY CLRDPYSVST EGVGLRGIGS VSHFMSCCRQ NPGAALGAPL DPPT--TLP- VPAHWPPGVS 183**

**CgCRY6_AQM57607 110:GIECKRFHSY LLHEPGSVSA ESVGMRGVGS VTHFMECCRQ SDAQPIGHPL DYPP--TLP- KPDQFPSSSS 176**

**CrCRY(plant-like)_AAC37438 106:GVAHRTFNGD MLYEPWDVLD PNK--QPYST FDDFWNSVRA MPVPP-PFPV SAPA--SMPA VPAAVPSMTV 170**

**AtCRY1_NP_567341 105:GIAVRSFNAD LLYEPWEVTD ELG--RPFSM FAAFWERCLS MPYDP-ESPL LPPK------ KIISGDVSKC 165**

**AtCRY2_NP_171935 105:GISVQSYNGD LLYEPWEIYC EKG--KPFTS FNSYWKKCLD MSI---ESVM LPPPWRLMPI TAAAEAIWAC 169**

**EcCPD_Phr_WP_001680736 108:NVVCEGFDDS VILPPGAVMT GNH--EMYKV FTPFKNAWLK RLREGMPECV AAPK--VRS- SGSIEPSPS- 171**

**(Figure S2: 4/8)**

**cCRY1_NP_989576 170:HDEK----YG VPSLEELGFD ----TDGLPS AVWPGGETEA LTRLERHL-- -ERKAWVANF ERPRMNANSL 228**

**XtCRY1_NP_001017311 170:HDEK----YG VPSLEELGFD ----TEGLPS AVWPGGETEA LTRLERHL-- -ERKAWVANF ERPRMNANSL 228**

**XlCRY1_NP_001081129 170:HDEK----YG VPSLEELGFD ----TEGLPS AVWPGGETEA LTRLERHL-- -ERKAWVANF ERPRMNANSL 228**

**mCRY1_NP_031797 170:HDEK----YG VPSLEELGFD ----TDGLSS AVWPGGETEA LTRLERHL-- -ERKAWVANF ERPRMNANSL 228**

**zCRY1aa_NP_001070765 170:HDEK----FG VPSLEELGFD ----TEGLSS AVWPGGETEA LTRLERHL-- -ERKAWVANF ERPRMNANSL 228**

**zCRY1ab_BAA96847 170:HDNK----FG VPSLEELGFE ----TEGLST AVWPGGETEA LTRLERHL-- -ERKAWVANF ERPRMNANSL 228**

**TrCRY1_XP_011611545 170:HDDK----YG VPSLEELGFD ----TEGLSS AVWPGGETEA LTRLERHL-- -ERKAWVANF ERPRMNANSL 228**

**zCRY1ba_BAA96848 170:HGDK----YG VPSLEELGFD ----IEGLPS AVWPGGETEA LTRIERHL-- -ERKAWVANF ERPRMNANSL 228**

**TrCRY2_XP_011617050 170:HGEK----YG VPSLEELGFD ----IEGLPS AVWPGGETEA LTRIERHL-- -ERKAWVANF ERPRMNANSL 228**

**zCRY1bb_NP_571867 170:HRDK----YG VPLLEELGFD ----TEGLAP AVWPGGESEA LKRMERHLGP DSTVAWQENF ERPKMNASPL 231**

**cCRY2_NP_989575 170:HDDV----YG VPSLEELGFP ----TDGLAP AVWQGGETEA LARLDKHL-- -ERKAWVANY ERPRMNANSL 228**

**mCRY2_NP_034093 170:HDDT----YG VPSLEELGFP ----TEGLGP AVWQGGETEA LARLDKHL-- -ERKAWVANY ERPRMNANSL 228**

**XtCRY2_XP_031756344 170:HDET----YG VPSLDELGFH ----SEIKGP SIWPGGETEA LARLDRHL-- -ERKAWVANY ERPRMNANSL 228**

**XlCRY2_AAK94667 170:HDET----YG VPSLEELGFH ----SENKGH AIWPGGETEA LARLDRHL-- -ERKAWVANY ERPRMSANSL 228**

**zCRY2(zCRY3)_BAA96850 170:HDEH----YG VPSLEELGFR ----TQGDSL HVWKGGETEA LERLNKHL-- -DRKAWVANF ERPRISGQSL 228**

**TrCRY3_XP_029687911 170:QEQL----YS IPSLEELGFR ----TEGLPP AVWRGGESEA LERLHKHL-- -DKKVWVANL EHSRVSTCSL 228**

**BmCRY2_NP_001182627 170:HDDR----FG VPTLEELGFE ----TEGLKP PIWIGGESEA LARLERHL-- -ERKAWVASF GRPKMTPQSL 228**

**DpCRY2_XP_032511492 170:HDER----FG VPTLEELGFD ----TEGLKP PIWIGGENEA LLRLERHL-- -ERKAWVASF GRPKMTPESL 228**

**AgCRY2_ABB29887 170:HDDK----YG VPTLEELGFE ----TEALRP PVWIGGETEA LARLERHL-- -ERKAWVASF GRPKMTPQSL 228**

**XtCRY4_NP_001123706 171:AEEY----YR IPKPEDLGIS ----KDC--P TNWIGGESEA LSRLEQHL-- -EKQGWVANF KKPQTIPNSL 227**

**XlCRY4_NP_001088990 171:AEEY----YR IPKPEDLGIC ----RDC--A PNWKGGESEA LCRLEQHL-- -EKQGWVANF QKPQTVPNSL 227**

**cCRY4_NP_001034685 170:LAEC----YG VPLPTDLKIP ----PES--I SPWRGGESEG LQRLEQHL-- -ADQGWVASF TKPKTVPNSL 226**

**zCRY4_NP_571862 169:VDRV----YA VPSLAHLGLQ ----VEA--E VLWPGGESHA LQRLEKHF-- -QSQGWVANF SKPRTIPNSL 225**

**d(6-4)Phr_BAA12067 173:VEQKDSAAYD CPTIKQLVKR ----PEELGP NKFPGGETEA LRRMEESL-- -KDEIWVARF EKPNTAPNSL 235**

**Dp(6-4)Phr_ABO38436 167:YDSQ---GYD IPSLEEFGVN ----ESELSE CKYPGGESEG LKRLDVYM-- -AKKQWVCNF EKPKSSPNSI 226**

**Cg(6-4)Phr_XP_011414697 165:HDKK----YG VPSLEDLGKS ----EKECGP LLFPGGETEA LRRLESMM-- -GKKNWVCTF EKPKTAPNSL 223**

**Xt(6-4)Phr_LOC100144974 170:YNEK----YG VPTLEELGQD ----SLKLGP RLYPGGESEA LSRLELHM-- -KRTTWVCNF KKPETEPNSL 228**

**Xl(6-4)Phr_NP_001081421 170:YDEK----YG VPTLEELGQD ----PMKLGP HLYPGGESEA LSRLDLHM-- -KRTSWVCNF KKPETEPNSL 228**

**z(6-4)Phr_NP_571863 170:HEEK----FG IPTLEDLGLD ----TSSLGP HLFPGGEQEA LRRLDEHM-- -ERTNWVCKF EKPKTSPNSL 228**

**Tr(6-4)Phr_XP_011607950 169:HEKK----YR IPTLEDFGHN ----LADLPE EQFPGGEQEA LRRLEEHM-- -KRTAWVCNF EKPKTSPNSL 227**

**At(6-4)Phr_NP_566520 173:IGNL--GISE VPSLEELGYK ---DDEQADW TPFRGGESEA LKRLTKSI-- -SDKAWVANF EKPKGDPSAF 234**

**CrCRY(animal-like)_XP_042923874 167:MPSAAPAATG VPTWQEVGFK ----EPP--L TVFKGGETEA LARLEAAF-- -QDPKWVAGF QKPDTDPSAW 227**

**BmCRY1_NP_001182628 171:EEFT--VYDK VPNPEDLGVF LE--NEDIRM IRWVGGETAA LKQMQHRLAV -EYETFCRGS YLPTHGSPDL 235**

**DpCRY1_XP_032522602 171:REFV--VFDK APKPEDLGVF LE--NEDIRM IRWVGGETAA LKQMQERLAV -EYETFCRGS YLPTHGNPDL 235**

**AgCRY1_ABB29886 178:SELK--LCQQ MPAPDDFGIH YD-GNARIAF QKWIGGETRA LEALGARLKQ -EEEAFREGY YLPTQAKPEI 243**

**dCRY_NP_732407 176:RSLK--LFEQ LPTPEHFNVY GD-NMGFLAK INWRGGETQA LLLLDERLKV -EQHAFERGF YLPNQALPNI 241**

**CgCRY1_XP_034329330 167:HDKQ----FG IPTLEDLNVR PECEEQNKRL VEWLGGESKA LELLAIRMKH -EEKAYENGY VMPNQYHPDL 231**

**XtCRY-dash_XP_031759840 171:LEEG-----S VPTHQDFDQQ DP-LTDPRSA FPCCGGETQA LQRLHHYF-- -WETNLVASY KDTRNGLIGI 231**

**XlCRY-dash_NP_001084438 171:LEEG-----S VPSHEDFDQQ DP-LTDPRTA FPCSGGESQA LQRLEHYF-- -WETNLVASY KDTRNGLIGL 231**

**zCRY-dash_NP_991249 171:LEEG-----P IPTFDSLGQT EP-LDDCRSA FPCRGGETEA LARLKHYF-- -WDTNAVATY KETRNGMIGV 231**

**TrCRY-dash_XP_003968037 171:LEEG-----T ILTAEDLEQK EP-VADPRSA FPCSGGESQA LARLKHYF-- -WDTDAVAVY KETRNGLIGV 231**

**XtCRY6_LC705158_(This_Study) 178:LDTL--ELGK MPHRKDGTLI ---DWAVTIR ESWDFSEDGA YTCLANFL-- -QDG--VKHY EKESGRADK- 236**

**XlCRY6_XP_018113364 178:LDKM--KLAK MPSRKDGTLI ---DWAMTIR ESWDFSEDGA YKCLSNFL-- -EDG--VKHY EKESGRADK- 236**

**zCRY6_XP_009291670 178:LADL--GLAR MPRRKDGTVI ---DWAVDIR KTWDFSEEGA HTHLEAFL-- -RDG--VYRY EKESCRADA- 236**

**TrCRY6_XP_011605280 184:LETL--GLAR MPKRKDGTTI ---DWAANIR KSWDFSEGGA HARLEAFL-- -HDG--VYRY EKESGRADA- 242**

**CgCRY6_AQM57607 177:LHDL--ELAK MPRRKDGSII ---DWAAPIV RQWDFGEEGA WKALELFL-- -SEG--VRKY EKESCRTDH- 235**

**CrCRY(plant-like)_AAC37438 171:AEVD---WFF TPEQE----- ---ASSDQLK FKWKPGVGGA ISELEHFL-- -AER--LTEF EHDRAKVDR- 223**

**AtCRY1_NP_567341 166:VADP--LVFE DDSEK----- ---GSNALLA RAWSPGWSNG DKALTTFI-- -NGP--LLEY SKNRRKADS- 219**

**AtCRY2_NP_171935 170:SIEE--LGLE NEAEK----- ---PSNALLT RAWSPGWSNA DKLLNEFI-- -EKQ--LIDY AKNSKKVVG- 223**

**EcCPD_Phr_WP_001680736 172:------ITLN YP-------- ----RQSFDT AHFPVEEKAA IAQLRQFC-- -QNG--AGEY EQQRDFPAV- 217**

**(Figure S2: 5/8)**

**cCRY1_NP_989576 229:L-ASPTGLSP YLRFGCLSCR LFYFKLTDLY KKVKKN---- -------SSP PLSLYGQLLW REFFYTAATN 286**

**XtCRY1_NP_001017311 229:L-ASTTGLSP YLRFGCLSCR LFYFKLTDLY KKVKKN---- -------SSP PLSLYGQLLW REFFYTAATN 286**

**XlCRY1_NP_001081129 229:L-ASTTGLSP YLRFGCLSCR LFYFKLTDLY KKVKKN---- -------SSP PLSLYGQLLW REFFYTAATN 286**

**mCRY1_NP_031797 229:L-ASPTGLSP YLRFGCLSCR LFYFKLTDLY KKVKKN---- -------SSP PLSLYGQLLW REFFYTAATN 286**

**zCRY1aa_NP_001070765 229:L-ASPTGLSP YLRFGCLSCR LFYFKLTDLY RKVKKN---- -------SSP PLSLYGQLLW REFFYTAATN 286**

**zCRY1ab_BAA96847 229:L-ASPTGLSP YLRFGCLSCR LFYFKLTDLY RKVKKN---- -------STP SLSLYGQLLW REFFYTAATN 286**

**TrCRY1_XP_011611545 229:L-ASPTGLSP YLRFGCLSCR LFYFKLTDLY RKVKKN---- -------SSP PLSLYGQLLW REFFYTAATN 286**

**zCRY1ba_BAA96848 229:L-ASPTGLSP YLRFGCLSCR LFYFKLTDLY RKVKKT---- -------STP PLSLYGQLLW REFFYTAATT 286**

**TrCRY2_XP_011617050 229:L-ASPTGLSP YLRFGCLSCR LFYFKLTDLY RKVKKN---- -------SSP PLSLYGQLLW REFFYTAATN 286**

**zCRY1bb_NP_571867 232:M-ASPLGLSP YLRFGCLSCR LFYCKLTQLY KKVKKN---- -------MNP SISLYDKILW REFFYTAATN 289**

**cCRY2_NP_989575 229:L-ASPTGLSP YLRFGCLSCR LFYYRLWELY KKVKRN---- -------STP PLSLYGQLLW REFFYTAATN 286**

**mCRY2_NP_034093 229:L-ASPTGLSP YLRFGCLSCR LFYYRLWDLY KKVKRN---- -------STP PLSLFGQLLW REFFYTAATN 286**

**XtCRY2_XP_031756344 229:L-ASPTGLSP YLRFGCLSCR LFYYRLKELY KKVKKN---- -------SPP PLSLYGQLLW REFFYTAATN 286**

**XlCRY2_AAK94667 229:L-ASPTGLSP YLRFGCLSCR LFYYRLQELY QKVKKN---- -------SPP PLSLYGQLLW REFFYTAATN 286**

**zCRY2(zCRY3)_BAA96850 229:F-PSPTGLSP YLRFGCLSCR VFYYNLRDLF MKLRRR---- -------SSP PLSLFGQLLW REFFYTAGTN 286**

**TrCRY3_XP_029687911 229:Y-ASPAGLSP YLRFGCLSCR VLYYNLRELY VKLRKG---- -------CSP PPSLFGQLLW REFFYTAATN 286**

**BmCRY2_NP_001182627 229:L-ASQTGLSP YLRFGCLSTR LFYYQLTELY KRVKR----- -------VRP PLSLHGQILW REFFYCAATR 285**

**DpCRY2_XP_032511492 229:L-SSQTGLSP YLRFGCLSTR LFYYQLSELY KRIKQ----- -------ERP PLSLHGQILW REFFYCAATR 285**

**AgCRY2_ABB29887 229:L-ASQTGLSP YLRFGCLSTR LFYYQLTDLY KKIKK----- -------ACP PLSLHGQLLW REFFYCAATK 285**

**XtCRY4_NP_001123706 228:L-PSTTGLSP YFSLGCLSVR VFFHRLSNIY AQSKNH---- -------SLP PVSLQGQLLW REFFYTVASS 285**

**XlCRY4_NP_001088990 228:L-PSTTGLSP YFSFGCLSAR VFYHRLSNIY AQSKNH---- -------SLP PVSLQGQLLW REFFYTAASS 285**

**cCRY4_NP_001034685 227:L-PSTTGLSP YFSTGCLSVR SFFYRLSNIY AQAKHH---- -------SLP PVSLQGQLLW REFFYTVASA 284**

**zCRY4_NP_571862 226:L-PSTTGLSP YLSLGCLSVR TFYHRLNCIY AQSKNH---- -------SLP PVSLQGQVLW REFFYTVASA 283**

**d(6-4)Phr_BAA12067 236:E-PSTTVLSP YLKFGCLSAR LFNQKLKEII KRQPKH---- -------SQP PVSLIGQLMW REFYYTVAAA 293**

**Dp(6-4)Phr_ABO38436 227:E-PSTTVLSP YISHGCLSAK LFYHKLKQVE NG-SKH---- -------TLP PVSLMGQLMW REFYYTAGSG 283**

**Cg(6-4)Phr_XP_011414697 224:E-PSTTVLSP YLKFGCLSPR MFYYKLQEVY NK-AKH---- -------TSP PVSLLGQLLW REFYYCVAVD 280**

**Xt(6-4)Phr_LOC100144974 229:T-PSTTVLSP YVKFGCLSAR TFWWRIAEIY QG-KKH---- -------SDP PVSLHGQLLW REFFYTAGVG 285**

**Xl(6-4)Phr_NP_001081421 229:T-PSTTVLSP YVKFGCLSAR TFWWKIADIY QG-KKH---- -------SDP PVSLHGQLLW REFYYTTGAG 285**

**z(6-4)Phr_NP_571863 229:I-PSTTVLSP YVRFGCLSAR TFWWRLADVY RG-KTH---- -------SDP PVSLHGQLLW REFFYTTAVG 285**

**Tr(6-4)Phr_XP_011607950 228:S-PSTTVLSP YVTFGCLSVR TFWWRLSDVY EG-KKH---- -------SAP PVSLHGQLLW REFFYTASVG 284**

**At(6-4)Phr_NP_566520 235:LKPATTVMSP YLKFGCLSSR YFYQCLQNIY KDVKKH---- -------TSP PVSLLGQLLW REFFYTTAFG 293**

**CrCRY(animal-like)_XP_042923874 228:EKPATTVLSP YLKFGCLSAR LFHARLLEVY RRHPAH---- -------SQP PVSLRGQLLW REFFYTVGST 286**

**BmCRY1_NP_001182628 236:L-GPPISLSP ALRFGCLSVR KFYWSLQDLF QQVHQGS--- -------LCS TQYITGQLIW REYFYTMSVN 294**

**DpCRY1_XP_032522602 236:L-GPPISLSP ALRFGCLSVR RFYWSLQDLF QQVHQGR--- -------LAS TQFITGQLIW REYFYTMSVN 294**

**AgCRY1_ABB29886 244:L-GPATSMSA ALRFGCLSVR MFYWCVHDLF AKVQSNS--- QFK----YPG GHHITGQLIW REYFYTMSVQ 305**

**dCRY_NP_732407 242:H-DSPKSMSA HLRFGCLSVR RFYWSVHDLF KNVQLRACVR GVQ----MTG GAHITGQLIW REYFYTMSVN 306**

**CgCRY1_XP_034329330 232:L-SPPLSLSA HLRFGCLSVR KFYWSIHDKF EEQNTRSTAL HFKVKPSMGA PVSLSAQLMW REYFYTMAIN 300**

**XtCRY-dash_XP_031759840 232:--DYSTKFAP WLALGCISPR YIYEQIRKYE KERTA----- -------NQS TYWVIFELLW RDYFRFVALK 287**

**XlCRY-dash_NP_001084438 232:--DYSTKFAP WLALGCVSPR YIYEQIGKYE KERTA----- -------NQS TYWVIFELLW RDYFRFVALK 287**

**zCRY-dash_NP_991249 232:--DFSTKFSP WLALGCISPR YIYEQIKKYE VERTA----- -------NQS TYWVIFELLW RDYFKFVALK 287**

**TrCRY-dash_XP_003968037 232:--DYSTKFSP WLALGCISPR YIYHQIKQYE SERTA----- -------NQS TYWVIFELLW RDYFRFVAVK 287**

**XtCRY6_LC705158_(This_Study) 237:--PYTSHISP YLHFGQISPR TVLHEAYFT- ---------- -------KKN VPKFLRKLAW RDLAYWLLIL 286**

**XlCRY6_XP_018113364 237:--PYTSHISP YLHFGQISPR TVLHEAYFT- ---------- -------KKN VPKFLRKLAW RDLAYWLLLL 286**

**zCRY6_XP_009291670 237:--PNTSCLSP YLHFGQLSAR QVLWAARGA- ---------- -------RCK SPKFQRKLAW RDLAYWQISL 286**

**TrCRY6_XP_011605280 243:--PNTSCVSP YLHFGQLSPR WLLWDAKGA- ---------- -------RCR PPKFQRKLAW RDLAYWQLTL 292**

**CgCRY6_AQM57607 236:--LNTCRISP YLHFGQISPR AVLEEAR--- ---------- -------HMK SPKFLRKLAW RDLSYWLLTL 283**

**CrCRY(plant-like)_AAC37438 224:--DSTSRLSP WIHIGSISVR YIFYRVRQCQ AEWLAAGT-- -----DRAQS CDDFLQQMGY REYSRYLAFH 284**

**AtCRY1_NP_567341 220:--ATTSFLSP HLHFGEVSVR KVFHLVRIKQ VAWANEGN-- ----EAGEES VNLFLKSIGL REYSRYISFN 281**

**AtCRY2_NP_171935 224:--NSTSLLSP YLHFGEISVR HVFQCARMKQ IIWARDKN-- ----SEGEES ADLFLRGIGL REYSRYICFN 285**

**EcCPD_Phr_WP_001680736 218:--EGTSRLSA SLATGGLSPR QCLHRLLAEQ PQALD----- -------GGA GSVWLSELIW REFYRHLMTY 273**

**(Figure S2: 6/8)**

**cCRY1_NP_989576 287:NPRFDKMEGN PICVQ-IPWD K-NPEALAKW AEGRTGFPWI DAIMTQLRQE GWIHHLARHA VACFLTRGDL 354**

**XtCRY1_NP_001017311 287:NPRFDKMEGN PICVQ-IPWD R-NPEALAKW AEGRTGFPWI DAIMTQLRQE GWIHHLARHA VACFLTRGDL 354**

**XlCRY1_NP_001081129 287:NPRFDKMDGN PICVQ-IPWD R-NPEALAKW AEGRTGFPWI DAIMTQLRQE GWIHHLARHA VACFLTRGDL 354**

**mCRY1_NP_031797 287:NPRFDKMEGN PICVQ-IPWD K-NPEALAKW AEGRTGFPWI DAIMTQLRQE GWIHHLARHA VACFLTRGDL 354**

**zCRY1aa_NP_001070765 287:NPRFDKMEGN PICVQ-IPWD K-NPEALAKW AEGRTGFPWI DAIMTQLRQE GWIHHLARHA VACFLTRGDL 354**

**zCRY1ab_BAA96847 287:NPHFDKMEFN PICVQ-IPWD R-NPEALAKW AEGQTGFPWI DAIMTQLRQE GWIHHLARHA VACFLTRGDL 354**

**TrCRY1_XP_011611545 287:NPCFDKMENN PICVQ-IPWD R-NPEALAKW AEGRTGFPWI DAIMTQLRQE GWIHHLARHA VACFLTRGDL 354**

**zCRY1ba_BAA96848 287:NPRFDKMEGN PICVR-IPWD K-NPEALAKW AEAKTGFPWI DAIMTQLRQE GWIHHLARHA VACFLTRGDL 354**

**TrCRY2_XP_011617050 287:NPRFDKMEGN PICVR-IPWD R-NMEALAKW AEAKTGFPWI DAIMTQLRQE GWIHHLARHA VACFLTRGDL 354**

**zCRY1bb_NP_571867 290:NPRFDRMEGN PICIR-IPWD R-NAEALAKW AEAKTGFPWI DAIMMQLRQE GWIHHLARHA VACFLTRGDL 357**

**cCRY2_NP_989575 287:NPKFDRMEGN PICIQ-IPWD K-NPEALAKW AEGKTGFPWI DAIMTQLRQE GWIHHLARHA VACFLTRGDL 354**

**mCRY2_NP_034093 287:NPRFDRMEGN PICIQ-IPWD R-NPEALAKW AEGKTGFPWI DAIMTQLRQE GWIHHLARHA VACFLTRGDL 354**

**XtCRY2_XP_031756344 287:NPKFDQMEGN PICVQ-IPWD K-NPKALAKW AEGKTGFPWI DAIMTQLRQE GWIHHLARHA VACFLTRGDL 354**

**XlCRY2_AAK94667 287:NPKFDQMEGN PICVQ-IPWD K-NPKALAKW TEGKTGFPWI DAIMTQLRQE GWIHHLARHA VACFLTRGDL 354**

**zCRY2(zCRY3)_BAA96850 287:NPNFDHMEGN PICVQ-IPWD H-NPEALAKW AEGRTGFPWI DAIMTQLRQE GWIHHLARHA VACFLTRGDL 354**

**TrCRY3_XP_029687911 287:NPNFDRMEGN PICVQ-IPWD Q-NPEALAKW AEGHTGFPWI DAIMTQLRQE GWIHHQARRA VACFLTRGDL 354**

**BmCRY2_NP_001182627 286:NPNFDRMEGN PICVQ-IPWE K-NQDALAKW ANGQTGYPWI DAIMIQLREE GWIHHLSRHA VACFLTRGDL 353**

**DpCRY2_XP_032511492 286:NPNFDRMEGN PICVQ-IPWE K-NQEALKKW ANGQTGFPWI DAIMIQLRND GWIHHLARHA VACFLTRGDL 353**

**AgCRY2_ABB29887 286:NPTFDKMAGN PICVQ-IPWD R-NAEALAKW ASGQTGFPWI DAIMTQLREE GWIHHLARHA VACFLTRGDL 353**

**XtCRY4_NP_001123706 286:TPNFTHMVGN PICLQ-IDWY K-NEEQLQKW KEAKTGFPWI DAIMTQLHNE GWIHHLARHA VACFLTRGDL 353**

**XlCRY4_NP_001088990 286:TPNFTHMVGN PICLQ-IEWY K-NEEQLQKW REGKTGFPWI DAIMAQLHEE GWIHHLARHA VACFLTRGDL 353**

**cCRY4_NP_001034685 285:TPNFTKMAGN PICLQ-IRWY E-DAERLHKW KTAQTGFPWI DAIMTQLRQE GWIHHLARHA AACFLTRGDL 352**

**zCRY4_NP_571862 284:TPNFTKMEGN SICLQ-IDWY H-DPERLEKW RTAQTGFPWI DAIMTQLLQE GWIHHLARHA VACFLTRGDL 351**

**d(6-4)Phr_BAA12067 294:EPNFDRMLGN VYCMQ-IPWQ E-HPDHLEAW THGRTGYPFI DAIMRQLRQE GWIHHLARHA VACFLTRGDL 361**

**Dp(6-4)Phr_ABO38436 284:TENFDKMVGN SVCTQ-IPWK K-NDAHLKAW AEGKTGYPFV DAIMRQLKQE GWIHHLARHM VACFLTRGDL 351**

**Cg(6-4)Phr_XP_011414697 281:TPNFDKMEGN PVCKQ-IPWD T-NESYLKAW KEGRTGYPFI DAVMTQLRQE GWIHHLARHS VACFLTRGDL 348**

**Xt(6-4)Phr_LOC100144974 286:IPNFNKMEGN TVCVQ-VDWG N-NKEHLQAW SEGRTGYPFI DAIMTQLRTE GWIHHLARHA VACFLTRGDL 353**

**Xl(6-4)Phr_NP_001081421 286:IPNFNKMEGN PVCVQ-VDWD N-NKEHLEAW SEGRTGYPFI DAIMTQLRTE GWIHHLARHA VACFLTRGDL 353**

**z(6-4)Phr_NP_571863 286:IPNFNKMEGN SACVQ-VDWD N-NPEHLAAW REARTGFPFI DTIMTQLRQE GWIHHLARHA VACFLTRGDL 353**

**Tr(6-4)Phr_XP_011607950 285:ISNFNKMVDN PVCTQ-VDWD I-NSEYLAAW REARTGFPFI DAVMTQLRQQ GWIHHLARHA VACFLTRGDL 352**

**At(6-4)Phr_NP_566520 294:TPNFDKMKGN RICKQ-IPWN E-DHAMLAAW RDGKTGYPWI DAIMVQLLKW GWMHHLARHC VACFLTRGDL 361**

**CrCRY(animal-like)_XP_042923874 287:TPNFHRMAGN PVCKQ-IDWD D-NPEFLAAW REARTGFPWI DAIMTQLVTW GWMHHLARHS VACFLTRGDL 354**

**BmCRY1_NP_001182628 295:NPHYGQMTDN PICLD-IPWK SPEGDELERW ASGRTGFPFV DAAMRQLRLE GWLHHAVRNT VASFLTRGTL 363**

**DpCRY1_XP_032522602 295:NPNYAQMSGN PICLD-IPWK EPENDELQRW KEGRTGFPFV DAAMRQLRTE GWLHHVVRNT VASFLTRGTL 363**

**AgCRY1_ABB29886 306:NPHYGEMERN PICLN-IPWY KPEDDSLTRW KEGRTGFPMI DAAMRQLLAE GWLHHILRNI TATFLTRGGL 374**

**dCRY_NP_732407 307:NPNYDRMEGN DICLS-IPWA KPNENLLQSW RLGQTGFPLI DGAMRQLLAE GWLHHTLRNT VATFLTRGGL 375**

**CgCRY1_XP_034329330 301:NINYDKMETN PICLN-IPWY D-NPDHEEKW TQGETGYPWI DAIMKQLRYE GWVHHVARHA VSCFLTRGDL 368**

**XtCRY-dash_XP_031759840 288:YGRRIFFLRG LQDKD-VPWK K-DPKLFDAW KEGRTGVPFV DANMRELAMT GFMSNRGRQN VASFLTK-DL 354**

**XlCRY-dash_NP_001084438 288:YGRRIFFLRG LQDKD-IPWK R-DPKLFDAW KEGRTGVPFV DANMRELAMT GFMSNRGRQN VASFLTK-DL 354**

**zCRY-dash_NP_991249 288:YGNRIFYMNG LQDKH-VPWK T-DMKMFDAW KEGRTGVPFV DANMRELALT GFMSNRGRQN VASFLTK-DL 354**

**TrCRY-dash_XP_003968037 288:YGTKLFQVNG LQDKS-VSWR K-DMKLFNAW KEGKTGVPFV DANMRELATT GFMSNRGRQN VASFLTK-DL 354**

**XtCRY6_LC705158_(This_Study) 287:FPDMPSEPVR PAYKS-QRWS S-DLNHLRAW QKGLTGYPLV DAAMRELWLT GWMCNYSRHV VASFLVA-YL 353**

**XlCRY6_XP_018113364 287:FPDMPSEPVR PAYKS-QRWS S-DLNHLRAW QKGLTGYPLV DAAMRELWLT GWMCNYSRHV VASFLVA-YL 353**

**zCRY6_XP_009291670 287:FPDLPWESLR PPYKA-LRWS S-DHAHLKAW QRGRTGYPLV DAAMRQLWQT GWMNNYMRHV VASFLIA-YL 353**

**TrCRY6_XP_011605280 293:FPDLPWESLR PPYKA-LRWS T-DRRHLEAW QRGRTGYPLV DAAMRQLWLT GWMNNYMRHV VASFLIA-YL 359**

**CgCRY6_AQM57607 284:WPDLPSQPTR VHYRD-QAWS R-DAGHLKAW QRGRTGFPLV DAAMRQLWLE GWINNYLRHV VASFLIS-YL 350**

**CrCRY(plant-like)_AAC37438 285:FPFIHERSLL GHLRA-CPWR I-DQHAFKAW RQGQTGYPIV DAAMRQLWSS GWCHNRGRVV AASFLVK-DL 351**

**AtCRY1_NP_567341 282:HPYSHERPLL GHLKF-FPWA V-DENYFKAW RQGRTGYPLV DAGMRELWAT GWLHDRIRVV VSSFFVK-VL 348**

**AtCRY2_NP_171935 286:FPFTHEQSLL SHLRF-FPWD A-DVDKFKAW RQGRTGYPLV DAGMRELWAT GWMHNRIRVI VSSFAVK-FL 352**

**EcCPD_Phr_WP_001680736 274:YPSLCKHCPF IAWTDRVQWQ S-NPAHLQAW QEGKTGYPIV DAAMRQLNST GWMHNRLRMI SASFLVK-DL 341**

**(Figure S2: 7/8)**

**cCRY1_NP_989576 355:WISWEEGMKV FEELLLDADW SVNAGSWMWL SCSSF--FQQ FFHCYCPVGF GRRTDPNGDY IRRYLPVLRG 422**

**XtCRY1_NP_001017311 355:WISWEEGMKV FEELLLDADW SVNAGSWMWL SCSSF--FQQ FFHCYCPVGF GRRTDPNGDY IRRYLPILKG 422**

**XlCRY1_NP_001081129 355:WISWEEGMKV FEELLLDADW SVNAGSWMWL SCSSF--FQQ FFHCYCPVGF GKRTDPNGDY IRRYLPILKG 422**

**mCRY1_NP_031797 355:WISWEEGMKV FEELLLDADW SINAGSWMWL SCSSF--FQQ FFHCYCPVGF GRRTDPNGDY IRRYLPVLRG 422**

**zCRY1aa_NP_001070765 355:WISWEEGMKV FEELLLDADW SVNAGSWMWL SCSSF--FQQ FFHCYCPVSF GRRTDPNGDY IRRYLPVLRG 422**

**zCRY1ab_BAA96847 355:WISWEEGMKV FEELLLDADW SVNAGSWMWL SCSSF--FQQ FFHCYCPVGF GRRTDPNGDY IRRYLPILRG 422**

**TrCRY1_XP_011611545 355:WIGWEEGMKV FEELLLDADW SVNAGSWMWL SCSSF--FQQ FFHCYCPVGF GRRTDPNGDY IRRYLPILRG 422**

**zCRY1ba_BAA96848 355:WISWEEGMKV FEELLLDADW SVNAGSWMWL SCSSF--FQQ FFHCYCPVGF GRRTDPNGDF IRRYLPILRG 422**

**TrCRY2_XP_011617050 355:WISWEEGMKV FDELLLDADW SVNAGSWMWL SCSSF--FQQ FFHCYCPVGF GRRTDPNGDF IRRYLPILRG 422**

**zCRY1bb_NP_571867 358:WISWEEGMKV FEELLLDADW SVNAGSWLCH SCSSF--FQQ FFHCYCPVGF GRRIDPNGDF IRRYLPVLRD 425**

**cCRY2_NP_989575 355:WISWESGVRV FDELLLDADF SVNAGSWMWL SCSAF--FQQ FFHCYCPVGF GRRTDPSGDY VKRYLPKLKG 422**

**mCRY2_NP_034093 355:WVSWESGVRV FDELLLDADF SVNAGSWMWL SCSAF--FQQ FFHCYCPVGF GRRTDPSGDY IRRYLPKLKG 422**

**XtCRY2_XP_031756344 355:WNSWECGVKV FDELLLDADF SVNAGSWMWL SCSAF--FQQ FFHCYCPVGF GRRTDPSGDY VRRYLPVLKA 422**

**XlCRY2_AAK94667 355:WNSWECGVKV FDELLLDADF SVNAGSWMWL SCSAF--FQQ FFHCYCPVGF GRRTDPSGDY VKRYLPVLKA 422**

**zCRY2(zCRY3)_BAA96850 355:WISWESGMKV FEELLLDADW SVNAGSWMWL SCSAF--FQQ FFHCYCPVGF GRRTDPSGDY IRRYIPKLKD 422**

**TrCRY3_XP_029687911 355:WISWECGMKV FEELLLDADW SVNAGSWMWL SCSAF--FQQ FFKCYCPVGF GRRTDPSGDY IRRYIPILKD 422**

**BmCRY2_NP_001182627 354:WISWEEGMKV FDELLLDADW SVNAGMWMWF SCSSF--FQQ FFHCYCPVRF GRKTDPNGDF IRKYIPALKN 421**

**DpCRY2_XP_032511492 354:WISWEEGMKV FDELLLDADW SVNAGMWMWL SCSSF--FQQ FFHCYCPVRF GRKTDPNGDF IRKYIPVLKN 421**

**AgCRY2_ABB29887 354:WISWEEGMKV FEELLLDADW SVNAGMWMWL SCSSF--FQQ FFHCYCPVKF GRKADPNGDY IRRYLPVLKN 421**

**XtCRY4_NP_001123706 354:WISWEEGMKV FEEFLLDADY CINAGNWMWL SASAF--FHH YTRIFCPVRF GKRSDPEGNY IRKYLPVLKN 421**

**XlCRY4_NP_001088990 354:WISWEEGMKV FEELLLDADY SINAGNWMWL SASAF--FHH YTRIFCPVRF GRRTDPEGNY IRKYLPVLKN 421**

**cCRY4_NP_001034685 353:WISWEEGMKV FEELLLDADY SINAGNWMWL SASAF--FHH YTRIFCPVRF GRRTDPEGQY IRKYLPILKN 420**

**zCRY4_NP_571862 352:WISWEEGMKV FEEFLLDADY SVNAGNWMWL SASAF--FHK YTRIFCPVRF GRRTDPQGEY LRKYLPVLKN 419**

**d(6-4)Phr_BAA12067 362:WISWEEGQRV FEQLLLDQDW ALNAGNWMWL SASAF--FHQ YFRVYSPVAF GKKTDPQGHY IRKYVPELSK 429**

**Dp(6-4)Phr_ABO38436 352:WISWEEGAKV FEDFLLDYDW SLNAGNWMWL SASAF--FYK YYRVYSPVAF GKKTDKDGLY IRKYVPELKK 419**

**Cg(6-4)Phr_XP_011414697 349:WINWEEGMKV FEEYLLDADW SLNAGNWMWL SASAF--FHQ YFRVYSPIEF GKKTDKDGDY IRKYVPQLSK 416**

**Xt(6-4)Phr_LOC100144974 354:WISWEEGQKV FEELLLDADW SLNAGNWQWL SASTF--FHQ FFRVYSPVAF GKKTDKNGDY IKKYLPILKK 421**

**Xl(6-4)Phr_NP_001081421 354:WISWEEGQKV FEELLLDADW SLNAGNWLWL SASAF--FHQ FFRVYSPVAF GKKTDKNGDY IKKYLPILKK 421**

**z(6-4)Phr_NP_571863 354:WISWEEGQKV FEELLLDSDW SLNAGNWQWL SASTF--FHQ YFRVYSPIAF GKKTDKHGDY IKKYLPVLKK 421**

**Tr(6-4)Phr_XP_011607950 353:WISWEEGQKV FEELLLDGDW ALNAGNWQWL SASAF--FHQ FFRVYSPVAF GKKTDKNGDY IKKFLPHLKK 420**

**At(6-4)Phr_NP_566520 362:FIHWEQGRDV FERLLIDSDW AINNGNWMWL SCSSF--FYQ FNRIYSPISF GKKYDPDGKY IRHFLPVLKD 429**

**CrCRY(animal-like)_XP_042923874 355:YVSWERGMEV FEEHLIDQDH YLNAANWMWL SASAF--FSQ YFRVYSPVVF GKKYDPEGRF IRKFLPVLKD 422**

**BmCRY1_NP_001182628 364:WLSWEHGLAH FLKYLLDADW SVCAGNWMWV SSSAFEALLD SGECACPVRL GQRLDPSGEY VRRYVPELAR 433**

**DpCRY1_XP_032522602 364:WLSWEHGLQH FLKYLLDADW SVCAGNWMWV SSSAFEALLD SGECACPVRL GRRLEPTGHY VRRYVPELAR 433**

**AgCRY1_ABB29886 375:WLSWEEGLQH FLKYLLDADW SVCAGNWMWV SSSAFERLLD SSKCTCPIAL ARRLDPKGDY VKRYLPELAN 444**

**dCRY_NP_732407 376:WQSWEHGLQH FLKYLLDADW SVCAGNWMWV SSSAFERLLD SSLVTCPVAL AKRLDPDGTY IKQYVPELMN 445**

**CgCRY1_XP_034329330 369:WLNWEVGLKV FYKYLLDADW SVCAGNWMWV SSSAFEKVLQ CPNCFCPVRY GKRMDPSGEY VRRYLPVLKD 438**

**XtCRY-dash_XP_031759840 355:XIDWRLGAEW FEYLLVDYDV CSNYGNWLYS AGIGND--PR ENRKFNMIKQ GLDYDAGGDY IRLWVPELQQ 422**

**XlCRY-dash_NP_001084438 355:GIDWRMGAEW FEYLLVDYDV CSNYGNWLYS AGIGND--PR ENRKFNMIKQ GLDYDSGGDY IRLWVPELQQ 422**

**zCRY-dash_NP_991249 355:GLDWRLGAEW FEYLLVDHDV CSNYGNWLYS AGIGND--PR ENRKFNMIKQ GLDYDNNGDY VRQWVPELRG 422**

**TrCRY-dash_XP_003968037 355:GLDWRMGAEW FEYLLVDHDV CSNYGNWLYS AGIGND--PR ENRKFNMIKQ GLDYDNNGEY VRLWVPELQR 422**

**XtCRY6_LC705158_(This_Study) 354:HIHWVHGYRW FQDTLLDADV AINAMMWQNG GMSGLD---H WNFVMHPVDS ALTCDPYGSY VRKWCPELAG 420**

**XlCRY6_XP_018113364 354:HIHWVHGYRW FQDTLLDADV AINAMMWQNG GMSGLD---H WNFVMHPVDS ALTCDPYGSY VRKWCPELAG 420**

**zCRY6_XP_009291670 354:HFPWQEGYRW FQDTLVDADV AIDAMMWQNG GMCGLD---H WNFVMHPIDA ALTCDPCGTF VRQWCPELKA 420**

**TrCRY6_XP_011605280 360:HLPWQEGYRW FQDTLVDADV AIDAMMWQNG GMCGLD---H WNFVMHPVDA AMTCDPCGQY VRKWCPELSG 426**

**CgCRY6_AQM57607 351:RLHWVEGYRW FQDTLLDADV AINAMMWQNG GMSGLD---Q WNFVMHPVDA ALTCDPDGAY VRKWCPEIAA 417**

**CrCRY(plant-like)_AAC37438 352:LLPWQWGLKH YWDAQIDADL ECDALGWQYV SGGMSD-AHP FSYMMDLEKE ARRFDPDGEY VRRWLPALSR 420**

**AtCRY1_NP_567341 349:QLPWRWGMKY FWDTLLDADL ESDALGWQYI TGTLPD-SRE FDRIDNPQFE GYKFDPNGEY VRRWLPELSR 417**

**AtCRY2_NP_171935 353:LLPWKWGMKY FWDTLLDADL ECDILGWQYI SGSIPD-GHE LDRLDNPALQ GAKYDPEGEY IRQWLPELAR 421**

**EcCPD_Phr_WP_001680736 342:LIDWREGERY FMSQLIDGDL AANNGGWQWA ASTGTD-AAP YFRIFNPTTQ GEKFDREGEF IRRWLPELRD 410**

**(Figure S2: 8/8)**

**cCRY1_NP_989576 423:FPAKYIYDPW NAPESVQKAA KCVIGVNY 450**

**XtCRY1_NP_001017311 423:FPPKYIYDPW NAPETVQKVA KCIIGVNY 450**

**XlCRY1_NP_001081129 423:FPPKYIYDPW NAPETVQKAA KCIIGVNY 450**

**mCRY1_NP_031797 423:FPAKYIYDPW NAPEGIQKVA KCLIGVNY 450**

**zCRY1aa_NP_001070765 423:FPAKYIYDPW NAPESVQKAA KCIIGVHY 450**

**zCRY1ab_BAA96847 423:FPAKFIYDPW NAPESVQKVA KCIIGVHY 450**

**TrCRY1_XP_011611545 423:FPAKYIYDPW NAPESVQKAA KCMIGVHY 450**

**zCRY1ba_BAA96848 423:FPAKYIYDPW NAPDSVQAAA KCIIGVHY 450**

**TrCRY2_XP_011617050 423:FPAKYIYDPW NAPEAVQAAA KCVIGVHY 450**

**zCRY1bb_NP_571867 426:FPAKYIYDPW NAPHDVQLAA KCVIGVDY 453**

**cCRY2_NP_989575 423:FPSRYIYEPW NAPESVQKAA KCIIGVDY 450**

**mCRY2_NP_034093 423:FPSRYIYEPW NAPESVQKAA KCIIGVDY 450**

**XtCRY2_XP_031756344 423:FPSRYIYEPW SAPESVQKEA KCIIGIDY 450**

**XlCRY2_AAK94667 423:FPSRYIYEPW SAPESVQKEA KCIIGIDY 450**

**zCRY2(zCRY3)_BAA96850 423:YPNRYIYEPW NAPESVQKAA NCIVGVDY 450**

**TrCRY3_XP_029687911 423:YPNRYIYEPW NAPEAVQKAA NCVVGVDY 450**

**BmCRY2_NP_001182627 422:MPTRYIHEPW MAPESVQAAA QCSIGRDY 449**

**DpCRY2_XP_032511492 422:MPTRYIHEPW VCPEEIQKSI RCIIGKDY 449**

**AgCRY2_ABB29887 422:FPTRFIHEPW NASESVQRAA KCLIGKDY 449**

**XtCRY4_NP_001123706 422:FPAKYIYEPW TAPEEIQKQA GCLIGKDY 449**

**XlCRY4_NP_001088990 422:FPAKYIYAPW TAPEEIQKQS GCLIGKDY 449**

**cCRY4_NP_001034685 421:FPSKYIYEPW TASEEEQKQA GCIIGRDY 448**

**zCRY4_NP_571862 420:FPSQYIYEPW KAPEDVQLSA GCIIGKDY 447**

**d(6-4)Phr_BAA12067 430:YPATCIYEPW KASLVDQRAY GCVLGTDY 457**

**Dp(6-4)Phr_ABO38436 420:YPSEFIYEPW KAPKGVQKTA GCIIGEGY 447**

**Cg(6-4)Phr_XP_011414697 417:YPTAYIYEPW KAPLKVQEKA GCIVGKDY 444**

**Xt(6-4)Phr_LOC100144974 422:FPAEYIYEPW KAPRSLQERA GCIIGKDY 449**

**Xl(6-4)Phr_NP_001081421 422:FPAEYIYEPW KSPRSLQERA GCIIGKDY 449**

**z(6-4)Phr_NP_571863 422:FPTEYIYEPW KAPRSVQERA GCIVGKDY 449**

**Tr(6-4)Phr_XP_011607950 421:FPAEYIFEPW KAPQSVQQAA GCIVGKDY 448**

**At(6-4)Phr_NP_566520 430:MPKQYIYEPW TAPLSVQTKA NCIVGKDY 457**

**CrCRY(animal-like)_XP_042923874 423:MPAKYIYEPW TAPLEVQRKA GCVVGRDY 450**

**BmCRY1_NP_001182628 434:VPTEYIYEPW KAPLDVQERA NCIIGKDY 461**

**DpCRY1_XP_032522602 434:MPGEYIYEPW RAPLEVQEAA GCVIGRDY 461**

**AgCRY1_ABB29886 445:YPAQFVHEPW KASREQQIEY GCVIGEKY 472**

**dCRY_NP_732407 446:VPKEFVHEPW RMSAEQQEQY ECLIGVHY 473**

**CgCRY1_XP_034329330 439:MPLRYLFEPW KAPLPVQQKA KCIVGVDY 466**

**XtCRY-dash_XP_031759840 423:IKGGDAHTPW ALSTASLAHS NVSLGETY 450**

**XlCRY-dash_NP_001084438 423:IKGGDAHTPW ALSNASLAHA NLSLGETY 450**

**zCRY-dash_NP_991249 423:IKGGDVHTPW TLSNSALSHA QVSLNQTY 450**

**TrCRY-dash_XP_003968037 423:IMGADVHTPW TLSSAILSHA HLSLGETY 450**

**XtCRY6_LC705158_(This_Study) 421:LPDEYIHKPW KCAPSQLRRA GVILGRNY 448**

**XlCRY6_XP_018113364 421:LPDEYIHKPW KCAPSQLRRA GVILGQNY 448**

**zCRY6_XP_009291670 421:LPDDLIHKPW KCPTSMLRRA GVVFGDSY 448**

**TrCRY6_XP_011605280 427:LPDELIHKPW RCPTSLLRRA GVVFGQTY 454**

**CgCRY6_AQM57607 418:LPNDFIHQPW KCPPSILRRC GIKLGETY 445**

**CrCRY(plant-like)_AAC37438 421:LPTEYIHAPW KAPASVLAAA DVELGCNY 448**

**AtCRY1_NP_567341 418:LPTDWIHHPW NAPESVLQAA GIELGSNY 445**

**AtCRY2_NP_171935 422:LPTEWIHHPW DAPLTVLKAS GVELGTNY 449**

**EcCPD_Phr_WP_001680736 411:VPGKAVHEPW KWA----QKA GVML--DY 432**

**Figure S2 Alignment of CRYs for phylogenetic analysis by MAFFT (https://mafft.cbrc.jp/alignment/server/)**

Amino- and carboxyl-terminal sequences (corresponding to Met^1^-His^243^ and Pro^692^-Asn^862^) were deleted to construct the phylogenetic tree (Figure 1a).

**(Figure S3: 1/19)**

**EKC36382.1_Crassostrea_gigas 1:---------- ---------- ---------- ---------- ----MIVSLD GYDSTRQHFL 16**

**ENSLLEP00000020779_Leptobrachium_leishanense 1:--------MA GAEG-----A ALTHMEGFMQ EMLTGSIQPD EFFCLVLSLL GPNRTHTLLP 47**

**XtCRY6_LC705158 (This_Study) 1:--------MD LKP------F ERAQIDDVLQ QLESGSVQAD EFLCLVLSIL GSSRTYSQFP 46**

**ENSXLAT00005054664_Xenopus_laevis 1:--------MD LEA------V ERAHINDIVR QLETGIVQTD EFLCLVLSVL GNRRTFWHLP 46**

**ENSLOCP00000004832_Lepisosteus_oculatus 1:---------- ---------- ----VQQMMA ELQLGELDTE EFFCLTLSLL GHQNTQEQFL 36**

**ENSSFOP00015030596_Scleropages_formosus 1:--------MP ATDSS-RPAA HVSRVQAMVT QVLLGTMEPE ELFWGSLSLL GLQGTQDTFL 51**

**ENSCHAP00000044642_Clupea_harengus 1:--------MP VEND-----S SVAQVRHLLR ELLVGREDPG GFFCLCLSIL GSRETRAGFR 47**

**ENSAMXP00000003765_Astyanax_mexicanus 1:--------MS V--------S AMSEVRLKLR DLLLGLEDAE GFFCLCLSAL GERDTLALFL 44**

**ENSDARP00000105169_Danio_rerio 1:--------MS AGEE----TK PVSEVRELLR ELILGREDPQ GFFCMCVSLL GDADTRRLFL 48**

**ENSSGRP00000063675_Sinocyclocheilus_grahami 1:--------MS VRRD----MK SMSQVRELLR ELESGREDPD GFFGLCLSLL GDTDTRTHFL 48**

**ENSCARP00000075848_Carassius_auratus/1801 1:--------MS VSRD----MK SVSQVRELLR ELVSGREDPD GFFCMCLSLL GDTDTRTHFL 48**

**ENSCARP00000074480_Carassius_auratus/1814 1:--------MS VSRD----MK SVSQVRELLR ELVSGREDPD AFFCMCLSLL GDTDTRTHFL 48**

**ENSGMOP00000042713_Gadus_morhua 1:--------MN VKRTIAGAAD NIASLSNTLQ QLVLGGEDPG GFFGICVSSM GHSETLSVFP 52**

**ENSNFUP00015037037_Nothobranchius_furzeri 1:--------MP SVA------D DLALVRHMLR EVLVGREDPE GYFAMCLSIL GHQETLSQFA 46**

**ENSTRUP00000037131_Takifugu_rubripes 1:--------MP PSAAP-DGDS TKVWVRRILR EVLAGREDPE GFFALCLSVL GHQETRSQYP 51**

**ENSKMAP00000018790_Kryptolebias_marmoratus 1:--------MA LSAA-----G DQAEVRKMLR EVLVGREDPE GFFAVCLSVL GHQETRTQFL 47**

**ENSCVAP00000003000_Cyprinodon_variegatus 1:---------- ---------- ---------- ---------- ----MCLSVL GNQETRSEFL 16**

**ENSPFOP00000018725_Poecilia_formosa 1:--------MP HSAA-----G DQAAVRQMLR EVLVGREDPE GFFAMCVSVL GHQETRSQFL 47**

**ENSFHEP00000001658_Fundulus_heteroclitus 1:--------MP PSAA-----G DQAAVRQMLR EVLVGREDPD SFFAMCVSVL GHQETRSQFL 47**

**ENSOMYP00000024389_Oncorhynchus_mykiss 0:---------- ---------- ---------- ---------- ---------- ---------- 0**

**ENSOTSP00005065052_Oncorhynchus_tshawytscha 1:--------MP VSGG----ED PMSQVRQMLR ELLVGRENAE GFFCLCVSVL GHNDTRTHFL 48**

**ENSOKIP00005070961_Oncorhynchus_kisutch 1:--------MP VSGG----ED PMSQVRQMLR ELLVGRENAE GFFCLCVSVL GHNDTRTHFL 48**

**ENSHHUP00000077840_Hucho_hucho 1:--------MP VSGG----ED PMSQVRQMLR ELLVGRENAE GFFCLCVSVL GHNDTRTHFL 48**

**ENSSSAP00000016302_Salmo_salar 1:--------MP VSGG----ED PMSQVRQMLR ELLVGRENAE GFFCLCVSVL GHNDTRTHFL 48**

**ENSSTUP00000081428_Salmo_trutta 1:--------MP VSGG----ED PMSQVRQMLR ELLVGRENAE GFFCLCVSVL GHNDTRTNFL 48**

**ENSHCOP00000008781_Hippocampus_comes 1:---------- ---------- ---------- ---------- ----MCLSIL GHQETRARFL 16**

**ENSOJAP00000007613_Oryzias_javanicus 1:--------MP PSKG------ DHALVREMLK EVLTGREDPE GFFAICLSVL GHRETYSMFP 46**

**ENSORLP00000011698_Oryzias_latipes 1:--------MP PSKS------ DQALVSEMLK EVLAGREDPE GFFAICLSVL GHRETYSVFQ 46**

**ENSOSIP00000024525_Oryzias_sinensis 1:--------MP PSKS------ DQDLVREMLK EVLAGREDPE GFFAICLSVL GHRETYSVFQ 46**

**ENSBSLP00000028581_Betta_splendens 1:--------MP RSAAA--IDD SKAWVRKMIR EVLVGREDPE GLFAMCLSVL GPQETRSQFL 50**

**ENSATEP00000003870_Anabas_testudineus 1:--------MP LSAAG--GGD SEVLVRKILR EVLLGREDPE GFFAMCLSVL GHQETRSQFL 50**

**ENSMMDP00005031819_Myripristis_murdjan 1:--------MP PAAG---VDD TKVLVRKMLR ELLMGREDPE GFFGLCVSLL GHQDTRSQFP 49**

**ENSONIP00000067431_Oreochromis_niloticus 1:--------MP PSGA-----D AQATVKWWLG EVLEGREDPE GFFAMCVSIL GHRETRSQFL 47**

**ENSNBRP00000029628_Neolamprologus_brichardi 1:--------MP PSGA-----D AQATVKWWLG EVLKGREDPE GFFAMCLSIL GHVETRSQFL 47**

**ENSHBUP00000030531_Haplochromis_burtoni 1:---------- ---------- ---------- ---------- ----MCVSIL GQVETRSQFL 16**

**ENSPNYP00000010231_Pundamilia_nyererei 1:MSVTFYSKMP PSGA-----D AQATVKWWLG EVLEGREDPE GFFAMCVSIL GHLETRSQFL 55**

**ENSMZEP00005026771_Maylandia_zebra 1:MSVTFYSKMP PSGA-----D AQATVKWWLG EVLEGREDPE GFFAMCVSIL GQVETRSQFL 55**

**ENSACLP00000035664_Astatotilapia_calliptera 1:MSVTFYSKMP PSGA-----D AQATVKWWLG EVLEGREDPE GFFAVCVSIL GQVETRSQFL 55**

**ENSLBEP00000024994_Labrus_bergylta 0:---------- ---------- ---------- ---------- ---------- ---------- 0**

**ENSCLMP00005004807_Cyclopterus_lumpus 1:--------MP PSAG------ -KVLVRKTLR EVLMGREDPE GFFATCVSAL GHQETRSQFR 45**

**ENSCGOP00000000746_Cottoperca_gobio 1:--------MP PSAAA--TED SIALVRKTLR EVLVGREDPE GFFATCVSAL GHQETRSHFL 50**

**ENSSPAP00000013334_Stegastes_partitus 1:---------- ---------- ---------- ---------- ----MCVSVL GHQETRTQFP 16**

**ENSAPOP00000010991_Acanthochromis_polyacanthus 1:--------MP PPAA-----D GQTEVRKMLR DVLVGREDPE GFFAMCVSIL GHRETRTLFP 47**

**ENSAPEP00000019384_Amphiprion_percula 1:--------MP PPAA-----D GQTEVRKMLR EVLVGREDPE GFFAMCVSIL GHRETRTVFP 47**

**ENSAOCP00000003852_Amphiprion_ocellaris 1:---------- ---------- ---------- ---------- ----MCVSIL GHRETRTVFP 16**

**ENSLCRP00005003692_Larimichthys_crocea 1:--------MS PSAAA-GAGD SKALVRRMLR EVLVGREDPE GFFVMCLSAL GHQETRSQFV 51**

**ENSSMAP00000005463_Scophthalmus_maximus 1:--------MP RPVVD--ADD SKASVRKWLR EVLAGREDPE GFFAMCVSVL GHQETRSQFQ 50**

**ENSSLDP00000025094_Seriola_lalandi_dorsalis 1:--------MP PSAGG--AED SKTLVRKLLR EVLVGREDPE GFFAMCLSVL GHQETRSQFP 50**

**ENSLCAP00010022815_Lates_calcarifer 1:--------MS PSAVG--ADD SKSLVRKMLR EVLVGREDPE GFFAMCVSLL GHQETRSRFL 50**

**ENSSAUP00010027448_Sparus_aurata 1:--------MP PAAAA-GAED SKALVRKMLR EVLVGREDPE WFFAMCVSAL GHQETRSQFL 51**

**ENSDLAP00005056549_Dicentrarchus_labrax 1:--------MP PSTG---AED RKALVRKMLR EVLVGREDPE GFFAMCVSAL GYQETRSQFL 49**

**(Figure S3: 2/19)**

**EKC36382.1_Crassostrea_gigas 17:KNIDFLRLTN PKKYNELFTV FANYFE---- ---K------ -----PPSGA FLDPIGCSVE 58**

**ENSLLEP00000020779_Leptobrachium_leishanense 48:GIITGLREQN PVLYSQIMHI HSEYFH---- ---K------ -----DVEE- ---------- 78**

**XtCRY6_LC705158 (This_Study) 47:AILQSLSRKE PAMYRELMDL HAEYFP---- ---K------ -----EPAD- ---------- 77**

**ENSXLAT00005054664_Xenopus_laevis 47:AIIQSLREKE PAMYRELMDL HAHYFR---- ---K------ -----EPTD- ---------- 77**

**ENSLOCP00000004832_Lepisosteus_oculatus 37:ELIEPLALRH EKEHMCLTTI FLEYFT---- ---QAQ---- --APGVEEE- ---------- 72**

**ENSSFOP00015030596_Scleropages_formosus 52:DLIEPLAQEH ALSQDQ---- ---------- --EA------ -----KEDE- ---------- 73**

**ENSCHAP00000044642_Clupea_harengus 48:ELIAPLTDKH RQLHSQLITI YQEYFS---- ---REEDDEL RLAPQDEDD- ---------- 89**

**ENSAMXP00000003765_Astyanax_mexicanus 45:DLIKPLASGH VQLYSQLSAI HTNYFS---- ---R------ -----DEEE- ---------- 7**

**ENSDARP00000105169_Danio_rerio 49:DLIKPLSSEY EHLHSQLTSV FLEYFS---- ---K------ -----DESE- ---------- 79**

**ENSSGRP00000063675_Sinocyclocheilus_grahami 49:DVIKPLLSGH EHIHSQLTAI FLEYFS---- ---K------ -----DEAD- ---------- 79**

**ENSCARP00000075848_Carassius_auratus/1801 49:DEIKPLLSGH EHLHSQLTAI FLEYFS---- ---K------ ---------- ---------- 75**

**ENSCARP00000074480_Carassius_auratus/1814 49:DEIKPLLSGH EHLHSQLTAI FLEYFS---- ---K------ -----DKAD- ---------- 79**

**ENSGMOP00000042713_Gadus_morhua 53:SLIQPLASVD PSQHSTLIAI YVEYFS---- ---K------ -----DEED- ---------- 83**

**ENSNFUP00015037037_Nothobranchius_furzeri 47:SIIKPLSTAN IHLHSILTSI YQEYFS---- ---K------ -----TEDD- ---------- 77**

**ENSTRUP00000037131_Takifugu_rubripes 52:SHIEPLRAAN SSMHSVLTSI YKEYFS---- ---K------ -----TEDD- ---------- 82**

**ENSKMAP00000018790_Kryptolebias_marmoratus 48:SIVKPLSTAN IHLHSVLTSI YREYYS---- ---K------ -----TEDE- ---------- 78**

**ENSCVAP00000003000_Cyprinodon_variegatus 17:SIIKPLSTAN IRLHSVLRSL YQDYFT---- ---K------ -----TEDD- ---------- 47**

**ENSPFOP00000018725_Poecilia_formosa 48:SIIKPLSTAN IRLHSVLSSI YQEYFS---- ---K------ -----MEDD- ---------- 78**

**ENSFHEP00000001658_Fundulus_heteroclitus 48:AIIKPLSTAN IRLHSVLSAL YQQYFS---- ---K------ -----PEDD- ---------- 78**

**ENSOMYP00000024389_Oncorhynchus_mykiss 0:---------- ---------- ---------- ---------- ---------- ---------- 0**

**ENSOTSP00005065052_Oncorhynchus_tshawytscha 49:PLIQLLATDH NRLHTTLTSI YLEYFS---- ---K------ -----DEDD- ---------- 79**

**ENSOKIP00005070961_Oncorhynchus_kisutch 49:PLIQLLATDH NRLHTTLTSI YLEYFS---- ---K------ -----DEDD- ---------- 79**

**ENSHHUP00000077840_Hucho_hucho 49:PLIQLLATDH NSLHTTLTSI YLKYFS---- ---K------ -----DEDD- ---------- 79**

**ENSSSAP00000016302_Salmo_salar 49:PLIQLLATDH SSLHTTLTSI YLEYFS---- ---K------ -----DEDD- ---------- 79**

**ENSSTUP00000081428_Salmo_trutta 49:PLIQLLATDH SSLHTTLTSI YLEYFS---- ---K------ -----DEDD- ---------- 79**

**ENSHCOP00000008781_Hippocampus_comes 17:PAVQPLSSAN RFLHSTLVSI YEEYFT---- ---K------ -----AEDD- ---------- 47**

**ENSOJAP00000007613_Oryzias_javanicus 47:ALIKPLSTAN ISVHSTLTLI YEEYFS---- ---K------ -----TGDD- ---------- 77**

**ENSORLP00000011698_Oryzias_latipes 47:TLIKPLSTAN TSVHSTLTLI YEEYFS---- ---K------ -----TEDD- ---------- 77**

**ENSOSIP00000024525_Oryzias_sinensis 47:TLIKPLSTAN TSVHSTLTLI YEQYFS---- ---K------ -----TADD- ---------- 77**

**ENSBSLP00000028581_Betta_splendens 51:CLIRPLAAAN AELHFRLSSI YQEYFS---- ---KFQ---- -----TEED- ---------- 83**

**ENSATEP00000003870_Anabas_testudineus 51:SLIHPLSTAN SSLHSTLTSI YQEFFT---- ---Q------ -----TEDD- ---------- 81**

**ENSMMDP00005031819_Myripristis_murdjan 50:ALIQPLSTAN RSLHSTLTSI YLDYFS---- ---Q------ -----DEDH- ---------- 80**

**ENSONIP00000067431_Oreochromis_niloticus 48:SLIEPLSTGD RLLHASLTSI YQEYFT---- ---K------ -----TEDD- ---------- 78**

**ENSNBRP00000029628_Neolamprologus_brichardi 48:SLIKPLSTGD RLLHASLTSI YQEYFT---- ---K------ -----TEDD- ---------- 78**

**ENSHBUP00000030531_Haplochromis_burtoni 17:SLIEPLSTGD RLLHTSLTSI YQEYFT---- ---K------ -----TEDD- ---------- 47**

**ENSPNYP00000010231_Pundamilia_nyererei 56:SLIEPLSTGD RLLHTSLTSI YQEYFT---- ---K------ -----TEDD- ---------- 86**

**ENSMZEP00005026771_Maylandia_zebra 56:SLIEPLSTGD RLLHASLTSI YQEYFT---- ---K------ -----TEDD- ---------- 86**

**ENSACLP00000035664_Astatotilapia_calliptera 56:SLIEPLSTGD RLLHASLTSI YQEYFT---- ---K------ -----TEDD- ---------- 86**

**ENSLBEP00000024994_Labrus_bergylta 1:--------MG CKLAYQIFYL YFNYKQ---- ---F------ -----FNND- ---------- 23**

**ENSCLMP00005004807_Cyclopterus_lumpus 46:SLIRPLCTAH GPLHSTLTSI YEQYFT---- ---E------ -----TEDD- ---------- 76**

**ENSCGOP00000000746_Cottoperca_gobio 51:SLIQPLSTAN SSLHSRLTSI YQEYFS---- ---K------ -----TEDD- ---------- 81**

**ENSSPAP00000013334_Stegastes_partitus 17:SLIQPLSTAN RALHSILTSI YQEYFS---- ---K------ -----TEDD- ---------- 47**

**ENSAPOP00000010991_Acanthochromis_polyacanthus 48:SLIQPLSTAN RALHSILISI YQEYFT---- ---K------ -----TEDD- ---------- 78**

**ENSAPEP00000019384_Amphiprion_percula 48:SLIQPLSTAN RALHSILISI YQEYFT---- ---K------ -----TEDD- ---------- 78**

**ENSAOCP00000003852_Amphiprion_ocellaris 17:SLIQPLSTAN RALHSILISI YQEYFT---- ---K------ -----TEDD- ---------- 47**

**ENSLCRP00005003692_Larimichthys_crocea 52:SLIQPLSTAN SSLHCTLTSI YREYFS---- ---K------ -----TEDD- ---------- 82**

**ENSSMAP00000005463_Scophthalmus_maximus 51:SLIQPLSAAN RSLHAALTSV YQGYFH---- ---K------ -----TEDD- ---------- 81**

**ENSSLDP00000025094_Seriola_lalandi_dorsalis 51:SLIRPLSTAN CSMYSTLTGI YQEYFSVVSE CLLN------ -----TEDD- ---------- 88**

**ENSLCAP00010022815_Lates_calcarifer 51:SLIQPLSTAN SSLYSTLTSI YKKYFS---- ---E------ -----TEDD- ---------- 81**

**ENSSAUP00010027448_Sparus_aurata 52:SLIQPLSTAN SSLHSTLTSI YREYFS---- ---K------ -----TEDD- ---------- 82**

**ENSDLAP00005056549_Dicentrarchus_labrax 50:SLIQPLSTAN RSLHSTLTSI YKDYFS---- ---K------ -----TEDD- ---------- 80**

**(Figure S3: 3/19)**

**DI-UIM**

**EKC36382.1_Crassostrea_gigas 59:NGYLTSDFGY ELEVELASAL SLQDQSESL- NSKA------ ---------S YVSVVQ-EPI 101**

**ENSLLEP00000020779_Leptobrachium_leishanense 79:------ALDC DVGLELAIAL SLHGNTSPE- QTAEVCPAMP ---------V QEQLRK-TTN 121**

**XtCRY6_LC705158 (This_Study) 78:----LETLGY ETDLELAIAL SLQEHNQLT- DTASF----- ---------- --ASEV-DPA 114**

**ENSXLAT00005054664_Xenopus_laevis 78:----L----- ETDLELAIAL SMHEQNQLT- DTAS------ ---------- ---SEM-SPT 107**

**ENSLOCP00000004832_Lepisosteus_oculatus 73:---------- --EVEVALAL SLQELGVSK- QEKP------ ---------Q PSS----HPG 100**

**ENSSFOP00015030596_Scleropages_formosus 74:---------- --ALALALAL SLQDAGAGK- QMTT------ ---------E ESSGSR-SQD 104**

**ENSCHAP00000044642_Clupea_harengus 90:---------- --ELQLALAL SLVHTSCQN- ---------- ---------- ---SPR-QPV 112**

**ENSAMXP00000003765_Astyanax_mexicanus 76:---------- --ALEVAIAL SLHDTNQRS- ---------- ---------- ---------- 92**

**ENSDARP00000105169_Danio_rerio 80:---------- --ELELALTL SLYETKQID- ------P--- ED-------K LHNSDH-QHH 109**

**ENSSGRP00000063675_Sinocyclocheilus_grahami 80:---------- --ELELALTQ SLYETKLTK- ------P--- ---------- ----DH-QP- 101**

**ENSCARP00000075848_Carassius_auratus/1801 76:---------- -----LALAL SLYETKLTK- ------P--- ---------- ----VH-QP- 94**

**ENSCARP00000074480_Carassius_auratus/1814 80:---------- --ELELALAL SLYETKLTK- ------P--- ---------- ----VH-QP- 101**

**ENSGMOP00000042713_Gadus_morhua 84:---------- --ELEVALAL SLLDVKPQQ- KPACR-P--- ---------- -------EPR 109**

**ENSNFUP00015037037_Nothobranchius_furzeri 78:---------- --EMELALAL SLLQMKDQQ- MATS------ ---------N NEPTPL-QFG 108**

**ENSTRUP00000037131_Takifugu_rubripes 83:---------- --ELELALAL SLVETKDYE- LSAP------ ---------V HEPQLK-QPG 113**

**ENSKMAP00000018790_Kryptolebias_marmoratus 79:---------- --ELELALTL SLLDMEDTG- MSTT------ ---------D QEPQLL-QSE 109**

**ENSCVAP00000003000_Cyprinodon_variegatus 48:---------- --ELEIALAL SLLEMKENQ- ISLP------ ---------L KKSEFT---- 75**

**ENSPFOP00000018725_Poecilia_formosa 79:---------- --ELELALAL SLLEMKDQQ- ISTP------ ---------N QLSLHP-AGG 109**

**ENSFHEP00000001658_Fundulus_heteroclitus 79:---------- --ELELALAL SLLEMKDNP- MPTP------ ---------N QSPLQP-EVT 109**

**ENSOMYP00000024389_Oncorhynchus_mykiss 1:---------- ---LAVALAL SLLEVKGQQ- QTNTK-PLFL DPKLQGQTDT QATATY-RPQ 44**

**ENSOTSP00005065052_Oncorhynchus_tshawytscha 80:---------- --ELAVALAL SLLEVKGQQ- QTNTK-PLFP DPKLQGQTDT QATATY-RPQ 124**

**ENSOKIP00005070961_Oncorhynchus_kisutch 80:---------- --ELAVALAL SLLEVKGQQ- QTNTK-PLFP DPKLQGQTDT QATATY-RPQ 124**

**ENSHHUP00000077840_Hucho_hucho 80:---------- --ELAVALAL SLLEVKGQE- QTDTK-PLFP DPK------P QATATY-RPQ 118**

**ENSSSAP00000016302_Salmo_salar 80:---------- --ELAVALAL SLLEVKRQQ- QTDTK-PLFP DPKL------ ---------- 109**

**ENSSTUP00000081428_Salmo_trutta 80:---------- --ELAVALAL SLLEVKRQQ- QTDTK-PLFP DPKLQGQTDT QATATY-RPQ 124**

**ENSHCOP00000008781_Hippocampus_comes 48:---------- --ELELAVTL SLLETQVHP- ---------- ---------- -RTRLQ-KYD 72**

**ENSOJAP00000007613_Oryzias_javanicus 78:---------- --DLELALAL SLMEMEDQQ- LSTQ------ ---------H L------QSE 103**

**ENSORLP00000011698_Oryzias_latipes 78:---------- --DLELALAL SLMEMEDQQ- PSTQ------ ---------H M------QAE 103**

**ENSOSIP00000024525_Oryzias_sinensis 78:---------- --DLELALAL SLMEMEDQQ- PSTQ------ ---------H M------QAE 103**

**ENSBSLP00000028581_Betta_splendens 84:---------- --ELELALAL SLMEIKDQDV LSTQ------ ---------S QESRFL-QPG 115**

**ENSATEP00000003870_Anabas_testudineus 82:---------- --ELELALAL SLLEMKDDQ- LSTS------ ---------S HKTGFL-QFG 112**

**ENSMMDP00005031819_Myripristis_murdjan 81:---------- --ELEMALSL SLLDVPAQ-- YSSA------ ---------T KESRAQ-GPT 110**

**ENSONIP00000067431_Oreochromis_niloticus 79:---------- --ELELALAL SLLDMKGQP- LQSP------ ---------S QESQPL-QSG 109**

**ENSNBRP00000029628_Neolamprologus_brichardi 79:---------- --ELELALAL SLLDMKGHP- LQSP------ ---------S QESQPL-QSG 109**

**ENSHBUP00000030531_Haplochromis_burtoni 48:---------- --ELELALAL SLLDMKGHP- LQSP------ ---------S QESQPL-QSG 78**

**ENSPNYP00000010231_Pundamilia_nyererei 87:---------- --ELELALAL SLLDMKGHP- LQSP------ ---------S QESQPL-HSG 117**

**ENSMZEP00005026771_Maylandia_zebra 87:---------- --ELELVLAL SLLDMKG--- ---------- ---------- ---------- 101**

**ENSACLP00000035664_Astatotilapia_calliptera 87:---------- --ELELALAL SLLDMKG--- ---------- ---------- ---------- 101**

**ENSLBEP00000024994_Labrus_bergylta 24:---------- --ELEVALAL SLLEMKDHQ- LSTP------ ---------S KTSQLH-QPH 54**

**ENSCLMP00005004807_Cyclopterus_lumpus 77:---------- --DLGLALAL SLMEMKDHQ- LSTP------ ---------S QESRLH-QPG 107**

**ENSCGOP00000000746_Cottoperca_gobio 82:---------- --ELELALTL SLLEVKDLQ- LSTP------ ---------S QESRRQ-PPG 112**

**ENSSPAP00000013334_Stegastes_partitus 48:---------- --ELELALAL SLLEMKDHQ- LSTP------ ---------S QELQLL-QPE 78**

**ENSAPOP00000010991_Acanthochromis_polyacanthus 79:---------- --ELELVLAL SLLEMKDHQ- PPAP------ ---------S QESQLL-QPG 109**

**ENSAPEP00000019384_Amphiprion_percula 79:---------- --VLELVLAL SLLEMKDQQ- PSVP------ ---------S QECQLLQQPG 110**

**ENSAOCP00000003852_Amphiprion_ocellaris 48:---------- --ELELVLAL SLLEMKDQQ- PSVP------ ---------S QECQLLQQPG 79**

**ENSLCRP00005003692_Larimichthys_crocea 83:---------- --ELELAMAL SLLEMKDHL- LSTP------ ---------S QESRCR-QTG 113**

**ENSSMAP00000005463_Scophthalmus_maximus 82:---------- --ELELALAL STLEMKDHQ- LSTP------ ---------G TESRLQ-QPG 112**

**ENSSLDP00000025094_Seriola_lalandi_dorsalis 89:---------- --ELEVALAL SVLEMKDHQ- MSAP------ ---------S QESRLQ-QPK 119**

**ENSLCAP00010022815_Lates_calcarifer 82:---------- --QLELALAL SVLEMKDHQ- LSSA------ ---------S QESRLQ-QL- 111**

**ENSSAUP00010027448_Sparus_aurata 83:---------- --ELELALAL SLLEMKDHR- LSTP------ ---------S QDSPPQ-QPG 113**

**ENSDLAP00005056549_Dicentrarchus_labrax 81:---------- --ELELALVL SLLEMKEQQ- LSTP------ ---------S QESRPQ-QPG 111**

**(Figure S3: 4/19)**

**EKC36382.1_Crassostrea_gigas 102:SKQ------- ---------- ---------- ---------- ---------- ---------- 104**

**ENSLLEP00000020779_Leptobrachium_leishanense 122:PKISYSE--- ----AAKKST LQSQSNQ--- ---------- ---------- ---------- 141**

**XtCRY6_LC705158 (This_Study) 115:PKISFAD--- ----AAKLSH FSHKHNK--- ---------- ---------- ---------- 134**

**ENSXLAT00005054664_Xenopus_laevis 108:AKISFAD--- ----AAKSSC YSHKYSE--- ---------- ---------- ---------- 127**

**ENSLOCP00000004832_Lepisosteus_oculatus 101:ARESQRE--- ---------- ---------- -TPFQSGSTD VCVNVAGEAS QPGAGQGVQA 136**

**ENSSFOP00015030596_Scleropages_formosus 105:VKCTRTL--- ----WLEANA SAGP------ ---------- ---------- ---------- 121**

**ENSCHAP00000044642_Clupea_harengus 113:PRS------- ----YAQLAS AMPVKGQPQG VDTQKSMGTH KPAGRVGE-- ---------- 149**

**ENSAMXP00000003765_Astyanax_mexicanus 93:---------- ---------- ---------- --------FD QLAERGSENR EP-------- 106**

**ENSDARP00000105169_Danio_rerio 110:VPRSYKT--- ----YADVRT ESSGA----- ---------- ---------- ---------- 127**

**ENSSGRP00000063675_Sinocyclocheilus_grahami 102:----YKS--- ----YADLTA VTSGVDQ--- ---------- ---------- ---------- 117**

**ENSCARP00000075848_Carassius_auratus/1801 95:----YKS--- ----YADLTT VTSGVNQ--- ---------- ---------- ---------- 110**

**ENSCARP00000074480_Carassius_auratus/1814 102:----YKS--- ----YVDLTT VTSGVNQ--- ---------- ---------- ---------- 117**

**ENSGMOP00000042713_Gadus_morhua 110:PPVWQGGY-- ----PAKVGG SIPGGATKSK TTTLPGLSYA QSAATGGRGR ---------- 153**

**ENSNFUP00015037037_Nothobranchius_furzeri 109:VTDCPSC--- ----FHQPDV VSNHQGK--- -------NCL EVT--PGR-- ---------- 137**

**ENSTRUP00000037131_Takifugu_rubripes 114:GRRNESG--- ----SVWPTS EFQTQRR--- -------SPI QPAEAASG-- ---------- 144**

**ENSKMAP00000018790_Kryptolebias_marmoratus 110:VRVSPSS--- ----FAQPKS AP-------- ---------- ---------- ---------- 124**

**ENSCVAP00000003000_Cyprinodon_variegatus 75:---------- ---------- ---------- ---------- ---------- ---------- 75**

**ENSPFOP00000018725_Poecilia_formosa 110:G---NAS--- ----SVLRNS SAKSQGN--- -------SCT QQEDVPGR-- ---------- 137**

**ENSFHEP00000001658_Fundulus_heteroclitus 110:D---DPS--- ----SVLLSS KPKSQGK--- -------TCT QPQDVPCR-- ---------- 137**

**ENSOMYP00000024389_Oncorhynchus_mykiss 45:NSSIQSQSVS PNGSSSQPPP APLPTGA--- -------SYA QLAAVGGR-- ---------- 82**

**ENSOTSP00005065052_Oncorhynchus_tshawytscha 125:NSALQSQSVS PNGSSSQPPP APLPTGA--- -------SYA QLAAVGGR-- ---------- 162**

**ENSOKIP00005070961_Oncorhynchus_kisutch 125:NSSIQSQSVS PNGSSSQPPP APLPTGA--- -------SYA QLAA-GGR-- ---------- 161**

**ENSHHUP00000077840_Hucho_hucho 119:NSSIQSQSVS PNGSSSQPPP APLPKGA--- -------SYA QLAAAGGR-- ---------- 156**

**ENSSSAP00000016302_Salmo_salar 109:---------- ---------- ---------- ---------- ---------- ---------- 109**

**ENSSTUP00000081428_Salmo_trutta 125:NSSIQSQSVS PNGSSSQPPP APLPKGA--- -------SYA QLAAAGGR-- ---------- 162**

**ENSHCOP00000008781_Hippocampus_comes 73:GVSNQSG--- ---------- ---------- ---------- ---------- ---------- 79**

**ENSOJAP00000007613_Oryzias_javanicus 104:VRTNQRA--- ----SFQLNS ASSSLEK--- -------TSI KPEDQVGR-- ---------- 134**

**ENSORLP00000011698_Oryzias_latipes 104:VRTNQSG--- ----PFQLNS ASMSLEK--- -------SSI KPEDQVGR-- ---------- 134**

**ENSOSIP00000024525_Oryzias_sinensis 104:VRTNQSG--- ----PFQLNS ASRSLEK--- -------SSI KPEDQVGR-- ---------- 134**

**ENSBSLP00000028581_Betta_splendens 116:DEG------- ---------- ---------- ---------- ---------- ---------- 118**

**ENSATEP00000003870_Anabas_testudineus 113:DKQNESR--- ----SSQLTS ESQPQGS--- -------SPT RRAEVVG--- ---------- 142**

**ENSMMDP00005031819_Myripristis_murdjan 111:DSRSLSAGII LSG-SIQQSS ASQPLGS--- -------SYA QLAASGGR-- ---------- 147**

**ENSONIP00000067431_Oreochromis_niloticus 110:DAQNQRG--- ----SVQMNS ILKPEGS--- -------NCT N--------- ---------- 133**

**ENSNBRP00000029628_Neolamprologus_brichardi 110:DAQNHRG--- ----SVQLNS ILKPEGS--- -------NCT N--------- ---------- 133**

**ENSHBUP00000030531_Haplochromis_burtoni 79:DAQNQRG--- ----SVQLNS ILKPEGS--- -------NCT N--------- ---------- 102**

**ENSPNYP00000010231_Pundamilia_nyererei 118:DAQNQRG--- ----SVQLNS ILKPEGS--- -------NCT N--------- ---------- 141**

**ENSMZEP00005026771_Maylandia_zebra 102:----QRG--- ----SVQLNS ILKPEGS--- -------NCT N--------- ---------- 121**

**ENSACLP00000035664_Astatotilapia_calliptera 102:----QRG--- ----SVQLNS ILKPEGS--- -------NCT N--------- ---------- 121**

**ENSLBEP00000024994_Labrus_bergylta 55:ESLNQRK--- ----SFQLAS VPQPQES--- -------SHT QRAAVTGR-- ---------- 85**

**ENSCLMP00005004807_Cyclopterus_lumpus 108:GRPNP----- -------LIS VSQPQGS--- -------SRI QPADVVGR-- ---------- 133**

**ENSCGOP00000000746_Cottoperca_gobio 113:DGLNQRS--- ----SVHRTS VSPLQGS--- -------SHT QLADGVGR-- ---------- 143**

**ENSSPAP00000013334_Stegastes_partitus 79:DRPNQSS--- ----SVQLNS VSKSQGS--- -------NYT QPADVPDV-- ---------- 109**

**ENSAPOP00000010991_Acanthochromis_polyacanthus 110:DGQNQSS--- ----SVQLSS GSKSQGS--- -------NDT QPADVQGT-- ---------- 140**

**ENSAPEP00000019384_Amphiprion_percula 111:GRHNQSS--- ----SVQLSS GSKSQGS--- -------SYT QLADVPGT-- ---------- 141**

**ENSAOCP00000003852_Amphiprion_ocellaris 80:DRHNQSS--- ----SVQLSS GSKSQGS--- -------SYT QLADVPGT-- ---------- 110**

**ENSLCRP00005003692_Larimichthys_crocea 114:DRADR----- ---------- ---------- ---------- ---------- ---------- 118**

**ENSSMAP00000005463_Scophthalmus_maximus 113:DTPSQSS--- ----SVHLNS VSQPQGS--- -------SHT QQADVRGQ-- ---------- 143**

**ENSSLDP00000025094_Seriola_lalandi_dorsalis 120:DRPNQSS--- ----SVQLTS VSQPQGS--- -------SHT QLADVAGR-- ---------- 150**

**ENSLCAP00010022815_Lates_calcarifer 112:----QSS--- ----SAQLTS VSKPQGS--- -------SHT QLTDVAA--- ---------- 137**

**ENSSAUP00010027448_Sparus_aurata 114:DRPNQRR--- ----FVQLAS VTQPQGS--- -------SHA QLADVVGR-- ---------- 144**

**ENSDLAP00005056549_Dicentrarchus_labrax 112:DRLNQRS--- ----SLQLIS VSQPQGS--- -------SHT QVVDVVGR-- ---------- 142**

**(Figure S3: 5/19)**

**EKC36382.1_Crassostrea_gigas 105:-RNTSSNSE- ---------- ------RNQP KNVNNSYS-- ---------- ---------- 124**

**ENSLLEP00000020779_Leptobrachium_leishanense 142:-RSLLSTKDV KPL-DTLSVV EV-----TQS LTRLQT---- ---DKQNSNN IDHLY---HG 184**

**XtCRY6_LC705158 (This_Study) 135:-KNSSSKTEM TKLKDNVAAM NL-----YQE RKRYHI---- ---NGQEKTC ISNCY---NG 178**

**ENSXLAT00005054664_Xenopus_laevis 128:-KTSSSKTEI DKLKHNVAAM HL-----SQE TNRCQA---- ---IRQEKT- LSSCY---NG 170**

**ENSLOCP00000004832_Lepisosteus_oculatus 137:LKATSSSGE- -----CDPRL EQLSSKGCQP PSRERAG--- ---------- ---------- 167**

**ENSSFOP00015030596_Scleropages_formosus 122:-RDGWSSRK- ---------- ------SNSM SERANS---- ---------- ---------- 139**

**ENSCHAP00000044642_Clupea_harengus 150:-RSPNGLKE- -----PGAT- -------RPA ATANEG---- ---RTDSSLA NDS------- 180**

**ENSAMXP00000003765_Astyanax_mexicanus 107:-GDPEKLIQ- -----KGCTL KH----IRPQ RDSTDT---- ---YTSGPSL VVD------- 141**

**ENSDARP00000105169_Danio_rerio 128:---------- -----DGSVV PE---NQPTA KCTLDT---- ---EVHKTRN AIKH------ 156**

**ENSSGRP00000063675_Sinocyclocheilus_grahami 118:-----SNVN- -----DGAVV AE---NQAVA RWTLNT---- ---EVHNTTC AIRN------ 150**

**ENSCARP00000075848_Carassius_auratus/1801 111:-----SNIS- -----DGAEE AE---NQAGA KWAPDT---- ---EVHNTTC AIKN------ 143**

**ENSCARP00000074480_Carassius_auratus/1814 118:-----SNIS- -----DGAEE AE---NQAGA KWAPDT---- ---EVHNTTC AIKN------ 150**

**ENSGMOP00000042713_Gadus_morhua 154:-RPSENLPK- -----TGSGV PS-----STP PTHPSRGRAP LPTDTHSTTS QTTRHKHVPG 201**

**ENSNFUP00015037037_Nothobranchius_furzeri 138:-EANSYSVL- -----TG--T EE-----GQP HINQKT---- ---GQHKNTH INSDAKQTPE 176**

**ENSTRUP00000037131_Takifugu_rubripes 145:-TRN-NSPQ- -----TGPSP KV-----CSP RTAVEP---- ---EHNADAW T--------- 175**

**ENSKMAP00000018790_Kryptolebias_marmoratus 125:--KSTSSAP- -----PGSRI KE-----CPT QINQET---- ---DPQKSLR SNSDNAQTHG 164**

**ENSCVAP00000003000_Cyprinodon_variegatus 76:-------EQ- -----SRSKT K--------- ETFLDI---- ---ENDKSLS FKSGDNQSLG 106**

**ENSPFOP00000018725_Poecilia_formosa 138:-EKSAFVAQ- -----SMSGS K--------- ENFREN---- ---QTYKSTH VEGVN-KSLG 173**

**ENSFHEP00000001658_Fundulus_heteroclitus 138:-GKSALPVQ- -----SSSKS K--------- ENLPET---- ---KTHKAMH VESADHQSLG 174**

**ENSOMYP00000024389_Oncorhynchus_mykiss 83:-RQTQAQHQD ----STSPRR SSG--HGSSP QTESQR---- ---------- ---------- 111**

**ENSOTSP00005065052_Oncorhynchus_tshawytscha 163:-RQTQAQHQD ----STSPRR SSG--HGSSP QTERQRG--- ---PTQKQTD IKW-----SN 204**

**ENSOKIP00005070961_Oncorhynchus_kisutch 162:-RRTQTQHQD ----STSPRR SSG--HGSSP QTERQRG--- ---QTQKQTD IKW-----SN 203**

**ENSHHUP00000077840_Hucho_hucho 157:-RQTEAQHQD ----STSPRR SSG--YGSSP QTERQRG--- -PTQTQKQTD IKW-----SN 200**

**ENSSSAP00000016302_Salmo_salar 110:--------QD ----STSPRR SSG--HGSSP QTERQRG--- -PTKTQKQTD IKW-----SN 146**

**ENSSTUP00000081428_Salmo_trutta 163:-RQTQAQHQD ----TTSPRR SSG--HGSSP QTERQRG--- -PTKTQKQTD IKW-----SN 206**

**ENSHCOP00000008781_Hippocampus_comes 80:-----HSKE- -----GGG-- ---------- -TTPEP---- ---AHQQETF LNM-----SG 103**

**ENSOJAP00000007613_Oryzias_javanicus 135:--GNDRMVK- -----TGFTN DE-------- SKTQDN---- ---DPQ---N NDK------- 161**

**ENSORLP00000011698_Oryzias_latipes 135:--ENDPVAK- -----SGLKS KE-----SPN SESRDI---- ---APQRGTH ENQ------- 167**

**ENSOSIP00000024525_Oryzias_sinensis 135:--ENDPVAK- -----PGLKS KE-----SPD SESQDI---- ---APQRGTH DNQ------- 167**

**ENSBSLP00000028581_Betta_splendens 119:---------- ---------L KQ-----TPP ENA------- ---------- --------VG 129**

**ENSATEP00000003870_Anabas_testudineus 143:---------- ---------- ---------- --TQET---- ---ELQKRPR VKKY------ 157**

**ENSMMDP00005031819_Myripristis_murdjan 148:-KKHNT---- ---------- ---------- DSLRPE---- ---KLQKNTH SDK--DVQGG 173**

**ENSONIP00000067431_Oreochromis_niloticus 134:--------Q- -----TGSWK RE-----SAP QTAREM---- ---EFQISTY PN---KQTLG 164**

**ENSNBRP00000029628_Neolamprologus_brichardi 134:--------Q- -----TGSWN RE-----SPP QTAREM---- ---EFQISTS PN---KQTLG 164**

**ENSHBUP00000030531_Haplochromis_burtoni 103:--------Q- -----TGSWN TE-----SPP QTAREM---- ---EFQISTY PN---KQTLG 133**

**ENSPNYP00000010231_Pundamilia_nyererei 142:--------Q- -----TGSWN RE-----SPP QTAREM---- ---EFQISTY PN---KQTLG 172**

**ENSMZEP00005026771_Maylandia_zebra 122:--------Q- -----TGSWN RE-----SPP QTAREM---- ---EFQISTY PN---KQTLG 152**

**ENSACLP00000035664_Astatotilapia_calliptera 122:--------Q- -----TGSWN RE-----SPP QTAREM---- ---EFQISTY PN---KQTLG 152**

**ENSLBEP00000024994_Labrus_bergylta 86:-KENPNLPN- -----TGARV K--------- ----GN---- ---QSRQRMS KDKCNCECPY 118**

**ENSCLMP00005004807_Cyclopterus_lumpus 134:-RKTHNSSQ- -----TGPWV KA-----NPP QTNQEA---- ---VLQKSTH VDKYEKETPG 174**

**ENSCGOP00000000746_Cottoperca_gobio 144:-REHTSSPQ- -----TGPWV KS-----SPP QTIQKA---- ---ELQKSTH VDKYKKETAG 184**

**ENSSPAP00000013334_Stegastes_partitus 110:-RKNTDSAQ- -----TGPWR NG-----SPP QTTRET---- ---ELQKSTH LDKHKKQTPG 150**

**ENSAPOP00000010991_Acanthochromis_polyacanthus 141:-RKNTDSAQ- -----TGPWR KE-----SPP QTTKET---- ---ELLKCIQ LDKHKKQTPG 181**

**ENSAPEP00000019384_Amphiprion_percula 142:-RKNTDSAQ- -----TGPWR KE-----SPP QTTKET---- ---ELLKSIQ LDKRKKQTPG 182**

**ENSAOCP00000003852_Amphiprion_ocellaris 111:-RKNTDSAQ- -----TGPWR KE-----SPP QTTKET---- ---ELLKSIQ LDKRKKQTPG 151**

**ENSLCRP00005003692_Larimichthys_crocea 119:-RKNINLPQ- -----GEPWV KV-----SPP QTARET---- ---DLEKSTH VYKYNTETLG 159**

**ENSSMAP00000005463_Scophthalmus_maximus 144:-RKKKHLAQ- -----TGPWG MV-----CPP QITQET---- ---ELQKSTN VDKGGKGTTG 184**

**ENSSLDP00000025094_Seriola_lalandi_dorsalis 151:-KKNSHSAQ- -----SGPWG MV-----CPP QTTRET---- ---EPQKSTH VDKYNKWTPG 191**

**ENSLCAP00010022815_Lates_calcarifer 138:--ENTYSAQ- -----TGPLG MV-----CPP KTTQET---- ---ELQRSTH VDKNNKETKG 177**

**ENSSAUP00010027448_Sparus_aurata 145:-GKNTNSSR- -----GAPWV KS-----SPP QTTRET---- ---GLQESAH VYKCNKETPG 185**

**ENSDLAP00005056549_Dicentrarchus_labrax 143:-RENTNSPQ- -----TEP-- ---------- -TTRGT---- ---ELRENTQ VYKYNKEAPE 175**

**(Figure S3: 6/19)**

**NoLS**

**EKC36382.1_Crassostrea_gigas 125:---------- ---------- --------KN VKDDNDSVTS SVKSKKRKNK G--------- 147**

**ENSLLEP00000020779_Leptobrachium_leishanense 185:QPEP------ --EDCVLKSA ENSSQ---FH LESLVDNGSK VKKSRRNRKK KT-------L 226**

**XtCRY6_LC705158 (This_Study) 179:QPEP------ --EDCVLKSE DGEDV---FH VETSRPRESK AKHSRRSRKK KK-------S 220**

**ENSXLAT00005054664_Xenopus_laevis 171:QPEP------ --EDCVLKSV DCEEV---FH VEASVPRESK AKQSRRRRKK TK-------S 212**

**ENSLOCP00000004832_Lepisosteus_oculatus 168:---------- ---------- --------GT PPGNTSQPDG SRSSRRRRTR KK------AS 193**

**ENSSFOP00015030596_Scleropages_formosus 140:---------- ----CSLLPC TDNKNVP--- ---EEVTASC PGRAKSSRRK RRSGKQQ--L 177**

**ENSCHAP00000044642_Clupea_harengus 181:---------- ---------- ---DS---QE KLDSNDQSEK SRKSRRRRQR KQ--QQQ--- 209**

**ENSAMXP00000003765_Astyanax_mexicanus 142:---------- ---------- ---DV---PL KNEDRGHLTK PKKS--RRHR KK---QQ--- 167**

**ENSDARP00000105169_Danio_rerio 157:---------- ---------- ---------- -----INGDK ITKSQKRRLR KK---QH--- 175**

**ENSSGRP00000063675_Sinocyclocheilus_grahami 151:---------- ---------- --------TN KDNDDLKVTK STRSQRRRQR KK---PH--- 176**

**ENSCARP00000075848_Carassius_auratus/1801 144:---------- ---------- --------TN KENDDLKVTK STRSQKRRLR KK---QH--- 169**

**ENSCARP00000074480_Carassius_auratus/1814 151:---------- ---------- --------TN KENDDLKVTK STRSQKRRLR KK---QH--- 176**

**ENSGMOP00000042713_Gadus_morhua 202:LPAPPTFHQ- --SDRRQDAV NDDARDPLDT EGTCPDQSQK PKRSKNRRQR GKGCGQRAVV 258**

**ENSNFUP00015037037_Nothobranchius_furzeri 177:TGQSPQISGS SASFSVPGMS HEGDH---IT E--DLNTSDK PKRSRNRRQR RKGTHQQ-LV 230**

**ENSTRUP00000037131_Takifugu_rubripes 176:---------- ----PAKARS ASCHQ---VM EAGDVDQSEK PKRPKKRRQR RKCAAQQ-VV 217**

**ENSKMAP00000018790_Kryptolebias_marmoratus 165:TGQALCVRGS SASFSKPDAA SEGDQ---MV AEGDSNKSDK PKRSKNRRQR RKGTGQQ-LV 220**

**ENSCVAP00000003000_Cyprinodon_variegatus 107:SSQPPYIPGS SASFTIPTTA SKSDQ---MI -RGDGNVSEK PKRPKSRRQR RKAAAQQ-VV 161**

**ENSPFOP00000018725_Poecilia_formosa 174:TSQALYVPDP SASFSIPNTA SISGQ---VM KEGDLNLSKK PRRSKNRRQR RKAAAQQ-IV 229**

**ENSFHEP00000001658_Fundulus_heteroclitus 175:TSQALRVPVS SASFSVPNAT SKSDQ---TM QEGDVNPSEK PRRPKSRRQR RKAAAQQ-IV 230**

**ENSOMYP00000024389_Oncorhynchus_mykiss 112:---------- ---------- ---------- ----VDQSEK PKRSKNRRQR RKGYGQQ-VV 136**

**ENSOTSP00005065052_Oncorhynchus_tshawytscha 205:VTQDVCASIA T--LTSNVNQ TKDDV---TV VECKVDQSEK PKRSKNRRQR RKGYGQQ-VV 258**

**ENSOKIP00005070961_Oncorhynchus_kisutch 204:VTQDVCASIA T--LTSNLNQ TKDDV---TV VECKVDQSEK PKRSKNRRQR RKGYGQQ-VV 257**

**ENSHHUP00000077840_Hucho_hucho 201:LTQDVCASIA T-SLTSNLNQ IKDDV---TV VECEVDQSEK PKRSKNRRQR RKGYGPQ-VV 255**

**ENSSSAP00000016302_Salmo_salar 147:VTQDVCASIA T-SLTSNLNQ TKDDV---TV VECEVDQSEK PKRSKNRRQR RKGYGQQ-VV 201**

**ENSSTUP00000081428_Salmo_trutta 207:VTQDVCASIA T-SLTSNLNQ TKGDV---TV VECEVDQSEK PKRSKNRRQR RKGYGQQ-VV 261**

**ENSHCOP00000008781_Hippocampus_comes 104:TCQTVS---- ----AKQDMG SKSDM----- ---MEDQCEK TNHSKSRRQR RKAAAQH-VV 146**

**ENSOJAP00000007613_Oryzias_javanicus 162:---------- --AQTKKDAV EESTN---LM ENQTLNQSEK TNRSKSRRKR RKGAV-G-LI 204**

**ENSORLP00000011698_Oryzias_latipes 168:---------- --AQTKQDAF KEPQN---LA KDQTLNQPEE PKRSKSRRKR RKGAA-S-LV 210**

**ENSOSIP00000024525_Oryzias_sinensis 168:---------- --AQTKQDAF KEPQN---LV KDQTLNQSEE PKRSKSRRKR RKGAA-S-LV 210**

**ENSBSLP00000028581_Betta_splendens 130:TCAAEAI--- ---------- ARGVP---VM EEGGVHQSEK GNRVKSRRRR HRAARQQ-AV 172**

**ENSATEP00000003870_Anabas_testudineus 158:---------- ---------- EECDQ---MT S----NLAEK SK--KNRRQR RKAARQQ-VV 187**

**ENSMMDP00005031819_Myripristis_murdjan 174:TSQNVCVSQL HTSSPKQDTG TEEDR---LV QEESLEKTEK PKRSKNRRQR RKGGGQQ-VV 229**

**ENSONIP00000067431_Oreochromis_niloticus 165:TDQTVCVSE- ---------- ---------- QEGDVDQSQK PKRSKNRRQR RKCTGQH-LV 202**

**ENSNBRP00000029628_Neolamprologus_brichardi 165:TGQTVCVSE- ---------- ---------- QEGDVDQSQK PKRSKNRRQR RKCTGQH-LV 202**

**ENSHBUP00000030531_Haplochromis_burtoni 134:TGQTVCVSE- ---------- ---------- QEGDVDQSQK PKRSKNRRQR RKCTGQH-LV 171**

**ENSPNYP00000010231_Pundamilia_nyererei 173:TGQTVCVSE- ---------- ---------- QEGDVDQSQK PKRSKNRRQR RKCTGQH-LV 210**

**ENSMZEP00005026771_Maylandia_zebra 153:TGQTVCVSE- ---------- ---------- QEGDVDQSQK PKRSKNRRQR RKCTGQH-LV 190**

**ENSACLP00000035664_Astatotilapia_calliptera 153:TGQTVCVSE- ---------- ---------- QEGDVDQSQK PKRSKNRRQR RKCTGQH-LV 190**

**ENSLBEP00000024994_Labrus_bergylta 119:TFR------- --AFPKQDTV EGGDQ---MM NMGDLTENEK PKRSKNRRQR RKGACHH-VV 165**

**ENSCLMP00005004807_Cyclopterus_lumpus 175:TSQTVCVSGL SCSIPKHNTV EESDK---MM AEGDLNPSEK PKRSKNRRQR RKGASQQ-VV 230**

**ENSCGOP00000000746_Cottoperca_gobio 185:SSRTVCVSTP SGLCSKLDTG EGGDQ---M- -EGDLNQSEK PKRSKNRRQR RKGASQQ-VV 238**

**ENSSPAP00000013334_Stegastes_partitus 151:PGQTV----- --SFSKQDAV REGDQ---RM GTEDLIQSEK PKHSKNRRQR RKGAGQQ-LV 199**

**ENSAPOP00000010991_Acanthochromis_polyacanthus 182:TGQTV----- ----SQQDTV RADDE---MI RAEDLNQSEK PKRSKNRRQR RKGAGQQ-LL 228**

**ENSAPEP00000019384_Amphiprion_percula 183:TGQTV----- ----SRQDTV RADDE---MM GEEDLNQSEK PKRSKNRRQR RKGAGQQ-LL 229**

**ENSAOCP00000003852_Amphiprion_ocellaris 152:TGQPV----- ----SRQDTV RADDE---MM GEEDLNQSEK PKRSKNRRQR RKGAGQQ-LL 198**

**ENSLCRP00005003692_Larimichthys_crocea 160:TSQTVCVSTL SGSISKQDKF EQGYQ---KM NEGDSNQSEK PKRSKNRRQR RKGPCQQ-IV 215**

**ENSSMAP00000005463_Scophthalmus_maximus 185:TSQSAYVSRP PASFSTQKTV LKSDQ---TM EEGDLNRTEK PKRSRNRRKR REGGGQQ-VV 240**

**ENSSLDP00000025094_Seriola_lalandi_dorsalis 192:TSQTESVSKP SASFSKQELV IEGGQ---MT KEGDLNQSGK PKRSKNRRQR RKAGGQQ-VV 247**

**ENSLCAP00010022815_Lates_calcarifer 178:TCQTVCVSRP SASFSRQEMV VEGDQ---TV QEGD--QSEK PKRSKNRRQR RKGGSQQ-VV 231**

**ENSSAUP00010027448_Sparus_aurata 186:TSKTECVSRL SGSISKQATG EEDNQ---MM DVGDLTESQK PKRSKNRRQR RKGAGQQ-VV 241**

**ENSDLAP00005056549_Dicentrarchus_labrax 176:TSQTVCVSRL SVSFSKQEMV EEGDQ---VM GEGDLNKSDK PKRSKNRRQR RKGTGQQ-VV 231**

**(Figure S3: 7/19)**

**EKC36382.1_Crassostrea_gigas 148:------STNR PVVYWFRRDL RLYDNPALFE AASMNVPVIL VFLWSESEED PEGV-VAAGG 200**

**ENSLLEP00000020779_Leptobrachium_leishanense 227:PTPNSFFSMK PVLVWFRRDL RLYDNPALIA ALERGAPIIP VFLWCLTEES GQNFSLASGG 286**

**XtCRY6_LC705158 (This_Study) 221:APSRGLVAMK PVLVWFRRDL RLHDNPALIS ALEHGVPVIP VFLWCINEET GQNFTLATGG 280**

**ENSXLAT00005054664_Xenopus_laevis 213:APSGGLVAMK PVLLWFRRDL RLHDNPALVS ALEHGVPVIP VFLWCINEET GQNFTLATGG 272**

**ENSLOCP00000004832_Lepisosteus_oculatus 194:SVHRSPSMPR PVLVWIRRDL RLSDNPALVG SLELGAPVIP VFLWCPREEE GPGVTVAVGS 253**

**ENSSFOP00015030596_Scleropages_formosus 178:AISRFPSFLK PILLWFRRDL RVSDNPVLIA SLEMGVPIIP VFLWCPKEEE GPGITVATGG 237**

**ENSCHAP00000044642_Clupea_harengus 210:-LLKSPSSPR PILLWFRRDL RLLDNPALIG CLELGAPVIP VFLWNAQEEE GPGVTVATGG 268**

**ENSAMXP00000003765_Astyanax_mexicanus 168:-MLKNPSAPR PVLLWLRRDL RMWDNPALIG CLELGAPIIP VFLWNATEEE GSGLTKAAGG 226**

**ENSDARP00000105169_Danio_rerio 176:-LLKNPSGPR PVVLWFRRDL RMWDNPALIG CLELGAPVIP VFLWNAMEEE GPGVTMSTGG 234**

**ENSSGRP00000063675_Sinocyclocheilus_grahami 177:-LLKNPSGPR PVLLWFRRDL RMWDNPALIG CLELGAPVIP VFLWNAVEEE GPGVTVATGG 235**

**ENSCARP00000075848_Carassius_auratus/1801 170:-LLKNPSGPR PVLLWFRRDL RMWDNPALIG CLELGAPVIP VFLWNVVEEE GPGVTVATGG 228**

**ENSCARP00000074480_Carassius_auratus/1814 177:-LLKNPSGPR PVLLWFRRDL RMWDNPALIG CLELGAPVIP VFLWNVVEEE GPGVTVATGG 235**

**ENSGMOP00000042713_Gadus_morhua 259:GLPHSPSATP PVLLWLRRDL RLHDNPALIG SLQAGAPVVP VFIWSPEEEE GPGVTMATGG 318**

**ENSNFUP00015037037_Nothobranchius_furzeri 231:GLPCSPSTPP PVVLWFRRDL RLCDNPALIR SLELGAPVIP VFIWSPKEEE GPGVTVAMGG 290**

**ENSTRUP00000037131_Takifugu_rubripes 218:GLPRAPSAAA PVLLWFRRDL RLSDNPALVS ALKVGAPVIP IFIWSPEEEE GPGVTVAMGG 277**

**ENSKMAP00000018790_Kryptolebias_marmoratus 221:SSPHSLLAPP PVLLWFRRDL RLCDNPALVG SLELGAPVIP VFIWSPEEEE GPGITVAMGG 280**

**ENSCVAP00000003000_Cyprinodon_variegatus 162:GLIRSPSAPP PVLLWFRRDL RLCDNPALIG SLEVGAPVIP IFIWSPEEEE GPGVTVAMGG 221**

**ENSPFOP00000018725_Poecilia_formosa 230:GLARSPSAPP PVLLWFRRDL RLCDNPALIR CLELGAPVIP VFIWSPEEEE GPGVTVAMGG 289**

**ENSFHEP00000001658_Fundulus_heteroclitus 231:GLARSPSAPP PVLLWFRRDL RLCDNPALIR SLELGAPVIP VFIWCPEEEE GPGVTVAMGG 290**

**ENSOMYP00000024389_Oncorhynchus_mykiss 137:GVPRCPSAPP PVLLWFRRDL RLHDNPAVIG SLEAGGPVIP VFIWCPEEEE GPGVTVAMGG 196**

**ENSOTSP00005065052_Oncorhynchus_tshawytscha 259:GVPRCPSAPP PVLLWFRRDL RLHDNPAVIG SLEAGGPVIP VFIWCPEEEE GPGVTVAMGG 318**

**ENSOKIP00005070961_Oncorhynchus_kisutch 258:GVPRCPSAPP PVLLWFRRDL RLHDNPAVIG SLEAGGPVIP VFIWCPEEEE GPGVTVAMGG 317**

**ENSHHUP00000077840_Hucho_hucho 256:GVPRCPSAPP PVLLWFRRDL RLHDNPAVIG SLEAGGPVIP VFIWCPEEEE GPGATVAMGG 315**

**ENSSSAP00000016302_Salmo_salar 202:GVPRCPSAPP PVLLWFRRDL RLHDNPAVIG SLEAGGPVIP VFIWCPEEEE GPGVTVAMGG 261**

**ENSSTUP00000081428_Salmo_trutta 262:GVPRCPSAPP PVLMWFRRDL RLHDNPAVIS SLEAGGPVIP VFIWCPEEEE GPGVTVAMGG 321**

**ENSHCOP00000008781_Hippocampus_comes 147:GLPDSPSGAA PVLLWVRRDL RLCDNPALVG SLELGAPVIP VFIWSPKEEE GPGMTVAMGG 206**

**ENSOJAP00000007613_Oryzias_javanicus 205:GLPGSPSASP PVLLWFRRDL RLCDNPALHA ALEMGAPVIP IFIWSSEEEE GPGVTVAAGG 264**

**ENSORLP00000011698_Oryzias_latipes 211:GLPGSPSASP PVLLWFRRDL RLCDNPALNA ALEMDAPVIP IFIWSPEEEE GPGVTVAAGG 270**

**ENSOSIP00000024525_Oryzias_sinensis 211:GLPGSPSASP PVLLWFRRDL RLCDNPALNA ALEMDAPVIP IFIWSPEEEE GPGVTVAAGG 270**

**ENSBSLP00000028581_Betta_splendens 173:GLPSSLSAPP PVLLWFRRDL RLCDNPALSA SLECGAPVIP VFIWSPEEEE GPGTTLATGG 232**

**ENSATEP00000003870_Anabas_testudineus 188:GLQCSLSAPP PVLVWFRRDL RLCDNPALSA SLELGAPVIP VFIWSPDEEE GPGITLAIRG 247**

**ENSMMDP00005031819_Myripristis_murdjan 230:GLPRSPSGPP PVLLWFRRDL RLCDNPALIG SLELGAPVIP VFIWSPEEEE GPGVTVAVGG 289**

**ENSONIP00000067431_Oreochromis_niloticus 203:GLPRSPSATP PVLLWFRRDL RLCDNPALVA SLEVGAPVIP VFIWSPEEEE GTGVTVAMGG 262**

**ENSNBRP00000029628_Neolamprologus_brichardi 203:GLPRSPSAPP PVLLWFRRDL RLCDNPALVA SLEVGAPVIP VFIWSPEEEE GTGITVAMGG 262**

**ENSHBUP00000030531_Haplochromis_burtoni 172:GLPRSPSAPP PVLLWFRKDL RLCDNPALVA SLEVGAPVIP VFIWSPKEEE GTGITVAMGG 231**

**ENSPNYP00000010231_Pundamilia_nyererei 211:GLPRSPSAPP PVLLWFRRDL RLCDNPALVA SLEVGAPVIP VFIWSPKEEE GTGITVAMGG 270**

**ENSMZEP00005026771_Maylandia_zebra 191:GLPRSPSAPP PVLLWFRRDL RLCDNPALVA SLEVGAPVIP VFIWSPKEEE GTGITVAMGG 250**

**ENSACLP00000035664_Astatotilapia_calliptera 191:GLPRSPSAPP PVLLWFRRDL RLCDNPALVA SLEVGAPVIP VFIWSPKEEE GTGITVAMGG 250**

**ENSLBEP00000024994_Labrus_bergylta 166:GLPPSPSARP PVLLWFRRDL RLCDNPALNG SLEVGAPVIP IFIWSPEEEE GPGITVAMGG 225**

**ENSCLMP00005004807_Cyclopterus_lumpus 231:GLPCSPSALP PVLLWFRRDL RLGDNPALIG SLEVGAPVIP VFIWSPEEEE GPGITVAMGG 290**

**ENSCGOP00000000746_Cottoperca_gobio 239:GLPCSPSAPP PVLLWFRRDL RLGDNPALTG SLEVGAPVIP VFIWSPEEEE GPGITVAMGG 298**

**ENSSPAP00000013334_Stegastes_partitus 200:GLPCSPSAPP PVLLWFRRDL RLCDNPALMG SLEVGAPVIP VFIWSPEEEE GPGVTVAMGG 259**

**ENSAPOP00000010991_Acanthochromis_polyacanthus 229:GLPGSPSAPP PVLLWFRRDL RLCDNPALMG SLEVGAPVIP VFIWSPEEEE GPGITVAMGG 288**

**ENSAPEP00000019384_Amphiprion_percula 230:GLPGSPSAAP PVLLWFRRDL RLCDNPALMG SLEVGAPVIP VFIWSPEEEE GPGITVAMGG 289**

**ENSAOCP00000003852_Amphiprion_ocellaris 199:GLPGSPSAPP PVLLWFRRDL RLCDNPALMG SLEVGAPVIP VFIWSPEEEE GPGITVAMGG 258**

**ENSLCRP00005003692_Larimichthys_crocea 216:GLPCSPSAPP PVLLWFRRDL RLRDNPALIC SLEFGAPVIP VFIWSPEEEE GPGNTVAMGG 275**

**ENSSMAP00000005463_Scophthalmus_maximus 241:GLPSSPSAPS PVLLWFRRDL RLCDNPALIG SLEVGAPVIP VFIWSPEEEE GPGITVAMGG 300**

**ENSSLDP00000025094_Seriola_lalandi_dorsalis 248:GLPCSPSAPQ PVLLWFRRDL RLCDNPALIG SLEVGAPVIP VFIWSPEEEE GPGITVAVGG 307**

**ENSLCAP00010022815_Lates_calcarifer 232:GLPHSPSAQP PVLLWFRRDL RLCDNPALIG SLEVGAPVIP VFIWSPEEEE GPGITVAVGG 291**

**ENSSAUP00010027448_Sparus_aurata 242:GLPCSPSAQP PVLLWFRRDL RLCDNPALIG SLEAGAPVIP VFIWSPEEEE GPGITVAMGG 301**

**ENSDLAP00005056549_Dicentrarchus_labrax 232:GLPCSPSAPP PVLLWFRRDL RLCDNPALNG SLEVGAPVIP VFIWSPEEEE GPGITVAMGG 291**

**(Figure S3: 8/19)**

**EKC36382.1_Crassostrea_gigas 201:ATKLWLHHAL NHLDKSISDR YNNRIIYRKT ------QSCQ REILSLIEET GAKALLINDV 254**

**ENSLLEP00000020779_Leptobrachium_leishanense 287:ATRYWLHHAL LQLNNSLKDK YSSQLIFRVS ------ESCA HELMTLVKET GAETVIVNSV 340**

**XtCRY6_LC705158 (This_Study) 281:ATKYWLHHAL LKLNQSLIQR FGSHIIFRVA ------RSCE EELVSLVHET GADTIVINAV 334**

**ENSXLAT00005054664_Xenopus_laevis 273:ATKYWLHHAL LELNQSLIQK FGSHVIFRVA ------QSCE KELVSLVHET GAGTVIVNAV 326**

**ENSLOCP00000004832_Lepisosteus_oculatus 254:ASKYWLHHAL LCFIQSLHKL GGHLVTRRVE ------TTTQ QALQSLVSET GADTLLANAL 307**

**ENSSFOP00015030596_Scleropages_formosus 238:ASKYWLHHAL LCLNQSLEKL GSHLVTVEAE ------TSSL EALQGLAAET GASGVVAAAL 291**

**ENSCHAP00000044642_Clupea_harengus 269:ACKYWLHQAL VCLNEALERI GSHLVTVKAE ------PSSS EALQRLVEET GAGAVVATAL 322**

**ENSAMXP00000003765_Astyanax_mexicanus 227:ASKYWMHQAL VCLNRSLEER GSHLVTQRAE ------PSSL TALQALVAET GAGTVVATAL 280**

**ENSDARP00000105169_Danio_rerio 235:ASKYWLHQAL VSLKRSLEER GSHLVTLKAE ------PSSL TALQGLMDET GAASVVATAL 288**

**ENSSGRP00000063675_Sinocyclocheilus_grahami 236:ASKYWLHQAL VSLNRSLEQR RSHLVTLKAE ------ASSL TALQGLITET GAASVVATAL 289**

**ENSCARP00000075848_Carassius_auratus/1801 229:ASKYWLHQAL VSLNRSLEKC GSNLVTLKAE ------VSSL TALQGLIAET GAASVVATAL 282**

**ENSCARP00000074480_Carassius_auratus/1814 236:ASKYWLHQAL VSLNRSLEKC GSNLVTLKAE ------VSSL TALQGLIAET GAASVVATAL 289**

**ENSGMOP00000042713_Gadus_morhua 319:ACRYWLHQAL ACLQGALQLL GSHLVLLKAD P-----GSSL GALLGLVGET GARTVLATAL 373**

**ENSNFUP00015037037_Nothobranchius_furzeri 291:ASKYWLHQAL SCLCASLEKI GSHLIFLRAS GEGNEVGSSL HVLKQLVKTT GAQTVMANAL 350**

**ENSTRUP00000037131_Takifugu_rubripes 278:ACKYWLHQAL SCLCSSLENI GSHLVFLKSE TRGSEGGASL RALQGLAKAT GARTVVANAL 337**

**ENSKMAP00000018790_Kryptolebias_marmoratus 281:ACKYWLHQAL SCLCTSLERI GSHLVFLEAS GEGIEVRSSL HTLKQLVKET GARTIMANAL 340**

**ENSCVAP00000003000_Cyprinodon_variegatus 222:ACKYWLHQAL SCFRASLEQI GSHLVFFKAS NDVTEVGSSL NTLKTLVKET GAQSVMANAL 281**

**ENSPFOP00000018725_Poecilia_formosa 290:ACKYWLHQAL SCFCASLERI GSHLIFLNAS GDGNKDGSSL HTLKTLVKET GAQTVMANAL 349**

**ENSFHEP00000001658_Fundulus_heteroclitus 291:ACKYWLHQAL SCFCASLEQI GSRLVFLKAG GEGNERRSSL HTLKTLAKET GAQTVLANAL 350**

**ENSOMYP00000024389_Oncorhynchus_mykiss 197:ACKFWLHQAL SCLSSALEHI GSHLVFLRPD EEREGIGSSL LALRSLVRET GAQTVLASAL 256**

**ENSOTSP00005065052_Oncorhynchus_tshawytscha 319:ACKFWLHQAL SCLSSALEHI GSHLVFLQPD EDREGIGSSL LALRSLVRET GAQTVLASAL 378**

**ENSOKIP00005070961_Oncorhynchus_kisutch 318:ACKFWLHQAL SCLSSALEHI GSHLVFLRPD EEREGIGSSL LALRSLVRET GAQTVLASAL 377**

**ENSHHUP00000077840_Hucho_hucho 316:ACKFWLHQAL SCLSSALERI GSHLVVLWPD EEREGIGSSL RALRSLVRET GAQTVLASAL 375**

**ENSSSAP00000016302_Salmo_salar 262:ACKFWLHQAL SCLSSALEHI GSHLVFLRPD EEREGIGSSL RALRSLVRET GAQTVLASAL 321**

**ENSSTUP00000081428_Salmo_trutta 322:ACKFWLHQAL SCLSSALEHI GSHLVFLRPD EEREGIGSSL RALRSLVRET GAQTVLASAL 381**

**ENSHCOP00000008781_Hippocampus_comes 207:ACKYWLHQAL SSFRSSLERI GSHLVFLEVN G-----SSTL RTLKELVRET GARTVLANAL 261**

**ENSOJAP00000007613_Oryzias_javanicus 265:ASKYWLHQAL ACFCISLNRI GSHLTFLEAE K-----QSSL RTLKQLVKET GARTLLANAL 319**

**ENSORLP00000011698_Oryzias_latipes 271:ASKYWLHQAL ACICTSLNHI GSRLTFLKAD GEE---KCSL PTLKQLVKET GAKTLLANAL 327**

**ENSOSIP00000024525_Oryzias_sinensis 271:ASKYWLHQAL ACICTSLNHI GSRLTFLKAD GEE---KCSL RTLKQLVKQT GAKTLLANAL 327**

**ENSBSLP00000028581_Betta_splendens 233:ASKYWLHQAL SCLCASLEHI GSHLVYIKAH GGGAGVGSSL HSLQELIKDT GARTVLANAL 292**

**ENSATEP00000003870_Anabas_testudineus 248:AGKYWLHQAL SHLCASLERI GSHLVFLKAS GEENGVGSSL HSLKKLIKDT GARTVLANAL 307**

**ENSMMDP00005031819_Myripristis_murdjan 290:ACKYWLHQAL SCFRSSLERI GSHLVFLETD REGKDVGSSL QALKELVDET GARTVLANAL 349**

**ENSONIP00000067431_Oreochromis_niloticus 263:ACKYWLHQAL LCFCSSLEHI GSHLVFLKAN GEMTSAESSL KALKELIKDT GARTVLANAL 322**

**ENSNBRP00000029628_Neolamprologus_brichardi 263:ACKYWLHQAL LCFSSSLEHI GSRLVVLKAN GEMTSAESSL KALKELIKET GARTVLANAL 322**

**ENSHBUP00000030531_Haplochromis_burtoni 232:ACKYWLHQAL LCFCSSLKHI GSRLVVLKAN GEMTSTESSL KALKELIKET GARTVLANAL 291**

**ENSPNYP00000010231_Pundamilia_nyererei 271:ACKYWLHQAL LCFCSSLEHI GSRLVVLKA- ----STESSL KALKELIKET GARTVLANAL 325**

**ENSMZEP00005026771_Maylandia_zebra 251:ACKYWLHQAL LCFCSSLEHI GSRLVVLKAN GEMTSTESSL KALKELIKET GARTVLANAL 310**

**ENSACLP00000035664_Astatotilapia_calliptera 251:ACKYWLHQAL LCFCSSLEHI GSRLVVLKAN GEMTSTESSL KALKELIKET GARTVLANAL 310**

**ENSLBEP00000024994_Labrus_bergylta 226:ACKYWLHQAL SCFCSSLECI GSRLVFLKAK AAKNEVGPTL HCLKELVKET GATTVFANAL 285**

**ENSCLMP00005004807_Cyclopterus_lumpus 291:ACKYWLHQAL SCFSSSLECI GSHLVFFEAN ------GSSL HTLKELVKET GARTVLANAL 344**

**ENSCGOP00000000746_Cottoperca_gobio 299:ACKYWLHQAL SCFSSSLERI GSNLVLLKAN ------GSSL RSLKELVKET GTRTVLANAL 352**

**ENSSPAP00000013334_Stegastes_partitus 260:ACKYWLHQAL SCFSSSLERI GSHLVFLKAN GEGNEVGSSL LTLKELVKET GARTVLANAL 319**

**ENSAPOP00000010991_Acanthochromis_polyacanthus 289:ACKYWLHQAL SCFSSSLERI GSHLVFLKAN KEGNEVGSSL QMLKELVKET GARTVLANAL 348**

**ENSAPEP00000019384_Amphiprion_percula 290:ACKYWLHQAL SCFRSSLERI GSHLIFLKAN REGNEVGSSL QTLKELVKET GARTVLANAL 349**

**ENSAOCP00000003852_Amphiprion_ocellaris 259:ACKYWLHQAL SCFCSSLERI GSHLVFLKAN REGNEVGSSL QTLKELVKET GARTVLANAL 318**

**ENSLCRP00005003692_Larimichthys_crocea 276:ACKYWLHQAL SCFCSSLERI GSHLVFLKAN HEGNKVGSSL STLKELIKET GARTVLANAL 335**

**ENSSMAP00000005463_Scophthalmus_maximus 301:ACKYWLHQAL SCFRSSLECI GSHLIFLKAN GEGHGVGSSL HTLRELIKET GARTVLANAL 360**

**ENSSLDP00000025094_Seriola_lalandi_dorsalis 308:ACKYWLHQAL SHFCSSLESI GSHLIFLKAD GGGDGVGSSL CTLKELIKKT GARTVLANAL 367**

**ENSLCAP00010022815_Lates_calcarifer 292:ACKYWLHQAL SCFCSSLERI GSHLVFLTAN GEGNGVGSSL RSLKELIKET GARTVLANAL 351**

**ENSSAUP00010027448_Sparus_aurata 302:ACKYWLHQAL SCFSSSLESI GSHLVFLKAN RERSGVGSSV HTLKELVKET GARTVVANAL 361**

**ENSDLAP00005056549_Dicentrarchus_labrax 292:ACKYWLHQAL SCFCSSLERI GSHLVFLKAS GEGKEVGSSL RTLKQLVKET GARTVLANAL 351**

**(Figure S3: 9/19)**

**EKC36382.1_Crassostrea_gigas 255:YEPFLKQRDD KICAELQRKG IECKKFHSYL LHEPGSVSAE SVGMRGVGSV THFMECCRQS 314**

**ENSLLEP00000020779_Leptobrachium_leishanense 341:YEPWLKERDD LVSEALQKRG VTLTKCHSYC LHEPYSVRSE GVGLQGIGSV SHFMECCKRN 400**

**XtCRY6_LC705158 (This_Study) 335:YEPWLKERDD LISETLRRHG VELKKHHSYC LYEPDSVSTE GVGLRGIGSV SHFMSCCKRN 394**

**ENSXLAT00005054664_Xenopus_laevis 327:YEPWLKERDD FISENLQKNG VEFKKHHSYC LYEPGSISTE GVGLRGIGSV SHFMSCCKRN 386**

**ENSLOCP00000004832_Lepisosteus_oculatus 308:YEPWLKERDE LAFSALESQG VKCHLYHSYC LREPGSVCTE GVGLRGIGSV SHFMSCCQHN 367**

**ENSSFOP00015030596_Scleropages_formosus 292:YEPWLKERDE AVFSALESKG VKCQLRHSYC LREPSSISTE GVGLRGIGSV SHFLSCCQQN 351**

**ENSCHAP00000044642_Clupea_harengus 323:YEPWLKERDE GVFNTLKQHR LSCHLYHSYC LQDPYTVTTQ GVGLRGIGSV SHFTSCCQQN 382**

**ENSAMXP00000003765_Astyanax_mexicanus 281:YEPWLQERDE AVWATLEKQG VKCHIYHSYC LRDPNTVSTR GVGLRGIGSV SHFISCCNQN 340**

**ENSDARP00000105169_Danio_rerio 289:YEPWLKERDD ALWETLEKRG VTCHIYHSYC LRDPYTVSTR GVGLRGIGSV SHFMSCCQQN 348**

**ENSSGRP00000063675_Sinocyclocheilus_grahami 290:YEPWLKERDD SVWEKLEKQG VKCHIYHSYC LRDPYTVSTR GVGLRGIGSV SHFMSCCQQN 349**

**ENSCARP00000075848_Carassius_auratus/1801 283:YEPWLKERDD SVWEKLEKQG VKCHVYHSYC LRDPYTVSTR GVGLRGIGSV SHFMSCCQQN 342**

**ENSCARP00000074480_Carassius_auratus/1814 290:YEPWLKERDD SVWEKLEKQG VKCHVYHSYC LRDPYTVSTR GVGLRGIGSV SHFMSCCQQN 349**

**ENSGMOP00000042713_Gadus_morhua 374:YEPWLRERDQ RVEAGLRRAG VAWRMVHSYC LRDPYSVSTQ GVGLRGLGSV SHFISCCEQN 433**

**ENSNFUP00015037037_Nothobranchius_furzeri 351:FEPWLKERDD AAVAALQKGG VVCRMLQSYC LRDPYSVSTE GVGLRGIGSV SHFMSCCRQN 410**

**ENSTRUP00000037131_Takifugu_rubripes 338:YEPWLKGRDD AVAAALQKSG VECEMFHSYC LRDPYSVSTE GVGLRGIGSV SHFMSCCRQN 397**

**ENSKMAP00000018790_Kryptolebias_marmoratus 341:YEPWLRERDV GVVSALQKEG VECKMFHSYC LRDPYSVSTE GVGLRGIGSV SHFMSCCRQN 400**

**ENSCVAP00000003000_Cyprinodon_variegatus 282:YEPWLKERDD AVSSALQKEG VEFKMFHSYC LRDPFSVSTE GVGLRGIGSV SHFMSCCRQN 341**

**ENSPFOP00000018725_Poecilia_formosa 350:YEPWLKERDD VVFSALHKEG VECKMFHSYC LRDPFSVSTE GVGLRGIGSV SHFMNCCRQN 409**

**ENSFHEP00000001658_Fundulus_heteroclitus 351:YEPWLKERDD VVLSALQKEG VDCEMFHSYC LRDPFSVSTE GVGLRGIGSV SHFMSCCRQN 410**

**ENSOMYP00000024389_Oncorhynchus_mykiss 257:YEPWLRERDQ VVVSALQKDR VEVNMVHSYC LRDPYTVTTE GVGLRGIGSV SHFMSCCQMN 316**

**ENSOTSP00005065052_Oncorhynchus_tshawytscha 379:YEPWLRERDQ VVVSALQKDR VEVNMVHSYC LRDPYTVTTE GVGLRGIGSV SHFMSCCQMN 438**

**ENSOKIP00005070961_Oncorhynchus_kisutch 378:YEPWLRERDQ VVVSALQKDR VEVNMVHSYC LRDPYTVTTE GVGLRGIGSV SHFMSCCQMN 437**

**ENSHHUP00000077840_Hucho_hucho 376:YEPWLRERDQ VVVSALQKDR VEVNMVHSYC LRDPYTVTTE GVGLRGIGSV SHFMSCCQMN 435**

**ENSSSAP00000016302_Salmo_salar 322:YEPWLRERDQ VVVSALQKDR VEVNMVHSYC LRDPYTVTTE GVGLRGIGSV SHFMSCCQMN 381**

**ENSSTUP00000081428_Salmo_trutta 382:YEPWLRERDQ VVVSALQKDR VEVNMVHSYC LRDPYTVTTE GVGLRGIGSV SHFMSCCQMN 441**

**ENSHCOP00000008781_Hippocampus_comes 262:YEPWLKERDD EVVSRLQKDG VRCKMFHSYC TRDPYSVSTE GVGLRGIGSV SHFMSCCRQN 321**

**ENSOJAP00000007613_Oryzias_javanicus 320:YEPWLKERDD AVVSALQKEG VECKISHSYC LRDPYSVSTV GVGLRGIGSV SHFMSCCKQN 379**

**ENSORLP00000011698_Oryzias_latipes 328:YEPWLKERDD MVESTLQKEG VQCRIFPSYC LRDPYSVSTV GVGLRGIGSV SHFMSCCKQN 387**

**ENSOSIP00000024525_Oryzias_sinensis 328:YEPWLKERDD MVESTLQKEG VQCRIFPSYC LRDPYSVSTV GVGLRGIGSV SHFMSCCKQN 387**

**ENSBSLP00000028581_Betta_splendens 293:YEPWLKARDD AVASALQKDG TELRMFHSYC LRDPYSVSTE GVGLRGIGSV SHFMSCCKQN 352**

**ENSATEP00000003870_Anabas_testudineus 308:YEPWLKERDD AVVAALQKDG TEVRMFQSYC LRDPYSVSTE GVGLRGIGSV SHFMSCCRQN 367**

**ENSMMDP00005031819_Myripristis_murdjan 350:YEPWLKERDD AVVSALQRDG VECRMVHSYC LRDPYSVSTE GVGLRGIGSV SHFMSCCRQN 409**

**ENSONIP00000067431_Oreochromis_niloticus 323:YEPWLKERDD LVVSTLQKQG VEFRMFHSFC LRDPYSVTTE GVGLRGIGSV SHFMSCCRQN 382**

**ENSNBRP00000029628_Neolamprologus_brichardi 323:YEPWLKERDD LVVSALQKQG VECRMFHSYC LRDPYSVTTE GVGLRGIGSV SHFMSCCRQN 382**

**ENSHBUP00000030531_Haplochromis_burtoni 292:YEPWLKERDD LVVSALQKQG VECRMFHSYC LRDPYSVTTD GVGLRGIGSV SHFMSCCRQN 351**

**ENSPNYP00000010231_Pundamilia_nyererei 326:YEPWLKERDD LVVSALQKQG VECRMFHSYC LRDPYSVTTE GVGLRGIGSV SHFMSCCRQN 385**

**ENSMZEP00005026771_Maylandia_zebra 311:YEPWLKERDD LVVSALQKQG VECRMFHSYC LRDPYSVTTE GVGLRGIGSV SHFMSCCRQN 370**

**ENSACLP00000035664_Astatotilapia_calliptera 311:YEPWLKERDD LVVSALQKQG VECRMFHSYC LRDPYSVTTE GVGLRGIGSV SHFMSCCRQN 370**

**ENSLBEP00000024994_Labrus_bergylta 286:YEPWLKDRDD EVVSAFKKDG VEVKMFHSYC LKDPYSVSTE GVGLRGIGSV SHFMSCCKQN 345**

**ENSCLMP00005004807_Cyclopterus_lumpus 345:YEPWLKERDD VVVSALQKDA VECRMFHSYC VRDPYSVSTE GVGLRGIGSV SHFMDCCRQN 404**

**ENSCGOP00000000746_Cottoperca_gobio 353:YEPWLKERDD AVVSALQKDG VECRMFHSYC LRDPYSVSPE GVGLRGIGSV SHFMSCCKQN 412**

**ENSSPAP00000013334_Stegastes_partitus 320:YEPWLKDRDD VVVSALKKDG VECRMFHSYC LRDPYSVSTE GVGLRGIGSV SHFMSCCKQN 379**

**ENSAPOP00000010991_Acanthochromis_polyacanthus 349:YEPWLKERDD AVVSALQKEG VECRMFHSYC LRDPYSVSTE GVGLRGIGSV SHFMSCCKQN 408**

**ENSAPEP00000019384_Amphiprion_percula 350:YEPWLKERDD AVVSALQKEG VECRMFHSYC LRDPYSVSTE GVGLRGIGSV SHFMSCCKQN 409**

**ENSAOCP00000003852_Amphiprion_ocellaris 319:YEPWLKERDD AVVSALQKEG VECRVFHSYC LRDPYSVSTE GVGLRGIGSV SHFMSCCKQN 378**

**ENSLCRP00005003692_Larimichthys_crocea 336:YEPWLKERDD AVVSSLQKDG VEFRMFHSYC LRDPYSISTE GVGLRGIGSV SHFMSCCRQN 395**

**ENSSMAP00000005463_Scophthalmus_maximus 361:YEPWLKERDD VVVSALQEVG VQCKVFHSYC LRDPYSVSTE GVGLRGIGSV SHFMSCCRQN 420**

**ENSSLDP00000025094_Seriola_lalandi_dorsalis 368:CEPWLKERDD VVVSDLQKDG VECRMFHSYC LRDPYSVSTE GVGLRGIGSV SHFMSCCRQN 427**

**ENSLCAP00010022815_Lates_calcarifer 352:YEPWLKERDD TVVSALQKDG VECRMLHSYC LRDPYSISTE GVGLRGIGSV SHFMSCCKQN 411**

**ENSSAUP00010027448_Sparus_aurata 362:YEPWLKERDD AVVSALQKDG VECKMFDSYC LKDPYSVSTE GVGLRGIGSV SHYMSCCSQN 421**

**ENSDLAP00005056549_Dicentrarchus_labrax 352:YEPWLKERDD VVVSALQKDG VECRMVHSYC LRDPYSVTTD GVGLRGIGSV SHFMSCCRQN 411**

**(Figure S3: 10/19)**

**EKC36382.1_Crassostrea_gigas 315:D-AQPIGHPL DYPPTLPKPD QFPSSSSLHD LELAKMPRRK DGSIIDWAAP IVRQWDFGEE 373**

**ENSLLEP00000020779_Leptobrachium_leishanense 401:P-SGPIGIPL EAPTSLPLPS CWPSALEIGQ LNLARMPRRK DGTVVDWAAT IRGTWDFSED 459**

**XtCRY6_LC705158 (This_Study) 395:N-SAPIGMPL DAPRCLPAPC NWPESDHLDT LELGKMPHRK DGTLIDWAVT IRESWDFSED 453**

**ENSXLAT00005054664_Xenopus_laevis 387:Q-SAPIGIPL DAPMILPTPC KWPDSDDLDK MKLAKMPSRK DGTLIDWAMT IRESWDFSED 445**

**ENSLOCP00000004832_Lepisosteus_oculatus 368:P-APGLGSVL QAPATLPVPS QWPQSGPLDQ LDLAKMPRRK DGTTVDWAAT IRSSWDFSEE 426**

**ENSSFOP00015030596_Scleropages_formosus 352:S-KSALGTPV ETPPGLPVPS TWPQGCPLAQ LGLAKMPRRK DGTTVDWAAN IRSSWDFSEE 410**

**ENSCHAP00000044642_Clupea_harengus 383:P-TGPSGAPQ DAPRTLPVPS AWPAGCPLEQ LGLARMPRRK DGTMVDWAVN IRRDWDFTEE 441**

**ENSAMXP00000003765_Astyanax_mexicanus 341:P-GPGIGAPL DAPGALPSPS SWPQGCPLTQ LELALMPVRK DGTVIDWAAN IRQAWDFSEE 399**

**ENSDARP00000105169_Danio_rerio 349:P-AGGLGSPL DAPTTLPSPS AWPQGCPLAD LGLARMPRRK DGTVIDWAVD IRKTWDFSEE 407**

**ENSSGRP00000063675_Sinocyclocheilus_grahami 350:P-GSGLTSPL DAPAALPAPS AWPQGCPLVD LGLARMPRRK DGTEIDWAVN IRKSWDFSEE 408**

**ENSCARP00000075848_Carassius_auratus/1801 343:P-GSGQTSPL DAPAALPAPS AWPQGCPLVD LGLARMPRRK DGTEIDWAVN IRKSWDISEK 401**

**ENSCARP00000074480_Carassius_auratus/1814 350:P-GSGQTSPL DAPAALPAPS AWPQGCPLVD LGLARMPRRK DGTEIDWAVN IRKSWDISEE 408**

**ENSGMOP00000042713_Gadus_morhua 434:P-GPALGPSL DPPVSMPTPS QWPPGDPLDA LGLARMPRRK DGTVIDWAAN IRASWDFSEQ 492**

**ENSNFUP00015037037_Nothobranchius_furzeri 411:P-GSPIGVPL DPPVSLPTPA HWPQGVPLDA LGLARMPRRK DGTTVDWAAN IRKSWDFSEN 469**

**ENSTRUP00000037131_Takifugu_rubripes 398:P-GAALGAPL DPPTTLPVPA HWPPGVSLET LGLARMPKRK DGTTIDWAAN IRKSWDFSEG 456**

**ENSKMAP00000018790_Kryptolebias_marmoratus 401:P-GPPVGVPL EPAASLPTPA NWPRGIPLDS LGLARMPRRK DGTVIDWAAN IRTSWDFSEA 459**

**ENSCVAP00000003000_Cyprinodon_variegatus 342:P-GAAVGVPL DPPVSLPSPA HWPQGVPLDA LGLARMPRRK DGTIVDWAAN IRKSWDFSED 400**

**ENSPFOP00000018725_Poecilia_formosa 410:P-GASIGVPL DPPESLPSPT RWPQGVPLDM LGLARMPRRK DGTIIDWAAN IRKSWDFSED 468**

**ENSFHEP00000001658_Fundulus_heteroclitus 411:P-GPAIGVPL DPPVSLPSPA FWPQGVAMDS IDLARMPRRK DGTIIDWAAN IRKSWDFSED 469**

**ENSOMYP00000024389_Oncorhynchus_mykiss 317:P-GPGLGVPL DPPISLPSPS VWPRGCPLEG LGLARMPCRK DGTTIDWAAN IRSSWDFSEE 375**

**ENSOTSP00005065052_Oncorhynchus_tshawytscha 439:P-GPGLGVPL DPPISLPSPS VWPRGCSLEG LGLARMPCRK DGTTIDWAAN IRSSWDFSEE 497**

**ENSOKIP00005070961_Oncorhynchus_kisutch 438:P-GPGLGVPL DPPISLPSPS VWPRGCPLEG LGLARMPCRK DGTTIDWAAN IRSSWDFSEE 496**

**ENSHHUP00000077840_Hucho_hucho 436:P-GPGLGVPL DPPRSLPSPS VWPRGCPLEG LGLARMPCRK DGTTIDWAAN IRSSWDFSEE 494**

**ENSSSAP00000016302_Salmo_salar 382:P-GPGLGVPL DPPISLPSPS VWPRGCPLEG LGLARMPCRK DGTTIDWAAN IRSSWDFSEE 440**

**ENSSTUP00000081428_Salmo_trutta 442:P-GPGLGVPL DPPISLPSPS VWPRGCPLEG LGLARMPCRK DGTTIDWAAN IRSSWDFSEE 500**

**ENSHCOP00000008781_Hippocampus_comes 322:PAGTAFGAPL DPPMSLPVPS SWPQGIPLDT LGLARMPRRK DGTTIDWAEN ICKSWDFSEE 381**

**ENSOJAP00000007613_Oryzias_javanicus 380:P-GSAIGVPL DPPQTLPTPA CWPQGVPLEA LGLARMPRRK DGKIIDWAAN IRKSWDFSED 438**

**ENSORLP00000011698_Oryzias_latipes 388:P-GSAIGVPL DPPQSLPTPS CWPQGVPLEA LGLARMPRRK DGTTIDWAAN IRKSWDFSED 446**

**ENSOSIP00000024525_Oryzias_sinensis 388:P-GSAIGVPL DPPQSLPTPS CWPQGVPLEA LGLARMPRRK DGTTIDWAAN IRKSWDFSED 446**

**ENSBSLP00000028581_Betta_splendens 353:P-ASVLGMAL DPPASLPAAA QWPQGVSLDT LGLAQMPRRK DGTVINWAAD IQKCWDFSEE 411**

**ENSATEP00000003870_Anabas_testudineus 368:P-ASPLGVTL DPPVSLPTPA HWPQGVSLDT LGLANMPRRK DGTVINWAAN IQKSWDFSEE 426**

**ENSMMDP00005031819_Myripristis_murdjan 410:P-GSGLGAPL DAPLSLPTPT HWPQGVCLDR LGLARMPRRK DGTTIDWAAN IRSSWDFSET 468**

**ENSONIP00000067431_Oreochromis_niloticus 383:P-GTAVGVPL DPPVSLPTPA HWPKGVCLDE LGLARMPRRK DGTMVDWAVN IRKTWDFSEG 441**

**ENSNBRP00000029628_Neolamprologus_brichardi 383:P-GTAVGVPL DPPVSLPTPA HWPKGVCLDE LGLARMPRRK DGTTVDWAVN IRKTWDFSEG 441**

**ENSHBUP00000030531_Haplochromis_burtoni 352:P-GTAVGVPL DPPVSLPTPA HWPKGLCLDE LGLARMPRRK DGTTVDWAVN IRKTWDFSEG 410**

**ENSPNYP00000010231_Pundamilia_nyererei 386:P-GTAVGVPL DPPVSLPTPA HWPKGVCLDE LGLARMPRRK DGTTVDWAVN IRKTWDFSEG 444**

**ENSMZEP00005026771_Maylandia_zebra 371:P-GTAVGVPL DPPVSLPTPA HWPKGLCLDE LGLARMPRRK DGTTVDWAAN IRKTWDFSEG 429**

**ENSACLP00000035664_Astatotilapia_calliptera 371:P-GTAVGVPL DPPVSLPTPA HWPKGLCLDE LGLARMPRRK DGTTVDWAAN IRKTWDFSEG 429**

**ENSLBEP00000024994_Labrus_bergylta 346:P-GSALGVPL DPPVSLPTPA QWPQGVSLDG LGLARMPRRK DGTTIDWAAN IRKSWDFTEK 404**

**ENSCLMP00005004807_Cyclopterus_lumpus 405:P-GSALGGPL DPPVSLPTPA HWPQGVSLGT LGLARMPRRK DGTTIDWAAN IRNSWDFSEE 463**

**ENSCGOP00000000746_Cottoperca_gobio 413:P-GSALGVPL DPPVFLPKPA NWPQGVSLGT LGLARMPRRK DGTTIDWAAN IRNSWDFSEE 471**

**ENSSPAP00000013334_Stegastes_partitus 380:P-ASTLGVPL DPPVSLPTPS RWPQGAPLDT LGLARMPRRK DGTTIDWAAN IRKSWDFSEE 438**

**ENSAPOP00000010991_Acanthochromis_polyacanthus 409:P-SSTLGVPL DPPVSLPSPS CWPQGAPLDT LGLARMPRRK DGTTIDWAAN IRTSWDFSED 467**

**ENSAPEP00000019384_Amphiprion_percula 410:P-SSTLGVPL DPPVSLPTPS HWPQGAPLDT LGLAHMPRRK DGTTIDWAAN IRTSWDFSEE 468**

**ENSAOCP00000003852_Amphiprion_ocellaris 379:P-SSTLGVPL DPPVSLPTPS HWPQGAPLDT LGLARMPRRK DGTTIDWAAN IRTSWDFSEE 437**

**ENSLCRP00005003692_Larimichthys_crocea 396:P-GSALGVPL DPPVSLPTPV PWPQGVPLDT LGLARMPCRK DGTTIDWAAN IRESWDFSEE 454**

**ENSSMAP00000005463_Scophthalmus_maximus 421:P-GSALGVPL EPPGSLPTPA HWPQGVCLDN LELARMPRRK DGTMVDWAFN IRKSWDFSEE 479**

**ENSSLDP00000025094_Seriola_lalandi_dorsalis 428:P-GSALGVPL DSPASLPTPA HWPQGVSLDT LGLARMPRRK DGTTIDWAAN IRKSWDFSEE 486**

**ENSLCAP00010022815_Lates_calcarifer 412:P-GSALGVPL DPPVSLPTPA HWPRGVSLDT LGLARMPRRK DGTTIDWAAN IRKCWDFSEE 470**

**ENSSAUP00010027448_Sparus_aurata 422:P-GSALGAPL DPPGSLPAPS NWPQGVSLDT LGLARMPRRK DGTTIDWAAN IRKAWDFSEE 480**

**ENSDLAP00005056549_Dicentrarchus_labrax 412:P-GPALGVPM DPPVSLPTPG HWPQVVSLDT LSLARMPRRK DGTTIDWAAN IRKAWDFSEE 470**

**(Figure S3: 11/19)**

**EKC36382.1_Crassostrea_gigas 374:GAWKALELFL SEGVRKYEKE SCRADHLNTC RISPYLHFGQ ISPRAVLEEA R--HMKSPKF 431**

**ENSLLEP00000020779_Leptobrachium_leishanense 460:GAFRCLGDFL EDGIKHYEKE SGRADKPYTS HISPYLHFGQ ISPRTVLHEA HFTKKSVPKF 519**

**XtCRY6_LC705158 (This_Study) 454:GAYTCLANFL QDGVKHYEKE SGRADKPYTS HISPYLHFGQ ISPRTVLHEA YFTKKNVPKF 513**

**ENSXLAT00005054664_Xenopus_laevis 446:GAYKCLSNFL EDGVKHYEKE SGRADKPYTS HISPYLHFGQ ISPRTVLHEA YFTKKNVPKF 505**

**ENSLOCP00000004832_Lepisosteus_oculatus 427:GAHARLQDFL CDDIWHYPVF VRKLDSSNPE ICSSLKVVIN IKFKHVTLKS KTCNANKGK- 485**

**ENSSFOP00015030596_Scleropages_formosus 411:GAQARLRAFL LDGIHRYEKE SCRADAPNTS CLSPYLHFGQ ISPRLLLWAS RSATCRPPKF 470**

**ENSCHAP00000044642_Clupea_harengus 442:GAHARLEAFL QDGVRRYEKE SARADAPNTS CLSPYLHWGQ LSVRWLLWDA RGARCRSPKF 501**

**ENSAMXP00000003765_Astyanax_mexicanus 400:GAQARLETFL KDGVYRYEKE SCRADVPNTS CLSPYLHFGQ LSVRSLLWDA RDARCRPQKF 459**

**ENSDARP00000105169_Danio_rerio 408:GAHTHLEAFL RDGVYRYEKE SCRADAPNTS CLSPYLHFGQ LSARQVLWAA RGARCKSPKF 467**

**ENSSGRP00000063675_Sinocyclocheilus_grahami 409:GAQAHLEAFL RDGVYRYEKE SSRADEPNTS SLSPYLHFGQ LSARSLLRDA RGARCRSPKF 468**

**ENSCARP00000075848_Carassius_auratus/1801 402:GAQAHLEAFL RDGVYRYEKE SSRADEPNTS SLSPYLHFGQ LSARSLLWDA RGAHCRPPKF 461**

**ENSCARP00000074480_Carassius_auratus/1814 409:GAQAHLEAFL RDGVYRYEKE SSRADEPNTS SLSPYLHFGQ LSGRSLLWDA RGAHCRPPKF 468**

**ENSGMOP00000042713_Gadus_morhua 493:GAHARLEAFL QDGVYRYEKE SGRADSPNTS CLSPYLHWGQ ISPRWLLWDA KSARCRPQKF 552**

**ENSNFUP00015037037_Nothobranchius_furzeri 470:GAHARLDSFL NDGVYRYEQE SGRADASNTS GLSPYLHFGQ LSPRWLLWDA KGACCRSPKF 529**

**ENSTRUP00000037131_Takifugu_rubripes 457:GAHARLEAFL HDGVYRYEKE SGRADAPNTS CVSPYLHFGQ LSPRWLLWDA KGARCRPPKF 516**

**ENSKMAP00000018790_Kryptolebias_marmoratus 460:GAQARLDAFL RDGVYRYESE SGRADAPNTS SLSPYLHFGQ LSPRWLLWDA KGARCRPLKF 519**

**ENSCVAP00000003000_Cyprinodon_variegatus 401:GAHARLEAFL HDGVYRYEKE SGRADAPNTS SLSPYLHFGQ LSPRWLLWDA KGARCRPPKF 460**

**ENSPFOP00000018725_Poecilia_formosa 469:GAHAQLEAFL HDGVYKYEKE SGRADAPNTS CLSPYLHFGQ LSPRWLLWDA KGAKCRPPKF 528**

**ENSFHEP00000001658_Fundulus_heteroclitus 470:GAHARLEAFL HDGVYRYEKE SGRADAPNTS SLSPYLHFGQ LSPRWLLWDA KGARCRPPKF 529**

**ENSOMYP00000024389_Oncorhynchus_mykiss 376:GAQSRLEAFL NDGVYRYEKE SGRADAPNTS CLSPYLHFGQ LSARWLLWDT KGARCRSPKF 435**

**ENSOTSP00005065052_Oncorhynchus_tshawytscha 498:GAQSCLEAFL NDGVYRYEKE SGRADAPNTS CLSPYLHFGQ LSARWLLWDT KAARCRSPKF 557**

**ENSOKIP00005070961_Oncorhynchus_kisutch 497:GAQSRLEAFL NDGVYRYEKE SGRADAPNTS CLSPYLHFGQ LSARWLLWDT KGARCRSPKF 556**

**ENSHHUP00000077840_Hucho_hucho 495:GAQSRLEAFL NDGVYRYEKE SGRADAPNTS CLSPYLHFGQ LSARWLLWDT KGARCRPPKF 554**

**ENSSSAP00000016302_Salmo_salar 441:GAQSRLEAFL NDGVYRYEKE SGRADAPNTS CLSPYLHFGQ LSARWLLWDT KGARCRPPKF 500**

**ENSSTUP00000081428_Salmo_trutta 501:GAQSRLEAFL NDGVYRYEKE SGRADVPNTS CLSPYLHFGQ LSARWLLWDT KGARCRPPKF 560**

**ENSHCOP00000008781_Hippocampus_comes 382:GAHARLEAFL HDGVYRYDKE SGRADAPNTS TLSPYLHFGQ LSPRWLLWDA KGARCRPAKF 441**

**ENSOJAP00000007613_Oryzias_javanicus 439:GAHARLDDFL RDGVYRYEKE SGRADAPNTS SLSPYLHFGQ LSPRWLLWDA KGARCRSPKF 498**

**ENSORLP00000011698_Oryzias_latipes 447:GAHARLDNFL RDGVYRYEKE SGRADAPNTS SLSPYLHFGQ LSPRWLLWDA KGARCRSPKF 506**

**ENSOSIP00000024525_Oryzias_sinensis 447:GAHARLDNFL RDGVYRYEKE SGRADAPNTS SLSPYLHFGQ LSPRWLLWDA KGARCRSPKF 506**

**ENSBSLP00000028581_Betta_splendens 412:GAQARLEAFL QDGVYRYHKE SGRADAPNTS CLSPYLHFGQ LSPRWVLQAA KRTCCQTSKF 471**

**ENSATEP00000003870_Anabas_testudineus 427:GAQAHLEAFL QDGVYRYDKE SGRADAPNTS CLSPYLHFGQ LSPRWVLQAA RGARCQPLKF 486**

**ENSMMDP00005031819_Myripristis_murdjan 469:GAHARLEAFL QDGVYRYEKE SGRADAPNTS CLSPYLHFGQ LSPRWLLWDA KGARCRPPKF 528**

**ENSONIP00000067431_Oreochromis_niloticus 442:GAQARLEAFL HDGVYRYEKE SGRADAPNTS CLSPYLHFGQ LSPRWLMWDA KGARCRPLKF 501**

**ENSNBRP00000029628_Neolamprologus_brichardi 442:GAQARLEAFL HDGVYRYEKE SGRADAPNTS CLSPYLHFGQ LSPRWLMWDA KGARCRPLKF 501**

**ENSHBUP00000030531_Haplochromis_burtoni 411:GAQARLEAFL HDGVYRYEKE SGRADAPNTS CLSPYLHFGQ LSPRWLMWDA KGARCRPLKF 470**

**ENSPNYP00000010231_Pundamilia_nyererei 445:GAQARLEAFL HDGVYRYEKE SGRADAPNTS CLSPYLHFGQ LSPRWLMWDA KGARCRPLKF 504**

**ENSMZEP00005026771_Maylandia_zebra 430:GAQARLEAFL HDGVYRYEKE SGRADAPNTS CLSPYLHFGQ LSPRWLMWDA KGARCRPLKF 489**

**ENSACLP00000035664_Astatotilapia_calliptera 430:GAQARLEAFL HDGVYRYEKE SGRADAPNTS CLSPYLHFGQ LSPRWLMWDA KGARCRPLKF 489**

**ENSLBEP00000024994_Labrus_bergylta 405:GAHARLEAFL QDGVYRYEKD SGRADAPNTS CLSPYLHFGQ LSPRWLLWDA KGARCRPPKF 464**

**ENSCLMP00005004807_Cyclopterus_lumpus 464:GACAQLEAFL NDGVYRYEKE SSRADAPNTS CLSPYLHFGQ LSPRWLLWDA KGARCRPPKF 523**

**ENSCGOP00000000746_Cottoperca_gobio 472:GACARLEAFL NDGVYRYEKE SGRADAPNTS CLSPYLHFGQ LSPRWLLWDA KGARCRPPKF 531**

**ENSSPAP00000013334_Stegastes_partitus 439:GAHARLDAFL HDGVYRYEKE SGRADAPNTS CLSPYLHFGQ LSPRWLLWDA KGARCRPPKF 498**

**ENSAPOP00000010991_Acanthochromis_polyacanthus 468:GAHARLDAFL HDGVYRYEKE SGRADAPNTS SLSPYLHFGQ LSPRWLLWDA KGARCRPPKF 527**

**ENSAPEP00000019384_Amphiprion_percula 469:GAHARLDAFL HDGVYRYEKE SGRADAPNTS SLSPYLHFGQ LSPRWLLWDA KGARCRPPKF 528**

**ENSAOCP00000003852_Amphiprion_ocellaris 438:GAHARLDAFL HDGVYRYEKE SGRADAPNTS SLSPYLHFGQ LSPRWLLWDA KGARCRPPKF 497**

**ENSLCRP00005003692_Larimichthys_crocea 455:GAHARLEAFL HDGVHRYDKE SGRADAPNTS SLSPYLHFGQ LSPRWLLWDS KGARCRCPKF 514**

**ENSSMAP00000005463_Scophthalmus_maximus 480:GAHARLEAFL HDGVYRYEKE SGRADAPNTS SLSPYLHFGQ LSPRWLLWDA KGARCRPPKF 539**

**ENSSLDP00000025094_Seriola_lalandi_dorsalis 487:GAQAQLEAFL HDGVYRYEKE SGRADAPNTS CLSPYLHFGQ LSPRWLLWDA KGARCRPPKF 546**

**ENSLCAP00010022815_Lates_calcarifer 471:GAHARLEAFL HDGVYRYEKE SGRADAPNTS SLSPYLHFGQ LSPRWLLWDA KGARCRPPKF 530**

**ENSSAUP00010027448_Sparus_aurata 481:GAHARLEAFL QDGVYRYEKE SGRADAPNTS SLSPYLHFGQ LSPRWLLWDA KGARCRPPKF 540**

**ENSDLAP00005056549_Dicentrarchus_labrax 471:GAHAQLEAFL QDGVYRYEKE SGRADAPNTS CLSPYLHFGQ LSPRWLLWDA KGARCRPPKF 530**

**(Figure S3: 12/19)**

**EKC36382.1_Crassostrea_gigas 432:LRKLAWRDLS YWLLTLWPDL PSQPTRVHYR DQAWSRDPGH LKAWQRGRTG FPLVDAAMRQ 491**

**ENSLLEP00000020779_Leptobrachium_leishanense 520:LRKLAWRDLA YWLLVLFPEM STEPIRPAYK SQRWSANQKH LQAWQKGMTG YPLVDAAMRE 579**

**XtCRY6_LC705158 (This_Study) 514:LRKLAWRDLA YWLLILFPDM PSEPVRPAYK SQRWSSDLNH LRAWQKGLTG YPLVDAAMRE 573**

**ENSXLAT00005054664_Xenopus_laevis 506:LRKLAWRDLA YWLLLLFPDM PSEPVRPAYK SQRWSSDLNH LRAWQKGLTG YPLVDAAMRE 565**

**ENSLOCP00000004832_Lepisosteus_oculatus 486:NIKLLWTDSV N-----HPGP PVAMPLLPSN AIRWSADRAH LKAWQRGRTG YPLVDAAMRQ 540**

**ENSSFOP00015030596_Scleropages_formosus 471:RRKLAWRDLA YWQLCLFPDL PWQSLRPAYR ALRWSSDRTH LKAWQKGRTG YPLVDAAMRQ 530**

**ENSCHAP00000044642_Clupea_harengus 502:QRKLAWRDLA YWQLTLFPDL PWESLRPPYK ALRWSSERAH LHAWQRGRTG YPLVDAAMRQ 561**

**ENSAMXP00000003765_Astyanax_mexicanus 460:QRKFAWRDLA YWQLCLFPDL PWESLRPPYK ALPWNSDRAH LKAWQRGRTG YPLVDAAMRQ 519**

**ENSDARP00000105169_Danio_rerio 468:QRKLAWRDLA YWQISLFPDL PWESLRPPYK ALRWSSDHAH LKAWQRGRTG YPLVDAAMRQ 527**

**ENSSGRP00000063675_Sinocyclocheilus_grahami 469:QRKLAWRDLA YWQLCLFPDL PWESLRPPYK ALRWSSDRVH LKAWQRGYTG YPLVDAAMRQ 528**

**ENSCARP00000075848_Carassius_auratus/1801 462:QRKLAWRDLA YWQLCLFPDL PWESLRPPYK TMSI------ LKAWQRGSTG YPLIDAAMRQ 515**

**ENSCARP00000074480_Carassius_auratus/1814 469:QRKLAWRDLA YWQLCLFPDL PWESLRPPYK ALRWSSDHVH LKAWQRGSTG YPLIDAAMRQ 528**

**ENSGMOP00000042713_Gadus_morhua 553:QRKLAWRDLA YWQLSLFPAL PWESLRPAYK ALRWSSDRGH LKAWQRGRTG YPLVDAAMRQ 612**

**ENSNFUP00015037037_Nothobranchius_furzeri 530:QRKLAWRDMA YWQLTLFPDL PWESLRPPYK ALRWSTDRGH LKAWQQGKTG YPLVDAAMRQ 589**

**ENSTRUP00000037131_Takifugu_rubripes 517:QRKLAWRDLA YWQLTLFPDL PWESLRPPYK ALRWSTDRRH LEAWQRGRTG YPLVDAAMRQ 576**

**ENSKMAP00000018790_Kryptolebias_marmoratus 520:QRKLAWRDLA YWQLSLFPDL PWESLRPPYK ALRWSADRSH LKAWQQGKTG YPLVDAAMRQ 579**

**ENSCVAP00000003000_Cyprinodon_variegatus 461:QRKLAWRDLA YWQLTLFPDL PWESLRPPYK ALRWNTDRSH LKAWQKGNTG YPLVDAAMRQ 520**

**ENSPFOP00000018725_Poecilia_formosa 529:QRKLAWRDLA YWQLTLFPDL PWESLRPPYK ALRWSTDCHH LKAWQKGKTG YPLVDAAMRQ 588**

**ENSFHEP00000001658_Fundulus_heteroclitus 530:QRKLAWRDLA YWQLTLFPDL PWESLRPPYK ALRWSTNRSH LKAWQKGKTG YPLVDAAMRQ 589**

**ENSOMYP00000024389_Oncorhynchus_mykiss 436:IRKLAWRDLA YWQLTLFPDL PWESLRPPYK ALRWSNERGH LKAWQKGRTG YPLVDAAMRQ 495**

**ENSOTSP00005065052_Oncorhynchus_tshawytscha 558:IRKLAWRDLA YWQLTLFPDL PWESLRPPYK ALRWSNERGH LKAWQKGRTG YPLVDAAMRQ 617**

**ENSOKIP00005070961_Oncorhynchus_kisutch 557:IRKLTWRDLA YWQLTLFPDL PWESLRPPYK ALRWSNERGH LKAWQKGRTG YPLVDAAMRQ 616**

**ENSHHUP00000077840_Hucho_hucho 555:IRKLAWRDLA YWQLTLFPDL PWESLRPPYK ALRWSNERGH LKAWQKGRTG YPLVDAAMRQ 614**

**ENSSSAP00000016302_Salmo_salar 501:IRKLAWRDLA YWQLTLFPDL PWESLRPPYK ALRWSNERGH LKAWQKGRTG YPLVDAAMRQ 560**

**ENSSTUP00000081428_Salmo_trutta 561:IRKLAWRDLA YWQLTLFPDL PWESLRPPYK ALRWSNERGH LKAWQKGRTG YPLVDAAMRQ 620**

**ENSHCOP00000008781_Hippocampus_comes 442:QRKLAWRDLA YWQLTLFPDL PWESLRPPYK ELRWSSNRNH LKAWQRGRTG YPLVDAAMRQ 501**

**ENSOJAP00000007613_Oryzias_javanicus 499:QRKLAWRDLA YWQLTLFPDL PWESIRPPYK ALRWNTDRSH LKAWQRGKTG YPLVDAAMRQ 558**

**ENSORLP00000011698_Oryzias_latipes 507:QRKLAWRDLA YWQLTLFPDL PWESIRPPYK ALRWNTDRSH LKAWQRGKTG YPLVDAAMRQ 566**

**ENSOSIP00000024525_Oryzias_sinensis 507:QRKLAWRDLA YWQLTLFPDL PWESIRPPYK ALRWNTDRSH LKAWQRGKTG YPLVDAAMRQ 566**

**ENSBSLP00000028581_Betta_splendens 472:QRKLAWRDLA YWQLTLFPDL PWESVRPPYK ALCWSSRRDH LKAWQRGRTG YPLVDAAMRQ 531**

**ENSATEP00000003870_Anabas_testudineus 487:QRKLAWRDLA YWQLTLFPDL PWESVRPPYK ALQWSSNRGH LKAWQRGLTG YPLVDAAMRQ 546**

**ENSMMDP00005031819_Myripristis_murdjan 529:QRKLAWRDLA YWQLTLFPDL PWESLRPPYK ALRWSSVRGH LKAWQRGRTG YPLVDAAMRQ 588**

**ENSONIP00000067431_Oreochromis_niloticus 502:QRKLAWRDLA YWQLTLFPDL PWESIRPPYK ALRWSSDRGH LKAWQRGRTG YPLVDAAMRQ 561**

**ENSNBRP00000029628_Neolamprologus_brichardi 502:QRKLAWRDLA YWQLTLFPDL PWESIRPPYK ALRWSSDRGH LKAWQRGRTG YPLVDAAMRQ 561**

**ENSHBUP00000030531_Haplochromis_burtoni 471:QRKLAWRDLA YWQLTLFPDL PWESIRPPYK ALRWSSDRGH LKAWQRGRTG YPLVDAAMRQ 530**

**ENSPNYP00000010231_Pundamilia_nyererei 505:QRKLAWRDLA YWQLTLFPDL PWESIRPPYK ALRWSSDWGH LKAWQRGRTG YPLVDAAMRQ 564**

**ENSMZEP00005026771_Maylandia_zebra 490:QRKLAWRDLA YWQLTLFPDL PWESIRPPYK ALRWSSDRGH LKAWQRGRTG YPLVDAAMKQ 549**

**ENSACLP00000035664_Astatotilapia_calliptera 490:QRKLAWRDLA YWQLTLFPDL PWESIRPPYK ALRWSSDRGH LKAWQRGRTG YPLVDAAMKQ 549**

**ENSLBEP00000024994_Labrus_bergylta 465:QRKLAWRDLA YWQLTLFPDL PWESLRPPYK ALRWSVDHGH LKAWQRGRTG YPLVDAAMRQ 524**

**ENSCLMP00005004807_Cyclopterus_lumpus 524:QRKLAWRDLA YWQLTLFPDL PWESLRPPYK ALRWSSDRGH LKAWQRGQTG YPLVDAAMRQ 583**

**ENSCGOP00000000746_Cottoperca_gobio 532:QRKLAWRDLA YWQLTLFPDL PWESLRPPYK ALRWSSDRGH LKAWQRGRTG YPLVDAAMRQ 591**

**ENSSPAP00000013334_Stegastes_partitus 499:QRKLAWRDLA YWQLTLFPDL PWESLRPPYK ALRWSSDRGH LKAWQRGKTG YPLVDAAMRQ 558**

**ENSAPOP00000010991_Acanthochromis_polyacanthus 528:QRKLAWRDLA YWQLTLFPDL PWESLRPPYK ALRWSSDRSH LKAWQRGKTG YPLVDAAMRQ 587**

**ENSAPEP00000019384_Amphiprion_percula 529:QRKLAWRDLA YWQLTLFPDL PWESLRPPYK ALRWSSDRSH LKAWQRGKTG YPLVDAAMRQ 588**

**ENSAOCP00000003852_Amphiprion_ocellaris 498:QRKLAWRDLA YWQLTLFPDL PWESLRPPYK ALRWSSDRSH LKAWQRGKTG YPLVDAAMRQ 557**

**ENSLCRP00005003692_Larimichthys_crocea 515:QRKLAWRDLA YWQLTLFPGL PWDSIRPPYK ALRWSTDRSH LKAWQQGQTG YPLVDAAMRQ 574**

**ENSSMAP00000005463_Scophthalmus_maximus 540:QRKLAWRDLA YWQLTLFPDL PWESLRPPYK NLRWSNDRGH LKAWQRGRTG YPLVDAAMRQ 599**

**ENSSLDP00000025094_Seriola_lalandi_dorsalis 547:QRKLAWRDLA YWQLTLFPDL PWESLRPPYK ALRWSSDRGH LKAWQRGQTG YPLVDAAMRQ 606**

**ENSLCAP00010022815_Lates_calcarifer 531:QRKLAWRDLA YWQLTLFPDL PWESLRPPYK ALRWSSDRGQ LKAWQRGRTG YPLVDAAMRQ 590**

**ENSSAUP00010027448_Sparus_aurata 541:QRKLAWRDLA YWQLTLFPDL PWESLRPPYK ALRWSSDRGH LKAWQRGRTG YPLVDAAMRQ 600**

**ENSDLAP00005056549_Dicentrarchus_labrax 531:QRKLAWRDLA YWQLILFPDL PWESLRPPYK ALRWSRDRGH LKAWQRGQTG YPLVDAAMRQ 590**

**(Figure S3: 13/19)**

**EKC36382.1_Crassostrea_gigas 492:LWLEGWINNY LRHVVASFLI SYLRLHWVEG YRWFQDTLLD ADVAINAMMW QNGGMSGLDQ 551**

**ENSLLEP00000020779_Leptobrachium_leishanense 580:LWLTGWMSNY SRHVVASFLV AYLHIHWVHG YRWFQDTLVD ADVAINAMMW QNGGMSGLDH 639**

**XtCRY6_LC705158 (This_Study) 574:LWLTGWMCNY SRHVVASFLV AYLHIHWVHG YRWFQDTLLD ADVAINAMMW QNGGMSGLDH 633**

**ENSXLAT00005054664_Xenopus_laevis 566:LWLTGWMCNY SRHVVASFLV AYLHIHWVHG YRWFQDTLLD ADVAINAMMW QNGGMSGLDH 625**

**ENSLOCP00000004832_Lepisosteus_oculatus 541:LWLTGWMNNY MRHVVASFLI AYLHLPWQEG YRWFQDTLVD ADVAIDAVMW QNGGMCGLDH 600**

**ENSSFOP00015030596_Scleropages_formosus 531:LWLTGWMNNY MRHVVASFLI AYLHLPWQEG YRWFQDTLVD ADVAIDAMMW QNGGMCGLDH 590**

**ENSCHAP00000044642_Clupea_harengus 562:LWQTGWMNNY MRHVVASFLI AYLHLPWQEG YRWFQDTLVD ADVAIDAMMW QNGGMSGLDH 621**

**ENSAMXP00000003765_Astyanax_mexicanus 520:LWLTGWMNNY MRHVAASFLI AYLHIAWQEG YRWFQDTLVD ADVAIDAMMW QNGGMCGLDH 579**

**ENSDARP00000105169_Danio_rerio 528:LWQTGWMNNY MRHVVASFLI AYLHFPWQEG YRWFQDTLVD ADVAIDAMMW QNGGMCGLDH 587**

**ENSSGRP00000063675_Sinocyclocheilus_grahami 529:LWQTGWMNNY MRHVVASFLI SYLHIAWQEG YRWFQDTLVD ADVAIDAMMW QNGGMCGLDH 588**

**ENSCARP00000075848_Carassius_auratus/1801 516:LWQTGWMNNY MRHVVASFLI AYLHIAWQEG YRWFQDTLVD ADVAIDAMMW QNGGMCGLDH 575**

**ENSCARP00000074480_Carassius_auratus/1814 529:LWQTGWMNNY MRHVVASFLI AYLHIAWQEG YRWFQDTLVD ADVAIDAMMW QNGGMCGLDH 588**

**ENSGMOP00000042713_Gadus_morhua 613:LWLTGWVNNY MRHVVASFLI AYLHLPWQEG YLWFQDTLLD ADVAIDAMMW QNGGMCGLDH 672**

**ENSNFUP00015037037_Nothobranchius_furzeri 590:LWQTGWMNNY MRHVVASFLI AYLHLPWQEG YRWFQDTLVD ADVAIDAMMW QNGGMCGLDH 649**

**ENSTRUP00000037131_Takifugu_rubripes 577:LWLTGWMNNY MRHVVASFLI AYLHLPWQEG YRWFQDTLVD ADVAIDAMMW QNGGMCGLDH 636**

**ENSKMAP00000018790_Kryptolebias_marmoratus 580:LWETGWMNNY MRHVVASFLI AYLYLPWQEG YRWFQDTLVD ADVAIDAMMW QNGGMSGLDH 639**

**ENSCVAP00000003000_Cyprinodon_variegatus 521:LWLTGWMNNY MRHVVASFLI AYLHLPWQEG YRWFQDTLVD ADVAIDAMMW QNGGMCGLDH 580**

**ENSPFOP00000018725_Poecilia_formosa 589:LWLTGWMNNY MRHVVASFLI AYLHLPWQEG YRWFQDTLVD ADVAIDAMMW QNGGMCGLDH 648**

**ENSFHEP00000001658_Fundulus_heteroclitus 590:LWLTGWMNNY MRHVVASFLI AYLHLPWQEG YRWFQDTLVD ADVAIDAMMW QNGGMCGLDH 649**

**ENSOMYP00000024389_Oncorhynchus_mykiss 496:LWLTGWMNNY MRHVVASFLI AYLHLPWQEG YRWFQDTLVD ADVAIDAMMW QNGGMCGLDH 555**

**ENSOTSP00005065052_Oncorhynchus_tshawytscha 618:LWLTGWMNNY MRRVVASFLI AYLHLPWQEG YRWFQDTLVD ADVAIDAMMW QNGGMCGLDH 677**

**ENSOKIP00005070961_Oncorhynchus_kisutch 617:LWLTGWMNNY MRHVVASFLI AYLHLPWQEG YRWFQDTLVD ADVAIDAMMW QNGGMCGLDH 676**

**ENSHHUP00000077840_Hucho_hucho 615:LWLTGWMNNY MRHVVASFLI AYLHLPWQEG YRWFQDTLVD ADVAIDAMMW QNGGMCGLDH 674**

**ENSSSAP00000016302_Salmo_salar 561:LWLTGWMNNY MRHVVASFLI AYLHLPWQEG YRWFQDTLVD ADVAIDAMMW QNGGMCGLDH 620**

**ENSSTUP00000081428_Salmo_trutta 621:LWLTGWMNNY MRHVVASFLI AYLHLPWQEG YRWFQDTLVD ADVAIDAMMW QNGGMCGLDH 680**

**ENSHCOP00000008781_Hippocampus_comes 502:LWLTGWMNNY TRHVVASFLI AYLHLPWQEG YRWFQDTLVD ADVAIDAMMW QNGGMCGLDH 561**

**ENSOJAP00000007613_Oryzias_javanicus 559:LWLTGWMNNY TRHVVASFLI AYLHLPWQEG YRWFQDTLVD ADVAIDAMMW QNGGMCGLDH 618**

**ENSORLP00000011698_Oryzias_latipes 567:LWLTGWMNNY MRHVVASFLI AYLHLPWQEG YRWFQDTLVD ADVAIDAMMW QNGGMCGLDH 626**

**ENSOSIP00000024525_Oryzias_sinensis 567:LWLTGWMNNY MRHVVASFLI AYLHLPWQEG YRWFQDTLVD ADVAIDAMMW QNGGMCGLDH 626**

**ENSBSLP00000028581_Betta_splendens 532:LWLTGWMSNY MRHVVASFLI AYLHLPWQQG YRWFQDTLVD ADVAIDAMMW QNGGMCGLDH 591**

**ENSATEP00000003870_Anabas_testudineus 547:LWLTGWMNNY MRHVVASFLI AYLHLPWQEG YRWFQDTLVD ADVAIDAMMW QNGGMCGLDH 606**

**ENSMMDP00005031819_Myripristis_murdjan 589:LWLTGWMNNY MRHVVASFLI AYLHLPWQEG YRWFQDTLVD ADVAIDAMMW QNGGMCGLDH 648**

**ENSONIP00000067431_Oreochromis_niloticus 562:LWQTGWMNNY MRHVVASFLI AYLHLPWQEG YRWFQDTLVD ADVAIDAMMW QNGGMCGLDH 621**

**ENSNBRP00000029628_Neolamprologus_brichardi 562:LWQTGWMNNY MRHVVASFLI AYLHLPWQEG YRWFQDTLVD ADVAIDAMMW QNGGMCGLDH 621**

**ENSHBUP00000030531_Haplochromis_burtoni 531:LWQTGWMNNY MRHVVASFLI AYLHLPWQEG YRWFQDTLVD ADVAIDAMMW QNGGMCGLDH 590**

**ENSPNYP00000010231_Pundamilia_nyererei 565:LWQTGWMNNY MRHVVASFLI AYLYLPWQEG YRWFQDTLVD ADVAIDAMMW QNGGMCGLDH 624**

**ENSMZEP00005026771_Maylandia_zebra 550:LWQTGWMNNY MRHVVASFLI AYLYLPWQEG YRWFQDTLVD ADVAIDAMMW QNGGMCGLDH 609**

**ENSACLP00000035664_Astatotilapia_calliptera 550:LWQTGWMNNY MRHVVASFLI AYLYLPWQEG YRWFQDTLVD ADVAIDAMMW QNGGMCGLDH 609**

**ENSLBEP00000024994_Labrus_bergylta 525:LWLTGWMNNY MRHVVASFLI AYLYLPWQEG YRWFQDTLVD ADVAIDAMMW QNGGMCGLDH 584**

**ENSCLMP00005004807_Cyclopterus_lumpus 584:LWLTGWMNNY MRHVVASFLI AYLYLPWQEG YRWFQDTLVD ADVAIDAMMW QNGGMCGLDH 643**

**ENSCGOP00000000746_Cottoperca_gobio 592:LWLTGWMNNY MRHVVASFLI AYLYLPWQEG YRWFQDTLVD ADVAIDAMMW QNGGMC---- 647**

**ENSSPAP00000013334_Stegastes_partitus 559:LWLTGWMNNY MRHVVASFLI AYLHLPWQEG YRWFQDTLVD ADVAIDAMMW QNGGMCGLDH 618**

**ENSAPOP00000010991_Acanthochromis_polyacanthus 588:LWLTGWMNNY MRHVVASFLI AYLHLPWQEG YRWFQDTLVD ADVAIDAMMW QNGGMCGLDH 647**

**ENSAPEP00000019384_Amphiprion_percula 589:LWLTGWMNNY MRHVVASFLI AYLHLPWQEG YRWFQDTLVD ADVAIDAMMW QNGGMCGLDH 648**

**ENSAOCP00000003852_Amphiprion_ocellaris 558:LWLTGWMNNY MRHVVASFLI AYLHLPWQEG YRWFQDTLVD ADVAIDAMMW QNGGMCGLDH 617**

**ENSLCRP00005003692_Larimichthys_crocea 575:LWLTGWMNNY MRHVVASFLI AYLYLPWQEG YRWFQDTLVD ADVAIDAMMW QNGGMCGLDH 634**

**ENSSMAP00000005463_Scophthalmus_maximus 600:LWLTGWMNNY MRHVVASFLI AYLHLPWQDG YRWFQDTLLD ADVAIDAMMW QNGGMCGLDH 659**

**ENSSLDP00000025094_Seriola_lalandi_dorsalis 607:LWLTGWMNNY MRHVVASFLI AYLHLPWQEG YRWFQDTLVD ADVAIDAMMW QNGGMCGLDH 666**

**ENSLCAP00010022815_Lates_calcarifer 591:LWLTGWMNNY MRHVVASFLI AYLHLPWQEG YRWFQDTLVD ADVAIDAMMW QNGGMCGLDH 650**

**ENSSAUP00010027448_Sparus_aurata 601:LWLTGWMNNY MRHVVASFLI AYLHLPWQEG YRWFQDTLVD ADVAIDAMMW QNGGMCGLDH 660**

**ENSDLAP00005056549_Dicentrarchus_labrax 591:LWLTGWMNNY MRHVVASFLI AYLYLPWQEG YRWFQDTLVD ADVAIDAMMW QNGGMCGLDH 650**

**(Figure S3: 14/19)**

**EKC36382.1_Crassostrea_gigas 552:WNFVMHPVDA ALTCDPDGAY VRKWCPEIAA LPNDFIHQPW KCPPSILRRC GIKLGETYPY 611**

**ENSLLEP00000020779_Leptobrachium_leishanense 640:WNFVMHPVDA ALTCDPYGSY VRKWCPELDG LPDDYIHKPW KCPPAQLRRA GVVLGENYPH 699**

**XtCRY6_LC705158 (This_Study) 634:WNFVMHPVDS ALTCDPYGSY VRKWCPELAG LPDEYIHKPW KCAPSQLRRA GVILGRNYPH 693**

**ENSXLAT00005054664_Xenopus_laevis 626:WNFVMHPVDS ALTCDPYGSY VRKWCPELAG LPDEYIHKPW KCAPSQLRRA GVILGQNYPH 685**

**ENSLOCP00000004832_Lepisosteus_oculatus 601:WNFVMHPVSA ALTCDPYGSF VRKWCPELAA LPDELIHKPW QCPASVLRRA GVTLGGDYPE 660**

**ENSSFOP00015030596_Scleropages_formosus 591:WNFVMHPVSA GLTCDPRGTF ARRWCPELAA LPDELVHRPW KSPASMLRRA GVTLGVNYPE 650**

**ENSCHAP00000044642_Clupea_harengus 622:WNFVMHPVDA AMTCDPNGTY VKRWCPELAP LPDELIHKPW KCPASILRRA GVVLGQNYPE 681**

**ENSAMXP00000003765_Astyanax_mexicanus 580:WNFVMHPVDA ALTCDPNGNY VRQWCPELKG LPDDFIHKPW MCPGSVLRRA GVILGQNYPE 639**

**ENSDARP00000105169_Danio_rerio 588:WNFVMHPIDA ALTCDPCGTF VRQWCPELKA LPDDLIHKPW KCPTSMLRRA GVVFGDSYPE 647**

**ENSSGRP00000063675_Sinocyclocheilus_grahami 589:WNFVMHPVDA ALTCDPYGTY VRQWCPELKA LPNDLIHKPW KCPASMLRRA GVVLGGNYPE 648**

**ENSCARP00000075848_Carassius_auratus/1801 576:WNFVMHPVDA ALTCDPCGTY VRQWCPVLKA LPDDLVHKPW KCPASMLRRA GVVLGGNYPE 635**

**ENSCARP00000074480_Carassius_auratus/1814 589:WNFVMHPVDA ALTCDPYGTY VRQWCPVLKA LPDDLVHKPW KCPASMLRRA GVVLGGNYPE 648**

**ENSGMOP00000042713_Gadus_morhua 673:WNFVMHPVDA ALTCDPGGSY VRKWCPELAA LPDDIIHKPW RCPASILRRA GVAFGHTYPE 732**

**ENSNFUP00015037037_Nothobranchius_furzeri 650:WNFVMHPIDA AMTCDPNGSY VRKWCPELSD LPDELIHKPW KCPASMLRRA GIVFGQTYAE 709**

**ENSTRUP00000037131_Takifugu_rubripes 637:WNFVMHPVDA AMTCDPCGQY VRKWCPELSG LPDELIHKPW RCPTSLLRRA GVVFGQTYPE 696**

**ENSKMAP00000018790_Kryptolebias_marmoratus 640:WNFVMHPVDA AMTCDPYGSY VRKWCPELAD LPDELIHKPW KCPASMLRRA GVVFGQTYPE 699**

**ENSCVAP00000003000_Cyprinodon_variegatus 581:WNFVMHPVDA AMTCDPYGSY VRKWCPELSD LPDELIHKPW KCPASMLRRA GVVFGQTYPE 640**

**ENSPFOP00000018725_Poecilia_formosa 649:WNFVMHPVDA AMTCDPYGSY VRKWCPELAD LPDELIHKPW KCPASMLRRA GVVFGQTYPE 708**

**ENSFHEP00000001658_Fundulus_heteroclitus 650:WNFVMHPIDA AMTCDPYGSY VRKWCPELAD LPDELIHKPW ACPASMLRRA GVAFGQTYPE 709**

**ENSOMYP00000024389_Oncorhynchus_mykiss 556:WNFVMHPVDA AMTCDPYGSY VRKWCPELAV LHDDHIHKPW KCPASMLRRA GVVLGQSYPE 615**

**ENSOTSP00005065052_Oncorhynchus_tshawytscha 678:WNFVMHPVDA AMTCDPYGSY VRKWCSELAV LPDDLIHKPW KCPASMLRRA GVVLGQSYPE 737**

**ENSOKIP00005070961_Oncorhynchus_kisutch 677:WNFVMHPVDA AMTCDPYGSY VRKWCSELAV LPDDLIHKPW KCPASMLRRA GVVLGQSYPE 736**

**ENSHHUP00000077840_Hucho_hucho 675:WNFVMHPVDA AMTCDPYGNY VRKWCPELAV LPDDLIHKPW KCPASMLRRA GVVLGQSYPE 734**

**ENSSSAP00000016302_Salmo_salar 621:WNFVMHPVDA AMTCDPYGNY VRKWCTELAV LPDDLIHKPW KCPASMLRRA GVVLGQSYPE 680**

**ENSSTUP00000081428_Salmo_trutta 681:WNFVMHPVDA AMTCDPYGNY VRKWCTELAV LPDDLIHKPW KCPASMLRRA GVVLGQSYPE 740**

**ENSHCOP00000008781_Hippocampus_comes 562:WNFVMHPVDA AMTCDPNGSY VRKWCPEIAE LPDELIHKPW KCPTSMLRRA GVVLGQTYPE 621**

**ENSOJAP00000007613_Oryzias_javanicus 619:WNFVMHPVDA ALTCDPYGHY VRKWCPELAD LPDELIHKPW KCPASMLRRA RVVFGETYPE 678**

**ENSORLP00000011698_Oryzias_latipes 627:WNFVMHPVDA ALTCDPYGHY VRKWCPELAD LPDELIHKPW KCPASMLRRA KVVFGETFPE 686**

**ENSOSIP00000024525_Oryzias_sinensis 627:WNFVMHPVDA ALTCDPYGHY VRKWCPELAD LSDELIHKPW KCPASMLRRA KVVFGETFPE 686**

**ENSBSLP00000028581_Betta_splendens 592:WNFVMHPVDA AMTCDPCGTY VRKWCPELAD LSDEFIHKPW KCPISMLRRS GVVFGQTYPE 651**

**ENSATEP00000003870_Anabas_testudineus 607:WNFVMHPIDA AMTCDPYGNY VRKWCPELAD LPDELIHKPW KCPSSMLRRA GVVFGQTYPE 666**

**ENSMMDP00005031819_Myripristis_murdjan 649:WNFVMHPVDA AMTCDPYGSY VRKWCPELAE LPDELIHKPW KCPASMLRRA GVVFGQNYPE 708**

**ENSONIP00000067431_Oreochromis_niloticus 622:WNFVMHPVDA AMTCDPYGTY VRKWCPELAD LPDELIHKPW KCPASMLRRA GVVFGQSYPE 681**

**ENSNBRP00000029628_Neolamprologus_brichardi 622:WNFVMHPVDA AMTCDPYGTY VRKWCPELAE LPDELIHKPW KCPASMLRRA GVVFGQSYPE 681**

**ENSHBUP00000030531_Haplochromis_burtoni 591:WNFVMHPVDA AMTCDPYGTY VRKWCPELAE LPDELIHKPW KCPASMLRRA GVVFGQSYPE 650**

**ENSPNYP00000010231_Pundamilia_nyererei 625:WNFVMHPVDA AMTCDPYGTY VRKWCPELAE LPDELIHKPW KCPASMLRRA GVVFGQSYPE 684**

**ENSMZEP00005026771_Maylandia_zebra 610:WNFVMHPVDA AMTCDPYGTY VRKWCPELAE LPDELIHKPW KCPASMLRRA GVVFGQSYPE 669**

**ENSACLP00000035664_Astatotilapia_calliptera 610:WNFVMHPVDA AMTCDPYGTY VRKWCPELAE LPDELIHKPW KCPASMLRRA GVVFGQSYPE 669**

**ENSLBEP00000024994_Labrus_bergylta 585:WNFIMHPVDA AMTCDPCGSY VRTWCPELAD LPEELIHKPW KCPTSMLRRA GVVFGQTYPE 644**

**ENSCLMP00005004807_Cyclopterus_lumpus 644:WNFVMHPVDA AMTCDPYGSH VRKWCPELAD LPDDLIHKPW KCPASMLRRA GVVFGQTYAE 703**

**ENSCGOP00000000746_Cottoperca_gobio 648:---------- AMTCDPYGSY VKKWCPELAD LPDELIHKPW KCPESMLRRA GVVFGQTYPE 697**

**ENSSPAP00000013334_Stegastes_partitus 619:WNFVMHPIDA AMTCDPYGSY VRKWCPELAD LPDELIHKPW KCPASMLRRA GVAFGQTYPE 678**

**ENSAPOP00000010991_Acanthochromis_polyacanthus 648:WNFVMHPIDA AMTCDPYGSY VRKWCPELAD LPDELIHKPW KCPASVLRRA GVVFGQTYPE 707**

**ENSAPEP00000019384_Amphiprion_percula 649:WNFVMHPTDA AMTCDPYGSY VRKWCPELAD IPDELIHKPW KCPASMLRRA GVVFGQTYPE 708**

**ENSAOCP00000003852_Amphiprion_ocellaris 618:WNFVMHPTDA AMTCDPYGSY VRKWCPELAD IPDELIHKPW KCPASMLRRA GVVFGQTYPE 677**

**ENSLCRP00005003692_Larimichthys_crocea 635:WNFVMHPVDA AMTCDPYGSY VRKWCPELAS LPDELIHKPW KCPASMLRRA GVVFGQTYPE 694**

**ENSSMAP00000005463_Scophthalmus_maximus 660:WNFVMHPTDA AMTCDPYGSY VRKWCPELAD LPDELIHKPW KCPASMLRRA GVVFGQTYPD 719**

**ENSSLDP00000025094_Seriola_lalandi_dorsalis 667:WNFVMHPVDA AMTCDPYGSY VRKWCPELAD LPDELIHKPW KCPASMLRRA GVVFGQTYPD 726**

**ENSLCAP00010022815_Lates_calcarifer 651:WNFVMHPVDA AMTCDPYGSY VRKWCPELAD LPDELIHKPW KCPASMLRRA GVVFGQTYPN 710**

**ENSSAUP00010027448_Sparus_aurata 661:WNFVMHPVDA AMTCDPCGSY VRKWCPELAD LPDELIHKPW KCPASMLRRS GVVFGQTYPE 720**

**ENSDLAP00005056549_Dicentrarchus_labrax 651:WNFVMHPVDA AMTCDPYGSY VRKWCPELAD LPDELIHKPW KCPASMLRRA GVVFGQTYPE 710**

**(Figure S3: 15/19)**

**EKC36382.1_Crassostrea_gigas 612:RVISDLEGAR EQSLTDVVNV RKKHP-EFVD RRTGNDLVPL PDGLC----- ---------- 655**

**ENSLLEP00000020779_Leptobrachium_leishanense 700:RIAEDLEERR EQSLKDVVDV RHKHP-EYVD DISGCDMVPI PDSLLASTLA HAG-EDEMVQ 757**

**XtCRY6_LC705158 (This_Study) 694:RIVLDLEERR EQSLKDVVEV RKKHL-EYLD EVSGCDMVQI PDQLLALTLG HTSGEDEVVR 752**

**ENSXLAT00005054664_Xenopus_laevis 686:RIVLDLEERR EQSLKDVVEV RKKHL-EYLD EVSGSDMIPI PDQLLALTLG RPNGDDEVVR 744**

**ENSLOCP00000004832_Lepisosteus_oculatus 661:RIVADLEERR ARSLRDVATA RQQFAGEYVD RRSGCDLLPL PDKLVKEALG GGG--GGEVV 718**

**ENSSFOP00015030596_Scleropages_formosus 651:RIITDLEERR AQSLRDVVRV RRNFP-QYVD QLSGCDLVPL PQRLVQEALG S-----GEVA 704**

**ENSCHAP00000044642_Clupea_harengus 682:RVVTDLEELR TQSLRDVAEV RRRFS-EYVD QHSGCDLIPL PDRLVEEALG ST----GEVL 736**

**ENSAMXP00000003765_Astyanax_mexicanus 640:RVVVELEERR AQSLQDVRMV RKRFS-EYVC QQTGCDLLPL PTKLVQEALG ST---DDLVQ 695**

**ENSDARP00000105169_Danio_rerio 648:RIVIDLEERR AQSLQDVASV RRRFR-QFVD QRSGCDLVPV PSRLVQDALG SM---EDIVK 703**

**ENSSGRP00000063675_Sinocyclocheilus_grahami 649:RIVVDLEERR AQSLQDVASV RRRFR-EFVD QRSGCDLVPV PARLVQEALG SM---EDVVS 704**

**ENSCARP00000075848_Carassius_auratus/1801 636:RIVVDLEERR AQSLQDVATV RRRFR-EFVD QRSGCDLVPV PARLVQDALG SM---EDVVS 691**

**ENSCARP00000074480_Carassius_auratus/1814 649:RIVVDLEERR AQSLQDVATV RRRFR-EFVD QRSGCDLVPV PARLVQDALG SM---EDVVS 704**

**ENSGMOP00000042713_Gadus_morhua 733:PIVTDLEARR GQSLQDVAQV RSTLS-EYVD ERSGCDLVPL PPRLVAEALG SAG--GDQLR 789**

**ENSNFUP00015037037_Nothobranchius_furzeri 710:RVITDLEERR TRSLQDVALV RREFG-QYVD KRSGCDLLPL PPRLVSEALG RSH-SDEAVV 767**

**ENSTRUP00000037131_Takifugu_rubripes 697:RIVTDLEERR SQSLQDVALV RKEFG-QYVD KRSGCDLVPL PPRLVSEALG SSH-LDGGVA 754**

**ENSKMAP00000018790_Kryptolebias_marmoratus 700:RIVTDLEERR SRSLQDVALV RKEFG-QYVD ERSGCDLVPL PPRLVSEALG SSH-GDEAVV 757**

**ENSCVAP00000003000_Cyprinodon_variegatus 641:RIVIDLEERR TKSLQDVALV RKQFQ-QYVD KRSGCDLVPL PKRLVSEALG LSH-RDGAVV 698**

**ENSPFOP00000018725_Poecilia_formosa 709:RIVTNLEEQR SKSLQDVALV RKQFQ-QYVD KCSGCDLVPL PKRLVSEALG LSH-WDGAVV 766**

**ENSFHEP00000001658_Fundulus_heteroclitus 710:RIVTDLEERR SKSLQDVALV RKQFQ-QYVD KRSGCDLVPL PKRLVSKTLG LSH-RDGAVV 767**

**ENSOMYP00000024389_Oncorhynchus_mykiss 616:RVVTDLEERR SQSLQDVALV RRRFG-QYVD PCSGCDLVPL PPRLVSEAMG ------GGMV 668**

**ENSOTSP00005065052_Oncorhynchus_tshawytscha 738:RVVTDLEERR SQSLQDVALV RRRFG-EYVD PCSGCDLVPL PPRLVSEAMG ------GGMV 790**

**ENSOKIP00005070961_Oncorhynchus_kisutch 737:RVVTDLEERR SQSLQDVALV RRRFG-EYVD PCSGCDLVPL PPRLVSEAMG ------GGMV 789**

**ENSHHUP00000077840_Hucho_hucho 735:RVVTDLEERR SQSLQDVALV RRRFG-EYVD PCSGCDLVPL PPRLVSEALG ------GGMV 787**

**ENSSSAP00000016302_Salmo_salar 681:RVVTDLEERR SQSLQDVALV RRRFG-EYVD PCSGCDLVPL PPRLVSETIF ------HIQI 733**

**ENSSTUP00000081428_Salmo_trutta 741:RVVTDLEERR SQSLQDVALV RRRFG-EYVD PCSGCDLVPL PPRLVSEAMG ------GGMV 793**

**ENSHCOP00000008781_Hippocampus_comes 622:RIVTNLEERR SRSLQDVALV RRQHA-EYVD TRSGCDLVPL PPRLVSEALG LAQ-PTG--- 676**

**ENSOJAP00000007613_Oryzias_javanicus 679:RIVVDLEERR NQSLQDVASV RKEFG-QFVD KRSGCDLVPL PPRLVSEALG WSQ-RDGAAA 736**

**ENSORLP00000011698_Oryzias_latipes 687:RIVVDLEERR NQSLQDVASV RKEFG-QFVD KRSGCDLVPL PPRLVAEALG LSQ-RDGAV- 743**

**ENSOSIP00000024525_Oryzias_sinensis 687:QIVVDLEERR NQSLQDVASV RKEFG-EFVD KRSGCDLVPL PPRLVSEALG LSQ-RDGAV- 743**

**ENSBSLP00000028581_Betta_splendens 652:RIITDLEERR SQSLQDVAHV RKEFN-QYVD KHTGCDLVPL PPRLLSETMG LSQ-KDDCVV 709**

**ENSATEP00000003870_Anabas_testudineus 667:RIITDLEERR SQSLQDVARV RREFQ-QYVD KRTGCDLVPL PPSLVTKALG LSH-QDSSVV 724**

**ENSMMDP00005031819_Myripristis_murdjan 709:RIVTDLEERR SQSLQDVALV RKEFA-EYVD KRTGCDLVPL PPRLVSEALG LSQ-KGGGVV 766**

**ENSONIP00000067431_Oreochromis_niloticus 682:RIITDLEERR NQSLQDVALV RREFE-QYVD KRSGCDLVPL PPRLVSEALG LSH-KDGAVV 739**

**ENSNBRP00000029628_Neolamprologus_brichardi 682:QIITDLEERR NQSLQDVALV RREFE-QYVD KRSGCDLVPL PPRLVSEALG LSH-KDGAVV 739**

**ENSHBUP00000030531_Haplochromis_burtoni 651:RIITDLEERR NQSLQDVALV RREFE-QYVD KRSGCDLVPL PPRLVSEALG LSH-KDGAVV 708**

**ENSPNYP00000010231_Pundamilia_nyererei 685:RIITDLEERR NQSLQDVALV RREFE-QYVD KRSGCDLVPL PPRLVSEALG LSH-KDGAVV 742**

**ENSMZEP00005026771_Maylandia_zebra 670:RIITDLEERR NQSLQDVALV RREFE-QYVD KRSGCDLVPL PPRLVSEALG LSH-KDGAVV 727**

**ENSACLP00000035664_Astatotilapia_calliptera 670:RIITDLEERR NQSLQDVALV RREFE-QYVD KRSGCDLVPL PPRLVSEALG LSH-KDGAVV 727**

**ENSLBEP00000024994_Labrus_bergylta 645:RIITDLDERR NHSLQDVALV RKEFS-QYVD KRSGCDLVPL PPRLVSEALG LSH-RDGGVV 702**

**ENSCLMP00005004807_Cyclopterus_lumpus 704:RIVTDLEERR SHSLQDVALV RKEFG-RYVD KQSGCDLVPL PPRLVSEALG LSH-RDGGVV 761**

**ENSCGOP00000000746_Cottoperca_gobio 698:RIVTDLEERR SRSLQDVTLV RKEFQ-QYVD KRSGCDLVPM PPRLVSVALG LSH-RDGDVV 755**

**ENSSPAP00000013334_Stegastes_partitus 679:RIITDLEGRR NQSLQDVALV RKEFG-QYVD KRSGCDLVPL PPRLVSEALG LSH---GAVR 734**

**ENSAPOP00000010991_Acanthochromis_polyacanthus 708:RIITDLEGRR NQSLQDVALV RREFE-QYVD KRSGCDLVPL PPRLVSEALG LSH-MGGAVV 765**

**ENSAPEP00000019384_Amphiprion_percula 709:RIITDLEGRR NQSLQDVALV RKEFG-QYVD KRSGCDLVPL PPRLVSEALG LSH-MGGAVV 766**

**ENSAOCP00000003852_Amphiprion_ocellaris 678:RIITDLEGRR NQSLQDVALV RREFG-QYVD KRSGCDLVPL PPRLVSEALG LSH-MGGAVV 735**

**ENSLCRP00005003692_Larimichthys_crocea 695:RIVTDLEERR SQSLQDVALV RKEFR-HYVD KRSGCDLVPL PPRLVSEALG LSH-RDGDVV 752**

**ENSSMAP00000005463_Scophthalmus_maximus 720:RIITDLEERR SRSLQDVALV RKEFG-QFVD KRTGCDLVPL PPRLVSEALG SSH-RNGGVA 777**

**ENSSLDP00000025094_Seriola_lalandi_dorsalis 727:RIITDLEERR SQSLQDVAVV RKEFG-HYVD KRTGCDLVPL PPRLVSEALG LSH-SNGGVV 784**

**ENSLCAP00010022815_Lates_calcarifer 711:RIITDLEERR SQSLQDVAVV RKEFG-QYVD KRTGCDLVPL PPRLVSEALG LSH-RGGDVV 768**

**ENSSAUP00010027448_Sparus_aurata 721:RVVTDLEERR TQSLHDVAVV RKKFG-QYVD KRSGCDLVPL PQRLVSEALG LSH-SDGGVV 778**

**ENSDLAP00005056549_Dicentrarchus_labrax 711:RIITDLEERR SQSLQDVALV RKEFG-QYVD KRSGSDLVPL PPRLVSEALG LSD-RDGGVV 768**

**(Figure S3: 16/19)**

**EKC36382.1_Crassostrea_gigas 656:-------VPV ITRKEFKYKL HHPE---AKD NPHTAVLRGY RSRKRDEAIA FANERDFMAS 705**

**ENSLLEP00000020779_Leptobrachium_leishanense 758:GHSGRFLLPV ITRKEFKYKT LKPL---AKD NPYNAVLKGY VGRKRDQTIA YMNERHFTAS 814**

**XtCRY6_LC705158 (This_Study) 753:NRTGSFLLPV ITRKEFKYKT LQPD---TKD NPYNTVLKGY VSRKRDETIA YMNERHFTAS 809**

**ENSXLAT00005054664_Xenopus_laevis 745:NRTSSFLLPV ITRKEFKYKT LQPE---AKD NPYSTVLKGY VSRKRDETIA YMNEKHFTAS 801**

**ENSLOCP00000004832_Lepisosteus_oculatus 719:QRGGRFLLPV ITRKEFQHQT LEPG---AQT NPFDAVLRGY VSRQRDEAAA FLRERDFTAS 775**

**ENSSFOP00015030596_Scleropages_formosus 705:DRGGRFLLPL ITRMEFKYQT QNPD---CRD NPFDAVLKGY VSRKRDEATA FRNERDFAAS 761**

**ENSCHAP00000044642_Clupea_harengus 737:RSGSRFLLPV ITRMEFKHQS VEPDAAEGRV NPYNAVLKGY VSRKRDETVA FLNERDFMAS 796**

**ENSAMXP00000003765_Astyanax_mexicanus 696:RGGNEFLLPV ITRMEFRLEL ED-------T ASHSAVLKGY VSRKRDEKIA FLNKCDFTAS 748**

**ENSDARP00000105169_Danio_rerio 704:HEKNGFLLPV ITRMEFKHQS EDPD---RQD NPYSAVLKGY VSRKRDETVA FLNERDFTAS 760**

**ENSSGRP00000063675_Sinocyclocheilus_grahami 705:REGTGFLLPV ITRMEFKHQS EDPD---RQD NPYSAVLKGY VSRKRDETIA FLNERDFTAS 761**

**ENSCARP00000075848_Carassius_auratus/1801 692:REGTGFLLPV ITRMEFKHQS EDPD---RQD NPCSAVLKGY VSRKRDETIA FLNERDFTAS 748**

**ENSCARP00000074480_Carassius_auratus/1814 705:REGTGFLLPV ITRMEFKHQS EDPD---RQD NPCSAVLKGY VSRKRDETIA FLNERDFTAS 761**

**ENSGMOP00000042713_Gadus_morhua 790:TGGEQFLLPV ITRMEFKHRR GDPG-EDAAS NPYDAVLKGY VNRKRDETVA FLNQRDFSAS 848**

**ENSNFUP00015037037_Nothobranchius_furzeri 768:RGGRQFLLPL ITRMEFKYQQ EEPE-ADAAS NPYNATLKGY VSRRRDETVA FLNERDFKTS 826**

**ENSTRUP00000037131_Takifugu_rubripes 755:TGGKQFLLPV ITRMEFKHQQ EDPD-ADAAS NPYNAVLKGY VSRKRDETIA FLNERDFTAS 813**

**ENSKMAP00000018790_Kryptolebias_marmoratus 758:REGKQFLLPL ITRMEFKHQL ENPD-ADAAS NPYNAVLKGY VSRKRDETVA FLNERDFKAS 816**

**ENSCVAP00000003000_Cyprinodon_variegatus 699:TEGKQFLLPV ITRMEFKHQL EDPD-ADAAS NPYNAVLKGY VSRKRDETIA FLNERDFTAS 757**

**ENSPFOP00000018725_Poecilia_formosa 767:TEGKQFLLPV ITRMEFKHQL EDPD-ADAAS NPYNAVLKGY VSRKRDETIA FLNETDFTAS 825**

**ENSFHEP00000001658_Fundulus_heteroclitus 768:TEGKEFLLPV ITRMEFKHQL EDPD-ADAAS NPYNAVLKGY VSRKRDETIA FLNERDFTAS 826**

**ENSOMYP00000024389_Oncorhynchus_mykiss 669:NTGGQFLLPV ITRMEFKHQS DDP---DAAS NPYNAVLKGY VSRRRNETIA FLNQTDFTAS 725**

**ENSOTSP00005065052_Oncorhynchus_tshawytscha 791:NTGGQFLLPV ITRMEFKHQS DDPD-ADAAS NPYNAVLKGY VSRRRNETIA FLNQTDFTAS 849**

**ENSOKIP00005070961_Oncorhynchus_kisutch 790:NTGGQFLLPV ITRMEFKHQS DDPD-ADAAS NPYNAVLKGY VSRRRNETIA FLNQTDFTAS 848**

**ENSHHUP00000077840_Hucho_hucho 788:NTGGQFLLPV ITRMEFKHQS DDPD-ADAAS NPYNAVLKGY VSRRRNETIA FLNQTDFTAS 846**

**ENSSSAP00000016302_Salmo_salar 734:-TGGQFLLPV ITRMEFKHQS DDPD-ADAAS NPYNAVLKGY VSRRRNETIA FLNQTDFTAS 791**

**ENSSTUP00000081428_Salmo_trutta 794:STGGQFLLPV ITRMEFKHQS DDPD-ADAAS NPYNAVLKGY VSRRRNETIA FLNQTDFTAS 852**

**ENSHCOP00000008781_Hippocampus_comes 677:--GKQFLLPV ITRMEFKHQM DDPD-MDAAT NPYNAVLKGY VSRKRDETVA FLNERDFTAS 733**

**ENSOJAP00000007613_Oryzias_javanicus 737:KEGKQFLLPV ITRMEFKYQQ DDPD-ADAAS NPYNAVLKGY VSRKRDETIA FLNQQDFTAS 795**

**ENSORLP00000011698_Oryzias_latipes 744:--GKQFLLPV ITRMEFKYQQ DDP---DAAS NPYNAVLKGY VSRKRDETIA FLNQRDFTAS 798**

**ENSOSIP00000024525_Oryzias_sinensis 744:--GKQFLLPV ITRMEFKYQQ DDP---DAAS NPYNAVLKGY VSRKRDETIA FLNQRDFTAS 798**

**ENSBSLP00000028581_Betta_splendens 710:LEGKQFLLPV ITRMEFKYQQ ENPD-TDAAA NPYNALLKGY VSRKRDETIA FLNERDFTAS 768**

**ENSATEP00000003870_Anabas_testudineus 725:IEGKQFLLPV ITRMEFKYQQ ENPN-ADASS NPYNALLKGY VSRKRDETIA FLNERDFTAS 783**

**ENSMMDP00005031819_Myripristis_murdjan 767:TTGKLFLLPV ITRMEFKHQS DDPD-ADAAS NPYNAVLKGY VSRKRDETIA FLNERDFTAS 825**

**ENSONIP00000067431_Oreochromis_niloticus 740:TQGKQFLLPV ITRMEFKHQQ EDPD-ADATS NPYNAVLKGY VSRKRDETIA FLNERDFTAS 798**

**ENSNBRP00000029628_Neolamprologus_brichardi 740:TQGKQFLLPV ITRMEFKHQH EDPD-ADATS NPYNAVLKGY VSRKRDETIA FLNKRDFTAS 798**

**ENSHBUP00000030531_Haplochromis_burtoni 709:TQGKQFLLPV ITRMEFKHQH EDPD-ADATS NPYNAVLKGY MSRKRDETIA FLNKRDFTAS 767**

**ENSPNYP00000010231_Pundamilia_nyererei 743:TQGKQFLLPV ITRMEFKHQH EDPD-ADATS NPYNAVLKGY VSRKRDETIA FLNKRDFTAS 801**

**ENSMZEP00005026771_Maylandia_zebra 728:TQGKQFLLPV ITRMEFKHQH EDPD-ADATS NPYNAVLKGY VSRKRDETIA FLNKRDFTAS 786**

**ENSACLP00000035664_Astatotilapia_calliptera 728:TQGKQFLLPV ITRMEFKHQH EDPD-ADATS NPYNAVLKGY VSRKRDETIA FLNKRDFTAS 786**

**ENSLBEP00000024994_Labrus_bergylta 703:KEGKQFLLPV ITRMEFKHQL EDPD-ADAAS NPYNAVLKGY VSRKRDETIA FLNERDFTAS 761**

**ENSCLMP00005004807_Cyclopterus_lumpus 762:TEGKHFLLPV ITRMEFKHQL EDP---DATS NPYNAVLKGY VSRKRDETIA FLNERDFTAS 818**

**ENSCGOP00000000746_Cottoperca_gobio 756:TEGKQFLLPV ITRMEFKHQL EDPD-ADAAS NPYNAVLKGY VSRKRDETIA FFNERDFTAS 814**

**ENSSPAP00000013334_Stegastes_partitus 735:TEGKQFLLPV ITRMEFKHQL EDPD-ADAAA NPYNAVLKGY VSRKRDETIA FLNERDFTAS 793**

**ENSAPOP00000010991_Acanthochromis_polyacanthus 766:KEGKQFLLPV ITRMEFKHQL EDPD-ADAAS NPYNAVLKGY VSRKRDETIA FLNERDFTAS 824**

**ENSAPEP00000019384_Amphiprion_percula 767:KEGKEFLLPV ITRMEFKHQL EDPD-ADAAS NPYNAVLKGY VSRKRDETIA FLNERDFTAS 825**

**ENSAOCP00000003852_Amphiprion_ocellaris 736:KEGKEFLLPV ITRMEFKHQL EDPD-ADAAS NPYNAVLKGY VSRKRDETIA FLNERDFTAS 794**

**ENSLCRP00005003692_Larimichthys_crocea 753:TAGTQFLLPV ITRMEFKHQL EDPE-ADAAS NPYNAVLKGY VSRKRDETIA FFNERDFTAS 811**

**ENSSMAP00000005463_Scophthalmus_maximus 778:AEAKQFLLPV ITRMEFKHQL EDPD-ADAAS NPYNAVLKGY VSRKRDETIA FLNERDFTAS 836**

**ENSSLDP00000025094_Seriola_lalandi_dorsalis 785:AEGKQFLLPV ITRMEFKHQL EDPD-ADAAS NPYNAVLKGY VSRKRDETIA FLNERDFTAS 843**

**ENSLCAP00010022815_Lates_calcarifer 769:AEGKQFLLPV ITRMEFKHQL EDPD-ADAAS NPYNAVLKGY VSRKRDETIA FLNERDFTAS 827**

**ENSSAUP00010027448_Sparus_aurata 779:TTGKQFLLPV ITRMEFKHQL EDPD-ADAAS NPYNAVLKGY VSRKRDETIA FLNERDFTTS 837**

**ENSDLAP00005056549_Dicentrarchus_labrax 769:TAGKQFLLPV ITRMEFKHQL EDPD-ADAAS NPYNAVLKGY VSRKRDETIA FLNERDFTAS 827**

**(Figure S3: 17/19)**

**EKC36382.1_Crassostrea_gigas 706:AMNERETYPN RVISD---LE GAREQSLTDV VNVRKKHPEF VDRRTGNDLV PLP-DGLCVP 761**

**ENSLLEP00000020779_Leptobrachium_leishanense 815:ALNETAQMYD RRERTARLLE GLP-PSTD-- KTERRRIPA- SDR-----LP VIP-PAYL-- 862**

**XtCRY6_LC705158 (This_Study) 810:TINEGAQRHE RIERTNRLME GLPAPSDA-- KNKSRRTPK- KDP-----FS IIP-PSYL-- 858**

**ENSXLAT00005054664_Xenopus_laevis 802:TIHEGAQRHE RTERTSRLME GLPAPSEP-- KNKSRRTPR- KDP-----FS IIP-PSYL-- 850**

**ENSLOCP00000004832_Lepisosteus_oculatus 776:VMSEGGRRLE RQERDRRVLE GLPPPPAQ-- KSRARRTPR- TDP-----FS VVP-GGAP-- 824**

**ENSSFOP00015030596_Scleropages_formosus 762:VLSESVQRRE RQEREQRALD GIPRPA---- TGRPGRTPR- KDV-----FS AQG-AGFT-- 808**

**ENSCHAP00000044642_Clupea_harengus 797:VVHEGTQRLE RAERNQRLLE GLPAPPSA-T RGRPRRTPTM PQR------- ---------- 838**

**ENSAMXP00000003765_Astyanax_mexicanus 749:VMYENAQRRE RMEKNHCMLE DLQCPPTN-- RGRGRHRNSR ---------- --P-VSGA-- 791**

**ENSDARP00000105169_Danio_rerio 761:VMCESAQRRE RLERDSCLLE GLPRPTAS-- RGGARRTQT- RDP-----YS KVP-GGVA-- 809**

**ENSSGRP00000063675_Sinocyclocheilus_grahami 762:VMCENTQRRE RLERDWCLLE GLPQPIVS-- RGRARRTPT- RDP-----YS KVP-GGVA-- 810**

**ENSCARP00000075848_Carassius_auratus/1801 749:VMCENTQRRE RLERDWCLLE GLPKPTVS-- RGRARRTPT- RDP-----YS KVP-GGVA-- 797**

**ENSCARP00000074480_Carassius_auratus/1814 762:VMCENTQRRE RLERDWCLLE GLPKPTVS-- RGRARRTPT- RDP-----YS KVP-GGVA-- 810**

**ENSGMOP00000042713_Gadus_morhua 849:VMNEGAQRRE RLDSDMRILE GLPRAPPG-- RGRARPTPAA QGQ-----TS ---------- 891**

**ENSNFUP00015037037_Nothobranchius_furzeri 827:VMYESAQRQG RLERDYRRIE GLPSSPSP-- RGRARRTPTA KDR-----FS IVP-GGAV-- 876**

**ENSTRUP00000037131_Takifugu_rubripes 814:VMHEGAQRRE RMESDQRRLE GLPRGPAP-- RGRVRRTPTA KDR-----FS VVP-GGAV-- 863**

**ENSKMAP00000018790_Kryptolebias_marmoratus 817:VMCEGAQRRE RLERDYRRME GLPPAPSP-- RGRGRRTPTA KDR-----FS VVP-GGSV-- 866**

**ENSCVAP00000003000_Cyprinodon_variegatus 758:VMYEGAQRRE RLERDHRRLE GLPPPPSA-- RGKARRTPTA KDR-----FS VVP-GGAI-- 807**

**ENSPFOP00000018725_Poecilia_formosa 826:VMYEGAQRQE RLERDYRRME GLPPAPSN-- RGRARRTPTA KDK-----FS VIP-GGTI-- 875**

**ENSFHEP00000001658_Fundulus_heteroclitus 827:VMYEGAQRQE RLERDRRRME GLPPAPSP-- RGRARRTPTA KDK-----FS IVP-GGAI-- 876**

**ENSOMYP00000024389_Oncorhynchus_mykiss 726:VINEGAERRE RQEQDQRRME GLPRPLAV-- ERRGKRTPAA KDR-----FS TVP-GGVA-- 775**

**ENSOTSP00005065052_Oncorhynchus_tshawytscha 850:VINEGAERRE RQEQDQRRME GLPRPLAV-- EQRGKRTPAA KDR-----FS TVP-GRVA-- 899**

**ENSOKIP00005070961_Oncorhynchus_kisutch 849:VINEGAERRE RQEQDQRRME GLPHPLAV-- ERRGKRTPAA KDR-----VS TVP-GRVP-- 898**

**ENSHHUP00000077840_Hucho_hucho 847:VINEGAERRD RQERDQRRME GLPRPLA--- -GRGRETTAA KDR-----FS TVP-GGVA-- 894**

**ENSSSAP00000016302_Salmo_salar 792:VINEGTERRE RQERDQRRME GLPRPLAA-- QGRGKRTPAA KDR-----FS TVP-GGVA-- 841**

**ENSSTUP00000081428_Salmo_trutta 853:VINEGTERRE RQERDQRRME GLPRPLAA-- QGRGKRTPAA KDR-----FS TVP-GGVA-- 902**

**ENSHCOP00000008781_Hippocampus_comes 734:VLHEGAQRRE RLESDHRRME GLPPKRLP-T QGRARRSTNT NDR-----FS ILP-SGTVL- 785**

**ENSOJAP00000007613_Oryzias_javanicus 796:VMYEGAQRRE RLERDYRRME GLPPPPSA-- RGRARRTPTA KDR-----FS IVP-GGVV-- 845**

**ENSORLP00000011698_Oryzias_latipes 799:VMHEGTQRLE RLERDYRRME GLPPPPST-- QGRARRTPTA KDR-----FS IVP-GGAV-- 848**

**ENSOSIP00000024525_Oryzias_sinensis 799:VMHEGAQRRE RLERDYRRME GLPPPPST-- QGRARRTPTA KDR-----FS IVP-GGVV-- 848**

**ENSBSLP00000028581_Betta_splendens 769:VMYEAVQRKE RLESDHRRME GLPEPPAL-- RGKVRRTPTA KDK-----FS IVPGGGAV-- 819**

**ENSATEP00000003870_Anabas_testudineus 784:VMFEAVQRKE RLESDHRRIE GLPHAPAP-- RGSARRTPTA KDK-----FS IVP-GGAL-- 833**

**ENSMMDP00005031819_Myripristis_murdjan 826:VMYEGAQRRE RLESDYRRME GLPRPSAP-- RGRARRTPTA KDR-----FS IVP-GGVV-- 875**

**ENSONIP00000067431_Oreochromis_niloticus 799:VMHEAIQRKE RLNSDYRRME GLPPSPSP-- RGRARRTPTA KDR-----FS IVP-GGAV-- 848**

**ENSNBRP00000029628_Neolamprologus_brichardi 799:VMHEAVQRKE RLNSDYRRME GLPPSPSP-- RGRARRTPTA KDR-----FS IVP-GGAV-- 848**

**ENSHBUP00000030531_Haplochromis_burtoni 768:VMHEAVQRKE RLNSDYRRME GLSPSPSP-- RGRARRTPTG KDR-----FS IVP-GGAV-- 817**

**ENSPNYP00000010231_Pundamilia_nyererei 802:VMHEAVQRKE RLNSDYRRME GLPPSPSP-- RGRARRTPTA KDR-----FS IVP-GGAV-- 851**

**ENSMZEP00005026771_Maylandia_zebra 787:VMHEAVQRKE RLNSDYRRME GLPPSPSP-- RGRARRTPTA KDR-----FS IVP-GGAV-- 836**

**ENSACLP00000035664_Astatotilapia_calliptera 787:VMHEAVQRKE RLNSDYRRME GLPPSPSP-- RGRARRTPTA EDR-----FS IVP-GGAV-- 836**

**ENSLBEP00000024994_Labrus_bergylta 762:VMYEGTQRRE RLESDYRRME GLPTPPAA-- RGRARRTPTA KDR-----FS VVP-GGAV-- 811**

**ENSCLMP00005004807_Cyclopterus_lumpus 819:VMYEGVQRKE RLESNYRRIE GLPQPPAP-- RGRARRTPTA KDR-----FP VVP-SGSV-- 868**

**ENSCGOP00000000746_Cottoperca_gobio 815:VMNEGVQRKE RLESDYRRIE GLPPSPAP-- RGRARRTPTA KDR-----FS VVP-GGAV-- 864**

**ENSSPAP00000013334_Stegastes_partitus 794:VMYEGTQRKE RLESDYRRME GLPLLPSP-- RGRARRTPTA KDR-----FS VVP-GGAV-- 843**

**ENSAPOP00000010991_Acanthochromis_polyacanthus 825:VMYEGAQRRE RLESDYRRME GLPLLPSP-- RGRARRTPTA KDR-----FS IVP-GGAV-- 874**

**ENSAPEP00000019384_Amphiprion_percula 826:VMYEGAQRRE RLESDYRRME GLPLLPSP-- RGRVRRTPTA KDR-----FS IVP-GGAV-- 875**

**ENSAOCP00000003852_Amphiprion_ocellaris 795:VMYEGAQRRE RLESDYRRME GLPLLPSP-- RGRVRRTPTA KDR-----FS IVP-GGAV-- 844**

**ENSLCRP00005003692_Larimichthys_crocea 812:VMYEGTQRKE RLENDYRRME GLSQLPAP-- RGRARRTPTA NDR-----FS IVP-GGAV-- 861**

**ENSSMAP00000005463_Scophthalmus_maximus 837:VMYEGTQRKE RLESDYRRME GLPTPPAP-- QGRARRTPKA KDK-----FS LVP-GGVV-- 886**

**ENSSLDP00000025094_Seriola_lalandi_dorsalis 844:VMYEGAQRKE RLEKDYRRME GLPQPAAP-- RGRARRTPTA KDR-----FS IVP-GGAV-- 893**

**ENSLCAP00010022815_Lates_calcarifer 828:VMYEGTQRRE RLESDQRRME GLPRPPAP-- RGRARRTPTA KDR-----FS IVP-GGVV-- 877**

**ENSSAUP00010027448_Sparus_aurata 838:VMYEGTQRRE RLESDYRRME GLPRPAAP-- RGRARRTPIA KDR-----FS VVP-GGAV-- 887**

**ENSDLAP00005056549_Dicentrarchus_labrax 828:VMYEGTQRKE RLESDYRRME GLPQPPAP-- RGRARRTPTA KDR-----FS IVP-GGAV-- 877**

**(Figure S3: 18/19)**

**EKC36382.1_Crassostrea_gigas 762:VITRKEFKYK LHHPEAKDNP HTAVLRGYRS RKRDEAIAFA NERDFMASAM NESVKLSERR 821**

**ENSLLEP00000020779_Leptobrachium_leishanense 862:---------- ---------- ---------- ---------- ---------- ---------- 862**

**XtCRY6_LC705158 (This_Study) 858:---------- ---------- ---------- ---------- ---------- ---------- 858**

**ENSXLAT00005054664_Xenopus_laevis 850:---------- ---------- ---------- ---------- ---------- ---------- 850**

**ENSLOCP00000004832_Lepisosteus_oculatus 824:---------- ---------- ---------- ---------- ---------- ---------- 824**

**ENSSFOP00015030596_Scleropages_formosus 808:---------- ---------- ---------- ---------- ---------- ---------- 808**

**ENSCHAP00000044642_Clupea_harengus 838:---------- ---------- ---------- ---------- ---------- ---------- 838**

**ENSAMXP00000003765_Astyanax_mexicanus 791:---------- ---------- ---------- ---------- ---------- ---------- 791**

**ENSDARP00000105169_Danio_rerio 809:---------- ---------- ---------- ---------- ---------- ---------- 809**

**ENSSGRP00000063675_Sinocyclocheilus_grahami 810:---------- ---------- ---------- ---------- ---------- ---------- 810**

**ENSCARP00000075848_Carassius_auratus/1801 797:---------- ---------- ---------- ---------- ---------- ---------- 797**

**ENSCARP00000074480_Carassius_auratus/1814 810:---------- ---------- ---------- ---------- ---------- ---------- 810**

**ENSGMOP00000042713_Gadus_morhua 891:---------- ---------- ---------- ---------- ---------- ---------- 891**

**ENSNFUP00015037037_Nothobranchius_furzeri 876:---------- ---------- ---------- ---------- ---------- ---------- 876**

**ENSTRUP00000037131_Takifugu_rubripes 863:---------- ---------- ---------- ---------- ---------- ---------- 863**

**ENSKMAP00000018790_Kryptolebias_marmoratus 866:---------- ---------- ---------- ---------- ---------- ---------- 866**

**ENSCVAP00000003000_Cyprinodon_variegatus 807:---------- ---------- ---------- ---------- ---------- ---------- 807**

**ENSPFOP00000018725_Poecilia_formosa 875:---------- ---------- ---------- ---------- ---------- ---------- 875**

**ENSFHEP00000001658_Fundulus_heteroclitus 876:---------- ---------- ---------- ---------- ---------- ---------- 876**

**ENSOMYP00000024389_Oncorhynchus_mykiss 775:---------- ---------- ---------- ---------- ---------- ---------- 775**

**ENSOTSP00005065052_Oncorhynchus_tshawytscha 899:---------- ---------- ---------- ---------- ---------- ---------- 899**

**ENSOKIP00005070961_Oncorhynchus_kisutch 898:---------- ---------- ---------- ---------- ---------- ---------- 898**

**ENSHHUP00000077840_Hucho_hucho 894:---------- ---------- ---------- ---------- ---------- ---------- 894**

**ENSSSAP00000016302_Salmo_salar 841:---------- ---------- ---------- ---------- ---------- ---------- 841**

**ENSSTUP00000081428_Salmo_trutta 902:---------- ---------- ---------- ---------- ---------- ---------- 902**

**ENSHCOP00000008781_Hippocampus_comes 785:---------- ---------- ---------- ---------- ---------- ---------- 785**

**ENSOJAP00000007613_Oryzias_javanicus 845:---------- ---------- ---------- ---------- ---------- ---------- 845**

**ENSORLP00000011698_Oryzias_latipes 848:---------- ---------- ---------- ---------- ---------- ---------- 848**

**ENSOSIP00000024525_Oryzias_sinensis 848:---------- ---------- ---------- ---------- ---------- ---------- 848**

**ENSBSLP00000028581_Betta_splendens 819:---------- ---------- ---------- ---------- ---------- ---------- 819**

**ENSATEP00000003870_Anabas_testudineus 833:---------- ---------- ---------- ---------- ---------- ---------- 833**

**ENSMMDP00005031819_Myripristis_murdjan 875:---------- ---------- ---------- ---------- ---------- ---------- 875**

**ENSONIP00000067431_Oreochromis_niloticus 848:---------- ---------- ---------- ---------- ---------- ---------- 848**

**ENSNBRP00000029628_Neolamprologus_brichardi 848:---------- ---------- ---------- ---------- ---------- ---------- 848**

**ENSHBUP00000030531_Haplochromis_burtoni 817:---------- ---------- ---------- ---------- ---------- ---------- 817**

**ENSPNYP00000010231_Pundamilia_nyererei 851:---------- ---------- ---------- ---------- ---------- ---------- 851**

**ENSMZEP00005026771_Maylandia_zebra 836:---------- ---------- ---------- ---------- ---------- ---------- 836**

**ENSACLP00000035664_Astatotilapia_calliptera 836:---------- ---------- ---------- ---------- ---------- ---------- 836**

**ENSLBEP00000024994_Labrus_bergylta 811:---------- ---------- ---------- ---------- ---------- ---------- 811**

**ENSCLMP00005004807_Cyclopterus_lumpus 868:---------- ---------- ---------- ---------- ---------- ---------- 868**

**ENSCGOP00000000746_Cottoperca_gobio 864:---------- ---------- ---------- ---------- ---------- ---------- 864**

**ENSSPAP00000013334_Stegastes_partitus 843:---------- ---------- ---------- ---------- ---------- ---------- 843**

**ENSAPOP00000010991_Acanthochromis_polyacanthus 874:---------- ---------- ---------- ---------- ---------- ---------- 874**

**ENSAPEP00000019384_Amphiprion_percula 875:---------- ---------- ---------- ---------- ---------- ---------- 875**

**ENSAOCP00000003852_Amphiprion_ocellaris 844:---------- ---------- ---------- ---------- ---------- ---------- 844**

**ENSLCRP00005003692_Larimichthys_crocea 861:---------- ---------- ---------- ---------- ---------- ---------- 861**

**ENSSMAP00000005463_Scophthalmus_maximus 886:---------- ---------- ---------- ---------- ---------- ---------- 886**

**ENSSLDP00000025094_Seriola_lalandi_dorsalis 893:---------- ---------- ---------- ---------- ---------- ---------- 893**

**ENSLCAP00010022815_Lates_calcarifer 877:---------- ---------- ---------- ---------- ---------- ---------- 877**

**ENSSAUP00010027448_Sparus_aurata 887:---------- ---------- ---------- ---------- ---------- ---------- 887**

**ENSDLAP00005056549_Dicentrarchus_labrax 877:---------- ---------- ---------- ---------- ---------- ---------- 877**

**(Figure S3: 19/19)**

**EKC36382.1_Crassostrea_gigas 822:LKATQYEAL 830**

**ENSLLEP00000020779_Leptobrachium_leishanense 863:-----HLSN 866**

**XtCRY6_LC705158 (This_Study) 859:-----HLAN 862**

**ENSXLAT00005054664_Xenopus_laevis 851:-----HLAN 854**

**ENSLOCP00000004832_Lepisosteus_oculatus 825:-----ITPS 828**

**ENSSFOP00015030596_Scleropages_formosus 809:-----LLHN 812**

**ENSCHAP00000044642_Clupea_harengus 838:--------- 838**

**ENSAMXP00000003765_Astyanax_mexicanus 792:-----AQQR 795**

**ENSDARP00000105169_Danio_rerio 810:-----VPRK 813**

**ENSSGRP00000063675_Sinocyclocheilus_grahami 811:-----VPHR 814**

**ENSCARP00000075848_Carassius_auratus/1801 798:-----VPHR 801**

**ENSCARP00000074480_Carassius_auratus/1814 811:-----VPHR 814**

**ENSGMOP00000042713_Gadus_morhua 891:--------- 891**

**ENSNFUP00015037037_Nothobranchius_furzeri 877:-----TLLK 880**

**ENSTRUP00000037131_Takifugu_rubripes 864:-----ASLR 867**

**ENSKMAP00000018790_Kryptolebias_marmoratus 867:-----TSVK 870**

**ENSCVAP00000003000_Cyprinodon_variegatus 808:-----VSKN 811**

**ENSPFOP00000018725_Poecilia_formosa 876:-----ASRN 879**

**ENSFHEP00000001658_Fundulus_heteroclitus 877:-----ASTN 880**

**ENSOMYP00000024389_Oncorhynchus_mykiss 776:-----TSHR 779**

**ENSOTSP00005065052_Oncorhynchus_tshawytscha 900:-----TSHR 903**

**ENSOKIP00005070961_Oncorhynchus_kisutch 899:-----TSHR 902**

**ENSHHUP00000077840_Hucho_hucho 895:-----TSHR 898**

**ENSSSAP00000016302_Salmo_salar 842:-----TSHR 845**

**ENSSTUP00000081428_Salmo_trutta 903:-----TSHR 906**

**ENSHCOP00000008781_Hippocampus_comes 786:-----TTHR 789**

**ENSOJAP00000007613_Oryzias_javanicus 846:-----TS-- 847**

**ENSORLP00000011698_Oryzias_latipes 849:-----TS-- 850**

**ENSOSIP00000024525_Oryzias_sinensis 849:-----TS-- 850**

**ENSBSLP00000028581_Betta_splendens 820:-----TSFE 823**

**ENSATEP00000003870_Anabas_testudineus 834:-----TSLR 837**

**ENSMMDP00005031819_Myripristis_murdjan 876:-----TSPR 879**

**ENSONIP00000067431_Oreochromis_niloticus 849:-----TSLK 852**

**ENSNBRP00000029628_Neolamprologus_brichardi 849:-----TSLK 852**

**ENSHBUP00000030531_Haplochromis_burtoni 818:-----TSLK 821**

**ENSPNYP00000010231_Pundamilia_nyererei 852:-----TSLK 855**

**ENSMZEP00005026771_Maylandia_zebra 837:-----TSLK 840**

**ENSACLP00000035664_Astatotilapia_calliptera 837:-----TSLK 840**

**ENSLBEP00000024994_Labrus_bergylta 812:-----TSLR 815**

**ENSCLMP00005004807_Cyclopterus_lumpus 869:-----SSVR 872**

**ENSCGOP00000000746_Cottoperca_gobio 865:-----SSLR 868**

**ENSSPAP00000013334_Stegastes_partitus 844:-----TSLR 847**

**ENSAPOP00000010991_Acanthochromis_polyacanthus 875:-----TSLK 878**

**ENSAPEP00000019384_Amphiprion_percula 876:-----TSLK 879**

**ENSAOCP00000003852_Amphiprion_ocellaris 845:-----TSLK 848**

**ENSLCRP00005003692_Larimichthys_crocea 862:-----TSLR 865**

**ENSSMAP00000005463_Scophthalmus_maximus 887:-----TSLR 890**

**ENSSLDP00000025094_Seriola_lalandi_dorsalis 894:-----TSLR 897**

**ENSLCAP00010022815_Lates_calcarifer 878:-----ASLR 881**

**ENSSAUP00010027448_Sparus_aurata 888:-----TSLR 891**

**ENSDLAP00005056549_Dicentrarchus_labrax 878:-----TSLR 881**

**Figure S3 Alignment of CRY6s by MAFFT (**[**https://mafft.cbrc.jp/alignment/server/**](https://mafft.cbrc.jp/alignment/server/)**)**

DI-UIM and putative NoLS of XtCRY6 are colored in red. NoLS motifs ([R/K][R/K]X[R/K]) are highlighted.
